# Supplementary material for: Circulating Protein Biomarkers for Prognostic Use in Patients with Advanced Pancreatic Ductal Adenocarcinoma Undergoing Chemotherapy
Source: Cancers (Basel). 2022 Jul 1;14(13):3250. doi: 10.3390/cancers14133250 (PMC9264968; doi:10.3390/cancers14133250)
Supplement: Supplementary file 1 [file cancers-14-03250-s001.zip › cancers-1760616-supplementary.pdf]

Supplementary File S1: Supplementary methods

Circulating Protein Biomarkers for Prognostic Use in Patients with advanced Pancreatic Ductal Adenocarcinoma Undergoing Chemotherapy

Sidsel C. Lindgaard[1,*], Emil Maag[2,^], Zsófia Sztupinszki[3,^], Inna M. Chen[1], Astrid Z. Johansen[1], Benny V. Jensen[1], Stig E. Bojesen[4,5], Dorte L. Nielsen[1, 5], Zoltan Szallasi[2,6], Julia S. Johansen[1,5,7].

The REMARK Checklist [25]

	Item to be reported	Page no.
**INTRODUCTION**		
1	State the marker examined, the study objectives, and any pre-specified hypotheses.	4
**MATERIALS AND METHODS**		
Patients		
2	Describe the characteristics (e.g., disease stage or co-morbidities) of the study patients, including their source and inclusion and exclusion criteria.	5 + Table 1
3	Describe treatments received and how chosen (e.g., randomized or rule-based).	Suppl. p. 4
Specimen characteristics		
4	Describe type of biological material used (including control samples) and methods of preservation and storage.	Suppl. p. 4
Assay methods		
5	Specify the assay method used and provide (or reference) a detailed protocol, including specific reagents or kits used, quality control procedures, reproducibility assessments, quantitation methods, and scoring and reporting protocols. Specify whether and how assays were performed blinded to the study endpoint.	Suppl. p. 5-6
Study design		
6	State the method of case selection, including whether prospective or retrospective and whether stratification or matching (e.g., by stage of disease or age) was used. Specify the time period from which cases were taken, the end of the follow-up period, and the median follow-up time.	5 + Suppl. p. 4
7	Precisely define all clinical endpoints examined.	NA
8	List all candidate variables initially examined or considered for inclusion in models.	Table 1 and Table S1
9	Give rationale for sample size; if the study was designed to detect a specified effect size, give the target power and effect size.	6 + Suppl. p.4
Statistical analysis methods		
10	Specify all statistical methods, including details of any variable selection procedures and other model-building issues, how model assumptions were verified, and how missing data were handled.	6 + Suppl. p. 6–14

11	Clarify how marker values were handled in the analyses; if relevant, describe methods used for cut-point determination.	Suppl. p. 6–14
**RESULTS**		
Data		
12	Describe the flow of patients through the study, including the number of patients included in each stage of the analysis (a diagram may be helpful) and reasons for dropout. Specifically, both overall and for each subgroup extensively examined report the numbers of patients and the number of events.	7–12 + Suppl. p. 15–17
13	Report distributions of basic demographic characteristics (at least age and sex), standard (disease-specific) prognostic variables, and tumor marker, including numbers of missing values.	Table 1
Analysis and presentation		
14	Show the relation of the marker to standard prognostic variables.	No standard prognostic variables available
15	Present univariable analyses showing the relation between the marker and outcome, with the estimated effect (e.g., hazard ratio and survival probability). Preferably provide similar analyses for all other variables being analyzed. For the effect of a tumor marker on a time-to-event outcome, a Kaplan-Meier plot is recommended.	Table 4, Figure S1, S2, S3
16	For key multivariable analyses, report estimated effects (e.g., hazard ratio) with confidence intervals for the marker and, at least for the final model, all other variables in the model.	Table 4
17	Among reported results, provide estimated effects with confidence intervals from an analysis in which the marker and standard prognostic variables are included, regardless of their statistical significance.	7–9
18	If done, report results of further investigations, such as checking assumptions, sensitivity analyses, and internal validation.	NA
**DISCUSSION**		
19	Interpret the results in the context of the pre-specified hypotheses and other relevant studies; include a discussion of limitations of the study.	12–15
20	Discuss implications for future research and clinical value.	15

The TRIPOD Checklist

Section/Topic	Item	Checklist Item	Page
**Title and abstract**			
Title	1	Identify the study as developing and/or validating a multivariable prediction model, the target population, and the outcome to be predicted.	1
Abstract	2	Provide a summary of objectives, study design, setting, participants, sample size, predictors, outcome, statistical analysis, results, and conclusions.	3
**Introduction**			
Background and objectives	3a	Explain the medical context (including whether diagnostic or prognostic) and rationale for developing or validating the multivariable prediction model, including references to existing models.	4–5
	3b	Specify the objectives, including whether the study describes the development or validation of the model or both.	4, 5, 6
**Methods**			
Source of data	4a	Describe the study design or source of data (e.g., randomized trial, cohort, or registry data), separately for the development and validation data sets, if applicable.	5
	4b	Specify the key study dates, including start of accrual; end of accrual; and, if applicable, end of follow-up.	5

Participants	5a	Specify key elements of the study setting (e.g., primary care, secondary care, general population) including number and location of centres.	5
	5b	Describe eligibility criteria for participants.	5
	5c	Give details of treatments received, if relevant.	Suppl. p. 4
Outcome	6a	Clearly define the outcome that is predicted by the prediction model, including how and when assessed.	Suppl. p. 6–14
	6b	Report any actions to blind assessment of the outcome to be predicted.	NA
Predictors	7a	Clearly define all predictors used in developing or validating the multivariable prediction model, including how and when they were measured.	Table S1, Suppl. p. 5–6
	7b	Report any actions to blind assessment of predictors for the outcome and other predictors.	NA
Sample size	8	Explain how the study size was arrived at.	6, Suppl. p .4
Missing data	9	Describe how missing data were handled (e.g., complete-case analysis, single imputation, multiple imputation) with details of any imputation method.	6 + Suppl. p. 6
Statistical analysis methods	10a	Describe how predictors were handled in the analyses.	6 + Suppl. p. 6–14
	10b	Specify type of model, all model-building procedures (including any predictor selection), and method for internal validation.	6 +Suppl. p. 6–14
	10d	Specify all measures used to assess model performance and, if relevant, to compare multiple models.	6 + Suppl. p. 6–14
Risk groups	11	Provide details on how risk groups were created, if done.	NA
**Results**			
Participants	13a	Describe the flow of participants through the study, including the number of participants with and without the outcome and, if applicable, a summary of the follow-up time. A diagram may be helpful.	Table 2
	13b	Describe the characteristics of the participants (basic demographics, clinical features, available predictors), including the number of participants with missing data for predictors and outcome.	Table 1
Model development	14a	Specify the number of participants and outcome events in each analysis.	Table 2 and througho ut
	14b	If done, report the unadjusted association between each candidate predictor and outcome.	Table S2
Model specification	15a	Present the full prediction model to allow predictions for individuals (i.e., all regression coefficients, and model intercept or baseline survival at a given time point).	Figure 1
	15b	Explain how to the use the prediction model.	NA
Model performance	16	Report performance measures (with CIs) for the prediction model.	Figure 1, table S6
**Discussion**			
Limitations	18	Discuss any limitations of the study (such as nonrepresentative sample, few events per predictor, missing data).	14–15
Interpretation	19b	Give an overall interpretation of the results, considering objectives, limitations, and results from similar studies, and other relevant evidence.	12–15

Implications	20	Discuss the potential clinical use of the model and implications for future research.	15
**Other information**			
Supplementary information	21	Provide information about the availability of supplementary resources, such as study protocol, Web calculator, and data sets.	Suppl.
Funding	22	Give the source of funding and the role of the funders for the present study.	15

Patients

Patients were chosen from the BIOPAC study according to available unthawed serum samples. The patients received one of three possible first-line chemotherapy regimens according to the physician's choice: mFOLFIRINOX (n = 98), gemcitabine + nab-paclitaxel (n = 82), or monotherapy gemcitabine (n = 183). One-hundred and twelve patients (30.9%) received second-line chemotherapy, and thirteen patients (3.6%) went on to receive third-line chemotherapy. All 363 patients had baseline blood samples obtained before the first cycle of first-line palliative chemotherapy, and a subset had longitudinal blood samples obtained before the second treatment cycle (n = 207) and at the time of the first CT scan, at approximately 3 months after treatment initiation (n = 177). For the patients with available longitudinal samples, 111 received gemcitabine as first-line chemotherapy, 48 received gemcitabine + nab-paclitaxel, and 76 received mFOLFIRINOX.

Blood sample characteristics

Blood for analysis was collected a median of 21 days (interquartile range (IQR) 16–31 days) after diagnosis of PDAC and within 0–3 days (IQR) before the start of first-line chemotherapy. The blood samples were drawn into an 8 ml tube (VACUETTE® TUBE 8 ml CAT Serum Separator Clot

Activator). According to the standard operating procedure (SOP) of the BIOPAC biomarker protocol, the tubes were stored at room temperature between 30 and 120 minutes before they were centrifuged at 2300 g at 4˚C for 10 minutes. Samples were then aliquoted into Greiner tubes (Cryo.s™ Freezing Tubes, 2 ml, GR-121280, Greiner Bio-One GmbH) and subsequently stored at −80^{0}C until analysis. Upon collection for this study, the samples were thawed at room temperature, mixed using a vortex mixer, and centrifuged at 3800 rpm for 10 minutes. Then, 250 µL was aliquoted to tubes (2.0 ml graduated screw tubes without ribs, natural, from SSIbio, CA, USA), labelled with an individual number, and stored at −80^{0}C until the analysis of the 92 proteins at BioXpedia, Aarhus, Denmark.

CA19-9

The concentrations of CA19-9 were determined using the Immulite 2000 GI-MA assay (Siemens, Catalogue Number L2KG12): a solid-phase, two-site sequential chemiluminescent immunometric assay. Imprecision was monitored using two internal controls at 16 and 83 kU/L, with coefficients of variation of 8% and 9%. Accuracy was monitored within the standard UK NEQAS program. Elevated CA19-9 was defined as >37 kU/L.

Olink Proximity Extension Assay

We analysed 737 serum samples from 363 PDAC patients. The samples were randomized across 14 Olink PEA plates during the period of October 23, 2018 to April 26, 2019, and normalized for any plate effects using the built-in inter-plate controls according to the manufacturer's

recommendations. Furthermore, eight patient samples from the first five plates analysed before the rest of the plates were included on all subsequent plates for bridging purposes.

Using a proximity extension assay (PEA), 1 µl of serum was mixed with pairs of antibodies linked to oligonucleotides (probes). Upon binding to the target antigen, the probes are brought into proximity with each other, leading to extension of the oligonucleotides by DNA polymerase. This acts as a surrogate marker for the specific antigen, and can then be quantified using real-time PCR (qPCR), whereby the number of PCR copies is proportional to the initial concentration of antigen in the sample [21].

PEA gives abundance levels for each protein measured as NPX values (Normalized Protein eXpression) on a log2 scale. Each assay has an experimentally determined lower limit of detection (LOD) defined as three standard deviations above the background level, determined by the negative controls included on all plates. The standard deviations are assay-specific and estimated during product validation for every panel. For assay values below LOD, the actual value was used. This was chosen because the LOD is considered quite a conservative measurement, leaving a high probability that the value below the LOD is the real value. Additionally, this gives a less skewed distribution compared to replacing data below LOD with a value. Samples with minor quality-control deviations were normalized and included in the analysis. Samples with major deviations were excluded from normalization and data analysis. Assay characteristics including detection limits calculations, assay performance, and validations are available from the manufacturer (www.olink.com).

The analyses were performed at BioXpedia, Aarhus, Denmark, and were performed blinded to the study endpoint, because no research questions were revealed before all samples had been analysed.

IL-6

Serum levels of IL-6 were determined in duplicates using a commercial two-site sandwich-type enzyme-linked immunosorbent assay (ELISA) (R&D Systems® high sensitive IL-6 Catalogue number HS600, Abingdon, Oxon, United Kingdom). The detection limit was 0.01 ng/L. Missing values for IL-6 ELISA (1.3% in the replication cohort) were imputed using the function *impute.knn* from the R package *impute*. These results were used as an extra control for the Olink Proximity Extension Assay.

Statistics

First statistical approach:

Missing values for CA19-9 (13 samples, 6.8%) were imputed using the function *impute.knn* from the R package *impute*. Before imputation, the abundance levels of CA19-9 were scaled to unit variance and centred to have a mean equal to zero.

Differential expression

Differential expression was tested using a *t*-test for independent samples. For all the *t*-tests, it was first checked whether the two groups that were tested were normally distributed using the Shapiro–Wilk test. If one of the groups was not normally distributed, a Wilcoxon rank-sum test was conducted instead. Furthermore, the fold change was calculated on a linear scale as the geometric mean of the first group (survival ≤180 days) divided by the geometric mean of the second group (survival >180 days).

Model generation

All patients were divided into a discovery (n=243) and a replication cohort (n=120). The discovery cohort was further divided into a training (50%) and a test set (50%). Logistic elastic-net (LASSO and Ridge) regression models were used for model building for the exploration of prognostic potential. The first aim was to identify a protein signature with the ability to distinguish patients with advanced PDAC according to survival. Four comparisons were made, with the following cut-off points of OS: ≤90 days vs. >90 days, ≤180 days vs. >180 days, <90 days vs. >1 year, and <90 days vs. >2 years. LASSO regression was used to investigate the stability of the proteins and generate a stability score for each protein. Protein signatures were generated based on the stability scores. For each protein signature, a Ridge regression model was fitted to the training set of the discovery cohort and tested in the corresponding test set. The same procedure was carried out with the entire discovery cohort as the training set and the entire replication cohort as the test set.

In detail, the strategy breaks down into the following steps:

1. **Bootstrap procedure for the discovery cohort**

   In the first step, the samples in the discovery cohort were randomly split into two equally large parts, thus generating a training set and a test set. Using the complete set of differentially expressed proteins mentioned in the introduction, a logistic LASSO regression model was fitted on the training set using the R package *glmnet* with *alpha* = 1 and optimized with the function *cv.glmnet* using 10-fold cross validation in the training set. The fitted logistic LASSO regression model was then employed on the test set. This process was repeated 100 times, thus generating 100 different logistic LASSO regression models.

2. **Logistic LASSO and Ridge regression**

Logistic LASSO regression is a multivariate logistic regression model with an added regularization parameter. When the coefficients of the predictors are estimated in the logistic LASSO regression model, some coefficients might be shrunk to zero due to the regularization parameter. This means that these predictors are not included in the model, and therefore, the logistic LASSO regression model performs variable selection [46]. Ridge regression is similar to LASSO regression except that the penalty term used in Ridge regression shrinks the coefficients of the predictors, but the coefficients will never be equal to zero. Hence, Ridge regression does not perform variable selection.

3. Estimation of predictor stability

For each protein, it was noted how many times each of the 100 logistic LASSO regression models included that protein as a predictor. These values were noted as a proportion and, therefore, will be referred to as the *proportion score*. The proteins with the highest proportion scores were taken as the most stable predictors, and the proteins with the lowest proportion scores were taken as the least stable predictors. To identify the protein signatures containing the most stable proteins, sets of proteins were generated according to the proportion scores. The sets were constructed in such a way that the first set contained proteins with a minimum proportion score of 0, thus containing all differentially expressed proteins. The remaining sets were constructed according to an incremental step of the proportion score of 0.05. For instance, the second set would contain proteins that had a minimum proportion score of 0.05 (included in at least 5% of the models) and the final set would contain proteins with a minimum proportion score of 1. For sets of proteins that were identical, only the sets with the corresponding highest proportion scores were selected, and therefore, some of the incremental steps were skipped.

4. **Performance in the discovery and replication cohort**

The discovery cohort was randomly split into two halves and used as a training set and a test set. For each protein signature, a Ridge regression model was fitted to the training set of the discovery cohort and tested in the corresponding test set using the R package *glmnet* with *alpha* = 0, and optimized with the function *cv.glmnet* using 10-fold cross validation in the training set. The model was then tested in the replication cohort. For each model fitted this way, another model was also fitted using the protein signature and the age of the patient.

General evaluation of model performance

The performance of each of the generated models was evaluated using Receiver Operating Characteristics (ROC) curves and the area under the ROC curve (AUC). For each ROC curve, Youden's index, also referred to as the best point, was used to identify the cut-off with the highest sensitivity and specificity.

The robustness of all evaluation parameters (AUC, sensitivity, specificity, and PPV) were investigated with bootstrapped 95% confidence intervals using 2000 stratified bootstrap replicates.

Survival analysis

Kaplan–Meier plots were generated for proteins included in the best-performing protein signature. The survival analyses were carried out for each of the targets for both the discovery cohort and the replication cohort. For each of the proteins, two groups were made, consisting of patients with expression of the given protein below the median expression of the protein and patients with expression above the median. These two groups were used to stratify the Kaplan–Meier plots into two curves, and a log-rank test was used to test whether the two curves were significantly

different. Furthermore, a Cox regression model was fitted to be able to calculate the hazard ratio (HR) and corresponding confidence interval (CI).

Subgroup analyses of patients divided by treatment

Patients were divided according to survival (≤180 days and >180 days) and according to treatment (gemcitabine, gemcitabine + nab-paclitaxel, or mFOLFIRINOX), creating six groups. The differential expression of the proteins + CA19-9 between the groups was tested using a *t*-test or Wilcoxon rank-sum test. The protein expressions in gemcitabine-treated patients surviving ≤180 days were compared to protein expressions in gemcitabine-treated patients surviving >180 days. The same was comparison was made within the gemcitabine + nab-paclitaxel group and within the mFOLFIRINOX group. The aim was to investigate whether protein expression at baseline in a particular treatment group could be prognostic. The comparisons were also performed for all patients combined (i.e., not divided by treatment) resulting in a total of four comparisons made. The median NPX value of each protein in a survival group was compared within a treatment group or in all patients combined (e.g., IL-8 in patients receiving gemcitabine and surviving ≤180 days compared with IL-8 in patients receiving gemcitabine and surviving >180 days).

The *p* values were adjusted for multiple testing using the Benjamini–Hochberg method. Boxplots were created for a visual presentation of the results.

Longitudinal analyses

For the longitudinal analyses, a linear mixed-effects model was fitted for each of the 93 markers (Olink Immuno-Oncology panel and CA19-9). The models were corrected for type of chemotherapy, age, and Eastern Cooperative Oncology Group (ECOG) performance status (PS). For each protein, we assessed whether there was a difference in protein abundance between the

group of patients that survived ≤1 year and the group of patients that survived >1 year over baseline and visit 3. This was achieved using an interaction term between the time-point variable and survival group variable.

The same assessment was performed for the comparison of the group of patients that survived ≤180 days and the group of patients that survived >180 days over baseline and visit 3, and over baseline and visit 2.

Paired boxplots were made to visualize the abundance levels of each protein over the time points. Furthermore, each plot was stratified according to the survival groups.

A table was generated to show all the significant interaction effects. A significant interaction term for a given protein suggests that there is a difference in the abundance level over the time points for the two groups of interest.

Each of the 139 patients with samples from all three time points had measurements for all 92 of the proteins for each time point. This group of patients was separated into two groups: patients surviving less than or equal to 180 days (≤180Days) and patients surviving longer than 180 days (>180Days). In order to investigate whether any of the 92 proteins + CA19-9 had similar changes in protein profiles over the three time points, a clustering analysis for the ≤180 days groups and a clustering analysis for the >180 days groups was carried out following the same steps. The function *kmeans* was used to cluster samples using the k-means clustering method from the package called *stats* from base R.

First, the mean for each protein for each time point was calculated. This gave three mean values for each protein, which will be referred to as a the protein profile. Second, a k-means algorithm was used to group the protein profiles into eight different clusters. Eight cluster groups were chosen because there are eight different combinations of patterns for three time points if we think of the

expression of proteins as either abundant or not abundant. Principal component analyses (PCA) were used to illustrate the cluster groups for both clustering analyses. The eight cluster groups from patients with OS ≤180 days were plotted alongside each other, as were the eight cluster groups from patients with OS >180 days.

Statistics and reproducibility

All statistical analyses were conducted in R version 3.6.1 (2019-07-05) [47]. The models were fitted and evaluated using functions from the R packages *glmnet* version 3.0-2 and *pROC* version 1.16.2. Plots were generated using the R package *ggplot2* version 3.3.0. Imputation was performed using the R package *impute* version 1.58.0. The R package *survminer* (version 0.4.6) was used to draw Kaplan–Meier plots. The functions *survdiff* and *coxph* from the R package *survival* (version 3.1-12) were used to perform the log-rank test and fit the Cox regression model, respectively. Linear mixed-effects models were fitted using the packages called *lme4* version 1.1-21 and *lmerTest* version 3.1-1. The function *kmeans* was used to cluster samples using the k-means clustering method from the package called *stats* from base R. The functions *fviz_nbclust* and *fviz_cluster* from the package called *factoextra (v. 1.0.5)* were used to visualize the expected number of clusters and the results of the K-means clustering algorithm using PCA.

Second statistical approach:

Data preparation

We aimed to develop a model to predict overall survival. The dataset comprised a single data table, the rows of which indexed the $N = 363$ serum samples, and with the columns indexing the $K = 92$ protein levels.

The protein levels, henceforth referred to as features, were log-transformed and standardized, according to the following formulas:

$$t_j = \log\left(1 + x_j\right) \text{ and } z_{i,j} = \frac{t_{i,j} - \mu(t_j)}{\sigma(t_j)}$$

Here, $x_j \in \{R\}_{i=1}^{N}$ is the *j*-th feature of the dataset (*j* = 1, 2, 3, … , *K*). The vector t_j is the log-transformed form of x_j, with elements $t_{i,j}$ (*i* indexes the samples: *i* = 1, 2, 3, … , *N*). The scalars $\mu(t_j)$ and $\sigma(t_j)$ are the mean and standard deviation of the *j*-th transformed feature, respectively.

The training and test set

From the 363 samples, 70% of the cases were randomly allocated to the discovery cohort (training, $N_{training}$ = 267), and 30% to the validation set (N_{test} = 106). Due to the low number of patients in the treatment-stratified subgroups, the models were also evaluated using the whole dataset.

The effect of the single protein levels on OS was evaluated using median separation. For exploring the prognostic capabilities of each of the proteins, Cox regression analyses and Kaplan–Meier plots were made, resulting in hazard ratios (HRs) and log-rank *p* values.

Cox Logistic regression

A non-negative L1 (LASSO)-regularized Cox Regression model was trained to predict overall survival. The L1, i.e., LASSO regulariser, was preferred to the other methods due to its feature-selection property.

The non-negative LASSO model was trained using the *glmnet* R package. The penalty parameter (lambda, λ) was trained using a 10-fold nested cross-validation strategy. In order to account for a robust solution in both lambda and the weights, this 10-fold cross-validation step was repeated 500

times. The final parameter λ was selected as the median of the resulting 500 λ values, and the final model weights were achieved using its value. The distribution of the resulting weights from the 500 iterations signifies the robustness of the final model. A risk score for each patient was calculated using the linear combination of levels of each protein multiplied by the corresponding regression coefficient.

Model evaluation

The performance of the model was evaluated in various ways. ROC curves were created using the *pROC* R package, and time-dependent ROC curves were determined using the *survivalROC* package at 3, 6, 12, 24 months. The effect of risk score on prognosis (overall survival) was evaluated using Kaplan–Meier plots, and log-rank *p* values were determined for the median cut-off and best cut-off for the risk score using the *maxstat* R package.

Longitudinal analyses

In this analysis, the three timepoints (at baseline, before the second treatment cycle, and at the time of the first CT scan) were not considered categorical variables; rather, the number of days from the baseline sample was used. This approach was used for the patients with all three timepoint samples available, and for the patients with two timepoint samples available (only baseline and before the second treatment cycle). Furthermore, the relationship between survival and the protein levels in the samples taken at the three mentioned timepoints was also evaluated using Cox regression and log-rank tests. Both univariate and multivariate analyses were performed for each

protein at each timepoint. The multivariate analyses included the clinical factors age, stage, baseline PS, baseline CA19-9, and type of palliative chemotherapy.

The results for the early changes in protein levels are visually presented in a simplified manor. In the analyses, the timepoints were not used as categorical variables, but rather, as continuous variables using the number of days between baseline and sample 2 and sample 3. Furthermore, the patients were not separated according to OS <6 months or >6 months in the analyses. However, when visually presenting these results (Supplementary Figure S10), the timepoints were used as categorical variables and patients were divided according to OS <6 months or >6 months, making it a simplified version of the results.

Supplementary File S2: Supplementary results

Prognostic protein panels for different survival durations

The results from the remaining three comparisons based on different OS cut-offs (≤90 days vs. >90 days; ≤180 days vs. >180 days; and <90 days vs. >1 year) and focusing on prognostic protein signatures can be found in Supplementary Tables S5 and S6. Several of the proteins included in Index I, such as CSF-1, IL-6, and TRAIL, were also primary proteins driving these prognostic models. The remaining proteins from Index I (PDCD1, TNFRSF12A, TWEAK, and CA19-9) were mainly part of the prognostic models when patients with longer OS (<90 days vs. OS >1 year and <90 days vs. OS >2 years) were considered.

Prognostic subgroup analyses

For the patients in the different treatment groups, the most significantly different levels of plasma proteins between the patients according to survival (≤180 days vs. >180 days) were CCL20, HGF, IL-6, and IL-8 for the gemcitabine treated group ($p < 0.001$ for all the mentioned proteins); CAIX, CSF-1, and MCP-3 for the gemcitabine + nab-paclitaxel treated group ($p < 0.001$ for all the mentioned proteins); and ANGPT2, CCL23, IL-6, IL-10, TNFRSF12A, and TNFSF14 for the mFOLFIRINOX group ($p < 0.05$ for all the mentioned proteins).

In the second statistical approach, six plasma proteins were found to be statistically significant in all four comparisons: ANGPT2, CSF-1, HGF, IL-6, MCP-3, and TNFRSF12A (Supplementary Table S7). For the patients in the different treatment groups, the most significantly different levels of plasma proteins between the patients according to survival (≤180 days vs. >180 days) in the second

statistical approach were caspase 8 (CASP-8), HGF, IL-6, IL-8, and MCP-3 for the gemcitabine treated group ($p < 0.001$ for all the mentioned proteins); CSF-1, IL-8, and MCP-3 for the gemcitabine + nab-paclitaxel treated group ($p < 0.001$ for all the mentioned proteins); and ANGPT2, CSF-1, IL-6, and angiopoietin-1 receptor (TIE2) for the mFOLFIRINOX group ($P < 0.001$ for all the mentioned proteins).

Early changes in circulating-protein levels after start of palliative chemotherapy and survival

With the first statistical approach, the changes in the protein profiles were grouped into eight different clusters (Supplementary Figure S6). The clustering did not reveal any clear changes in profiles separating one from the other (Supplementary Figure S7), meaning that there was not one group of proteins that could be used over time to monitor patients undergoing palliative chemotherapy.

However, when the patients were split according to survival (≤/> 180 days and ≤/> 1 year), the interaction analyses revealed several proteins with an interaction effect yielding p values of <0.05, suggesting that a difference in the abundance level over the time points had a relation to survival. Two proteins (fractalkine (CX3CL1) and IL-33) had significant interaction effects with regard to changes in NPX values from baseline to before the second treatment cycle samples, and from baseline to the time of the first CT evaluation samples in relation to survival ≤/>180 days. This suggests a relation between the change in NPX values of the two proteins and the survival of the patients (≤/>180 days). Four proteins (TNFRSF12A, decorin (DCN), adenosine deaminase (ADA), and matrix metalloproteinase-7 (MMP7)) had significant interaction effects regarding changes in NPX values from baseline to the first CT evaluation samples in relation to both survival ≤/>180

days and survival ≤/> 1 year. For details on proteins with p values <0.05 in the interaction analyses, see Supplementary Table S8.

Paired boxplots were made to visualize the abundance levels of each protein over the time points, and each plot was stratified according to the survival groups. For plots of the proteins with interaction terms with $p < 0.01$, see Supplementary Figure S8.

In the second statistical approach, univariate analyses identified 33 proteins with significant log-rank tests, and 23 of these were also significant in the multivariate analyses of the blood samples taken before the first CT scan (at approximately 3 months after chemotherapy initiation) (Supplementary table S9C). Of the proteins significantly associated with survival in the multivariate analyses, one protein (CSF-1) was also in prognostic Index I, one protein (CXCL13) was in Index II, and two proteins (IL-6 and TNFRSF12A) were in both Index I and Index II. The results from the analyses of the baseline samples and the samples before the second treatment cycle are shown in Supplementary Table S9. Simplified plots of the proteins with longitudinal changes that were statistically significantly associated with OS ($p < 0.001$) are found in Supplementary Figure S9.

IL-6 ELISA

The IL-6 ELISA data were compared with the IL-6 data from the Olink analyses with Spearman's correlation. ELISA IL-6 showed a high correlation with Olink IL-6, with a Spearman's correlation coefficient of 0.9211 (see Supplementary Figure S10).

Supplementary Figures S1–S10

Supplementary Figure S1: Kaplan-Meier plots for the proteins included in Index I

The patients are divided according to protein expression (NPX values) below or above the median NPX of that given protein for each protein in the two indices.

a)

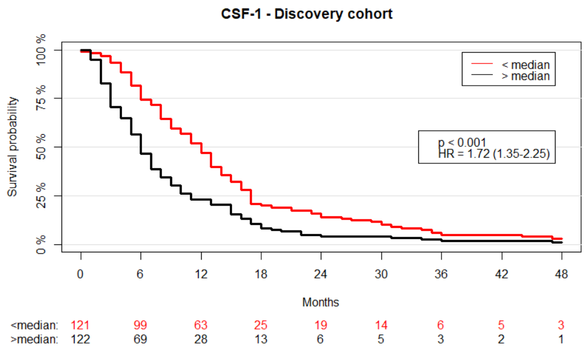

b)

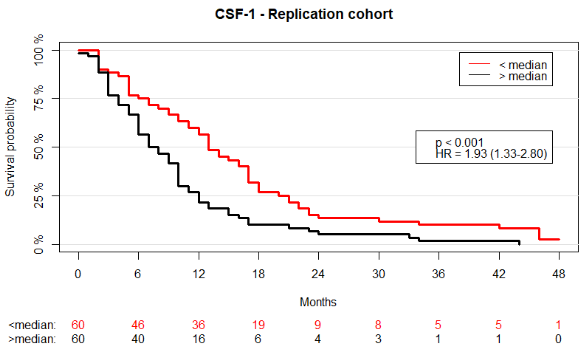

c)

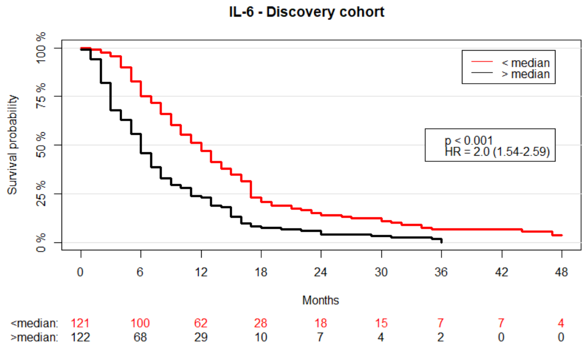

d)

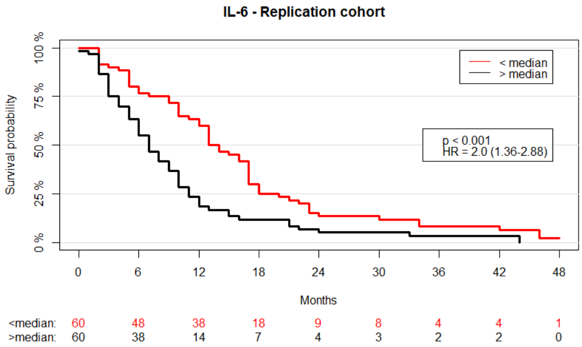

e)

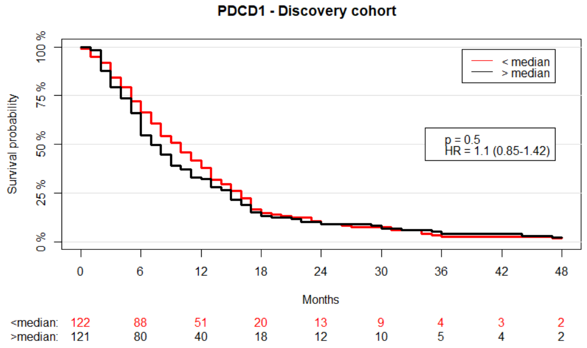

f)

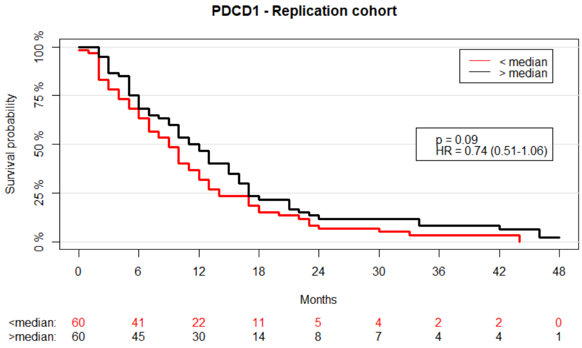

g) h)

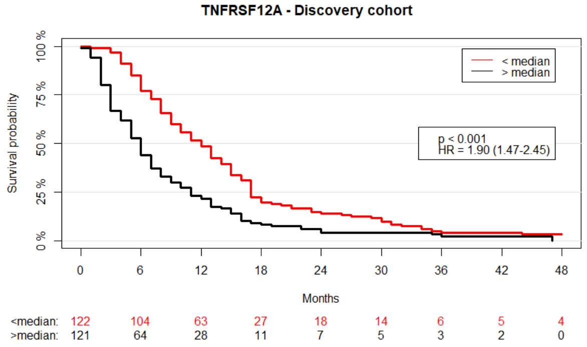
TNFRSF12A - Discovery cohort
Survival probability
Months
< median
> median
p < 0.001
HR = 1.90 (1.47-2.45)
<median: 122 104 63 27 18 14 6 5 4
>median: 121 64 28 11 7 5 3 2 0

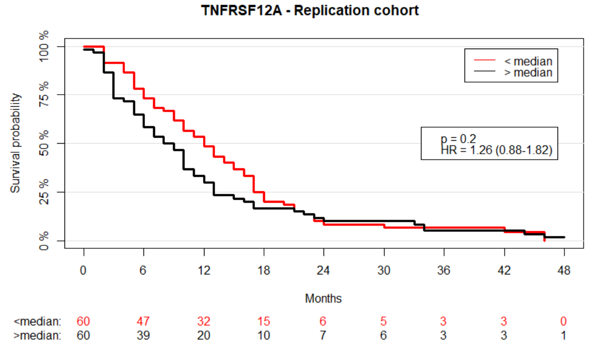
TNFRSF12A - Replication cohort
Survival probability
Months
< median
> median
p = 0.2
HR = 1.26 (0.88-1.82)
<median: 60 47 32 15 6 5 3 3 0
>median: 60 39 20 10 7 6 3 3 1

i) j)

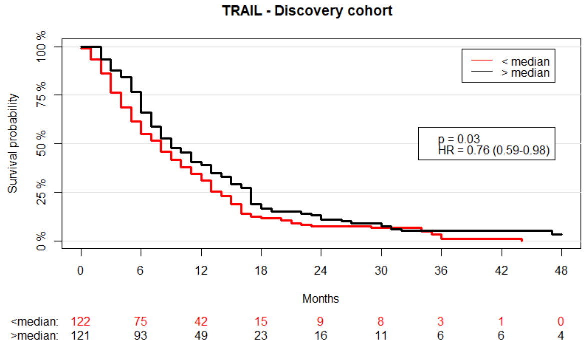
TRAIL - Discovery cohort
Survival probability
Months
< median
> median
p = 0.03
HR = 0.76 (0.59-0.98)
<median: 122 75 42 15 9 8 3 1 0
>median: 121 93 49 23 16 11 6 6 4

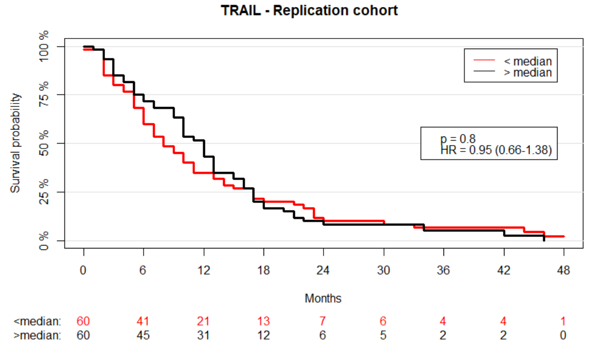
TRAIL - Replication cohort
Survival probability
Months
< median
> median
p = 0.8
HR = 0.95 (0.66-1.38)
<median: 60 41 21 13 7 6 4 4 1
>median: 60 45 31 12 6 5 2 2 0

k) l)

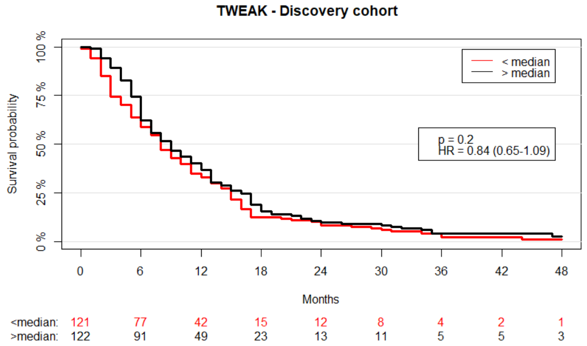
TWEAK - Discovery cohort
Survival probability
Months
< median
> median
p = 0.2
HR = 0.84 (0.65-1.09)
<median: 121 77 42 15 12 8 4 2 1
>median: 122 91 49 23 13 11 5 5 3

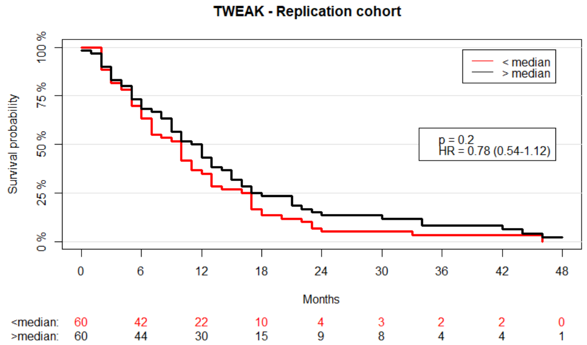
TWEAK - Replication cohort
Survival probability
Months
< median
> median
p = 0.2
HR = 0.78 (0.54-1.12)
<median: 60 42 22 10 4 3 2 2 0
>median: 60 44 30 15 9 8 4 4 1

m) n)

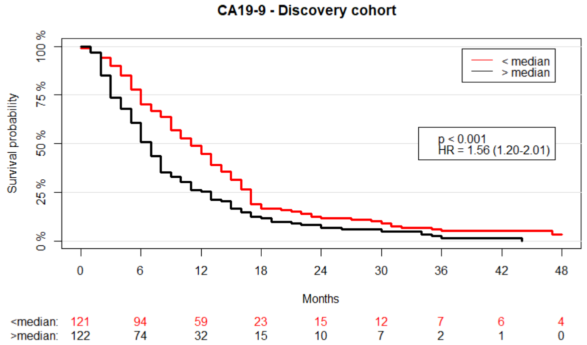
CA19-9 - Discovery cohort
Survival probability
Months
< median
> median
p < 0.001
HR = 1.56 (1.20-2.01)
<median: 121 94 59 23 15 12 7 6 4
>median: 122 74 32 15 10 7 2 1 0

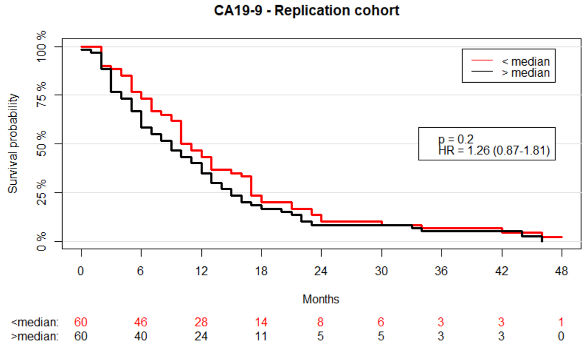
CA19-9 - Replication cohort
Survival probability
Months
< median
> median
p = 0.2
HR = 1.26 (0.87-1.81)
<median: 60 46 28 14 8 6 3 3 1
>median: 60 40 24 11 5 5 3 3 0

Supplementary Figure S2: Kaplan-Meier plots for the proteins included in Index II

The patients are divided according to protein expression (NPX values) below or above the median NPX of that given protein for each protein in the two indices.

a) b)

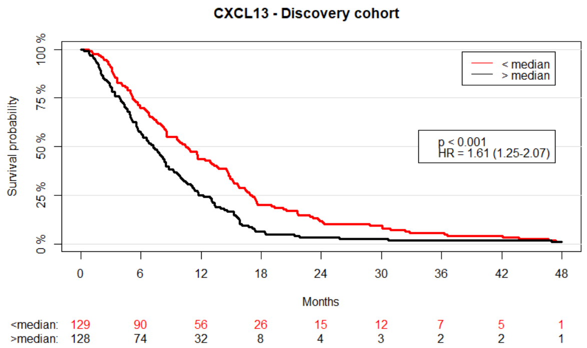

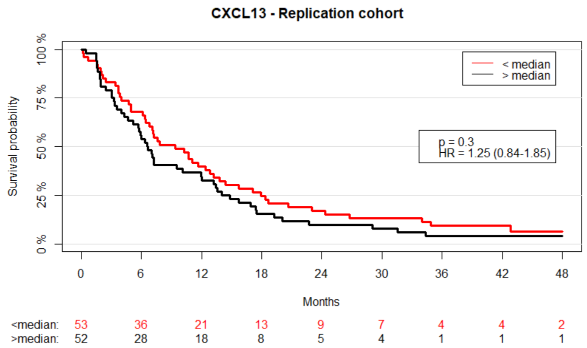

c) d)

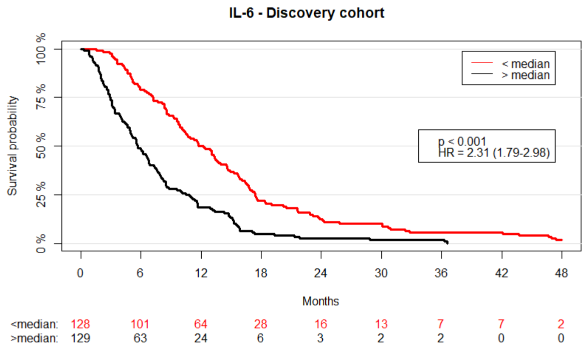

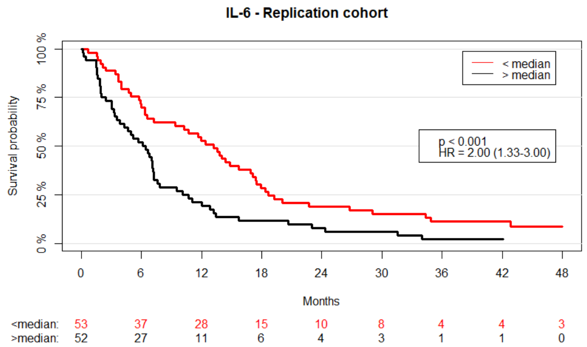

e) f)

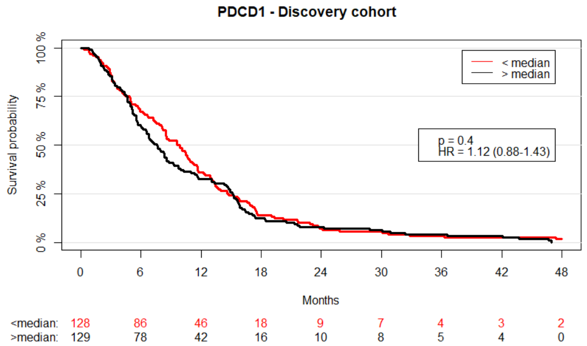

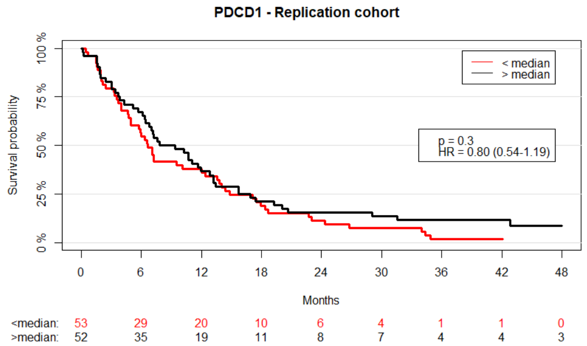

g) h)

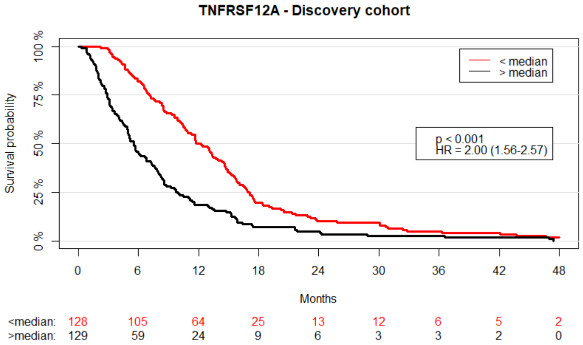
TNFRSF12A - Discovery cohort
Survival probability
100 %
75 %
50 %
25 %
0 %
< median
> median
p < 0.001
HR = 2.00 (1.56-2.57)
0 6 12 18 24 30 36 42 48
Months
<median: 128 105 64 25 13 12 6 5 2
>median: 129 59 24 9 6 3 3 2 0

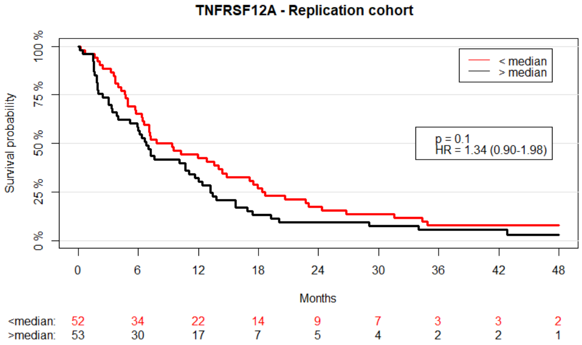
TNFRSF12A - Replication cohort
Survival probability
100 %
75 %
50 %
25 %
0 %
< median
> median
p = 0.1
HR = 1.34 (0.90-1.98)
0 6 12 18 24 30 36 42 48
Months
<median: 52 34 22 14 9 7 3 3 2
>median: 53 30 17 7 5 4 2 2 1

Supplementary Figure S3: Kaplan-Meier plot of patients divided by treatment group

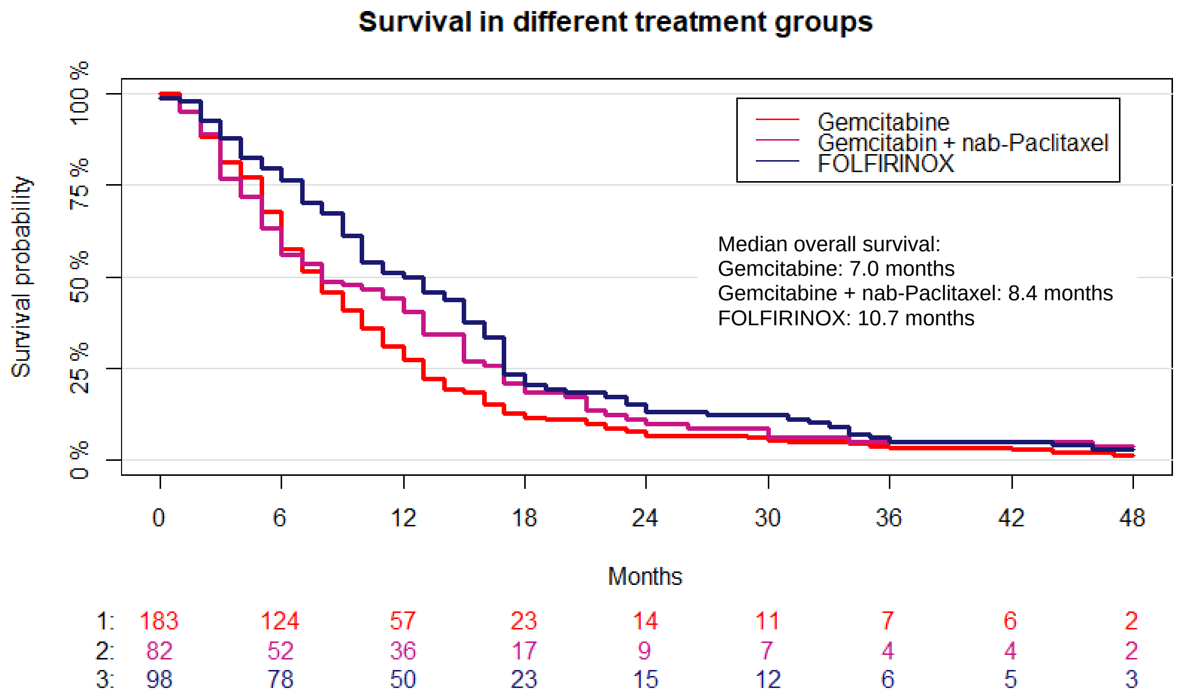

Supplementary Figure S4: Boxplots of the proteins with statistically significant differences in groups with survival ≤180 days (survival group A) and >180 days (survival group B) in all four comparisons for the predictive protein panel

a) ANGPT2 b) IL-6

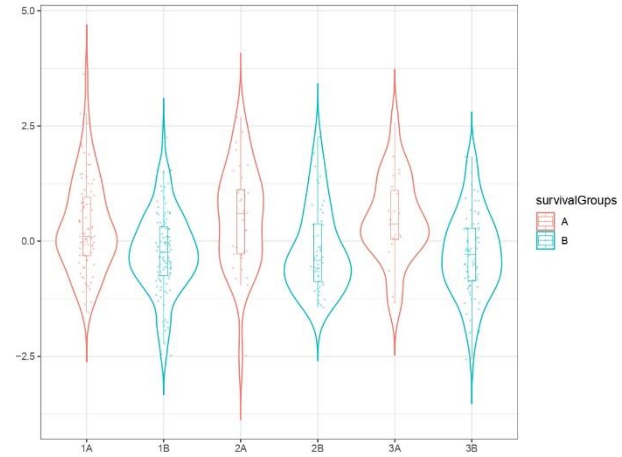

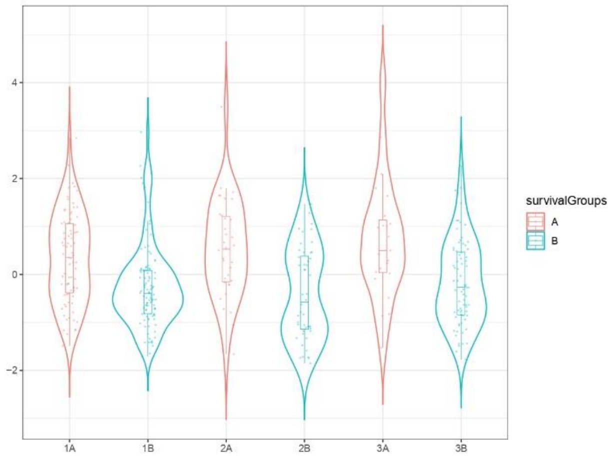

c) IL-10 d) TNFRSF12A

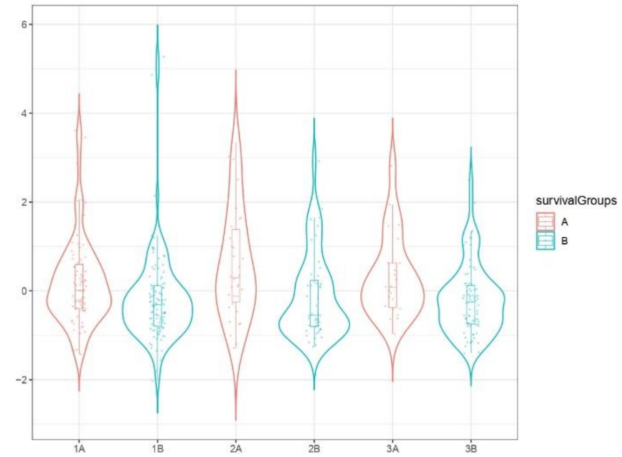

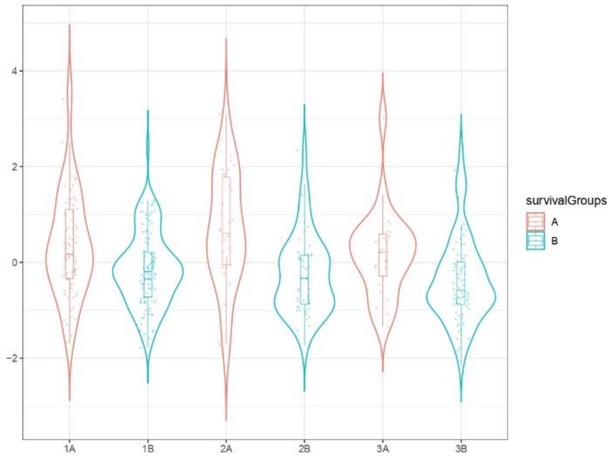

Treatment group 1 = Gemcitabine, Treatment group 2 = Gemcitabine + nab-Paclitaxel, Treatment group 3 = mFOLFIRINOX, Survival group A: ≤180 days, Survival group B: >180 days

Supplementary Figure S5: Subgroup analyses of survival in patients divided by treatment.

Showing proteins that were significantly different between patients with an OS ≤180 days compared with patients with an OS of >180 days, looking at the patients in the different subgroups. a) Overlapping proteins found in the first statistical approach. b) Overlapping proteins found in the second statistical approach. Blue circles = gemcitabine, red circles = gemcitabine + nab-paclitaxel, green circles = mFOLFIRINOX.

a)

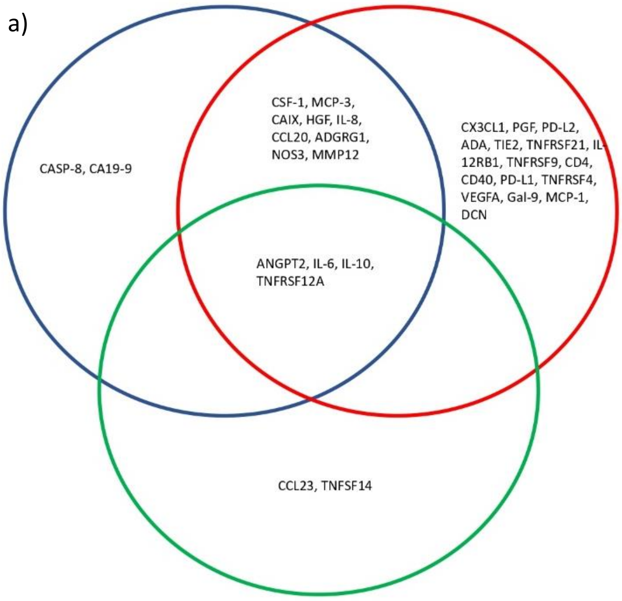

b)

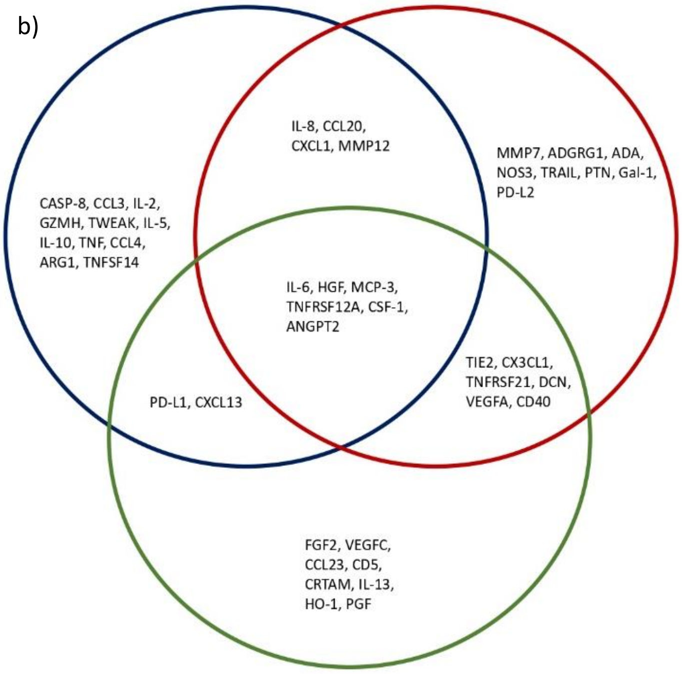

Supplementary Figure S6: Cluster plots from the longitudinal analyses

a) Cluster plot for the patients with OS ≤180 days

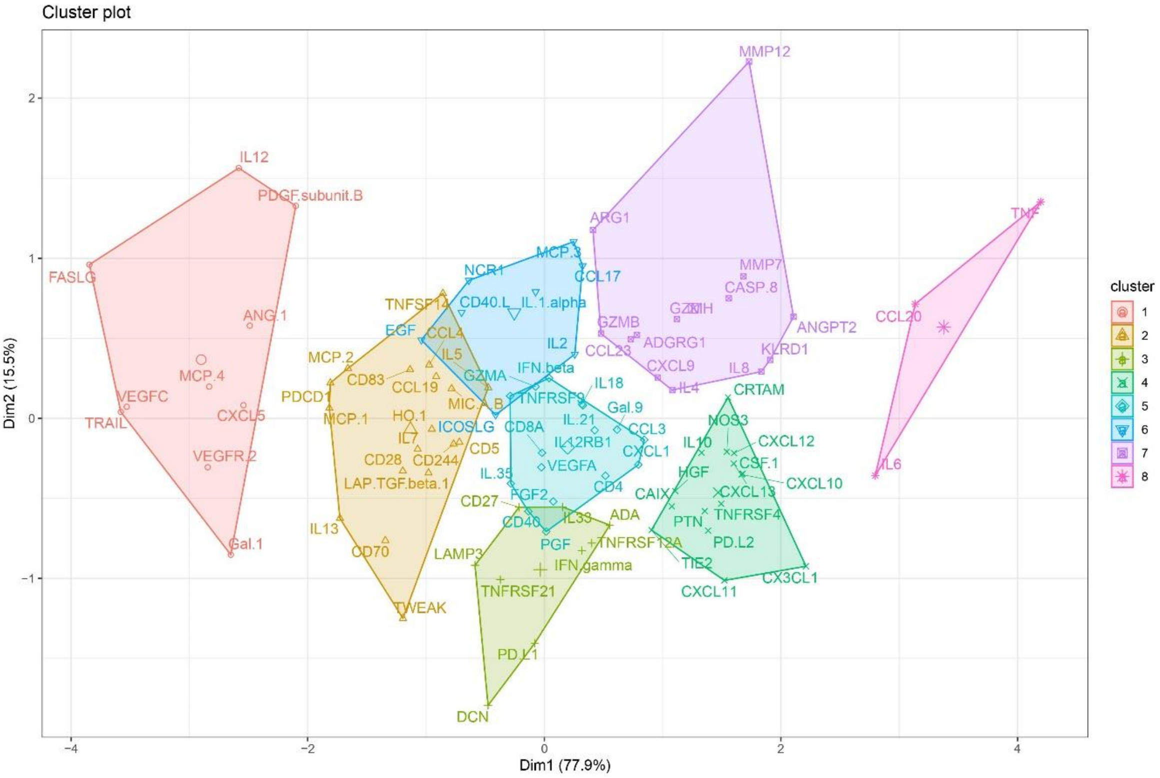

b) Cluster plot for the patients with OS >180 days

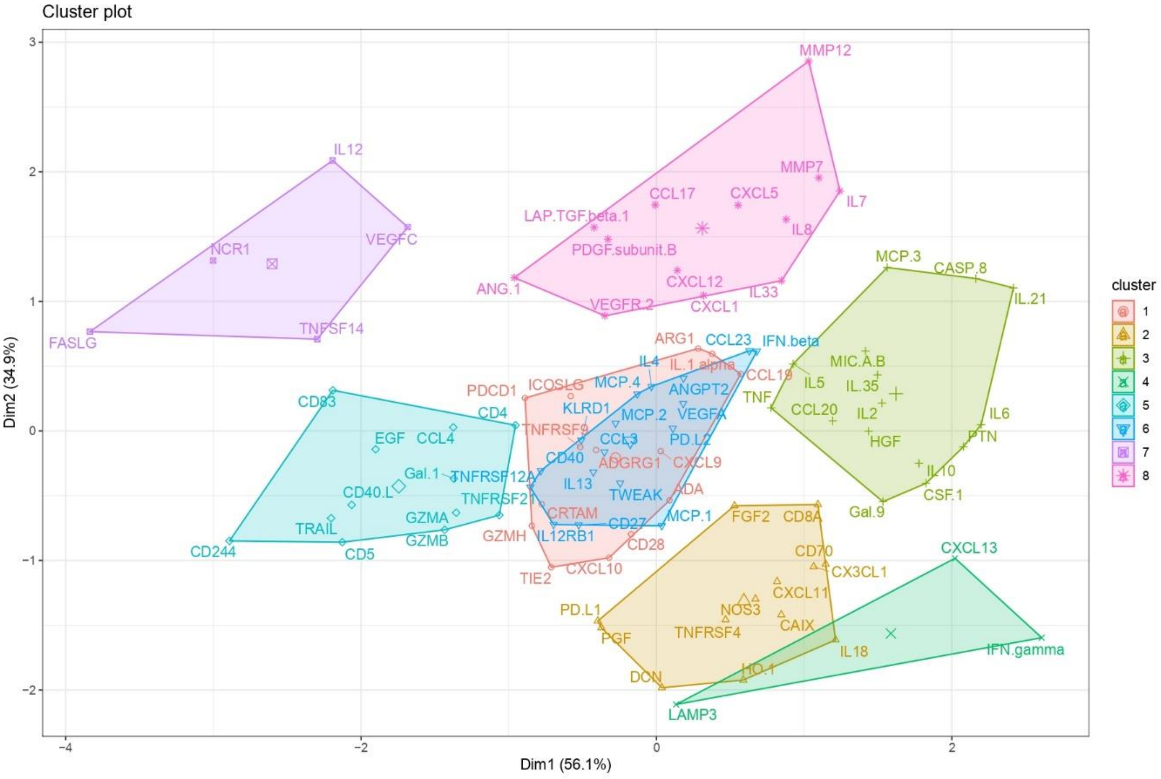

Supplementary Figure S7: Protein profile plots for the 139 patients with three longitudinal samples available

a) Protein profile plots for patients surviving ≤180 days

1) Cluster 1

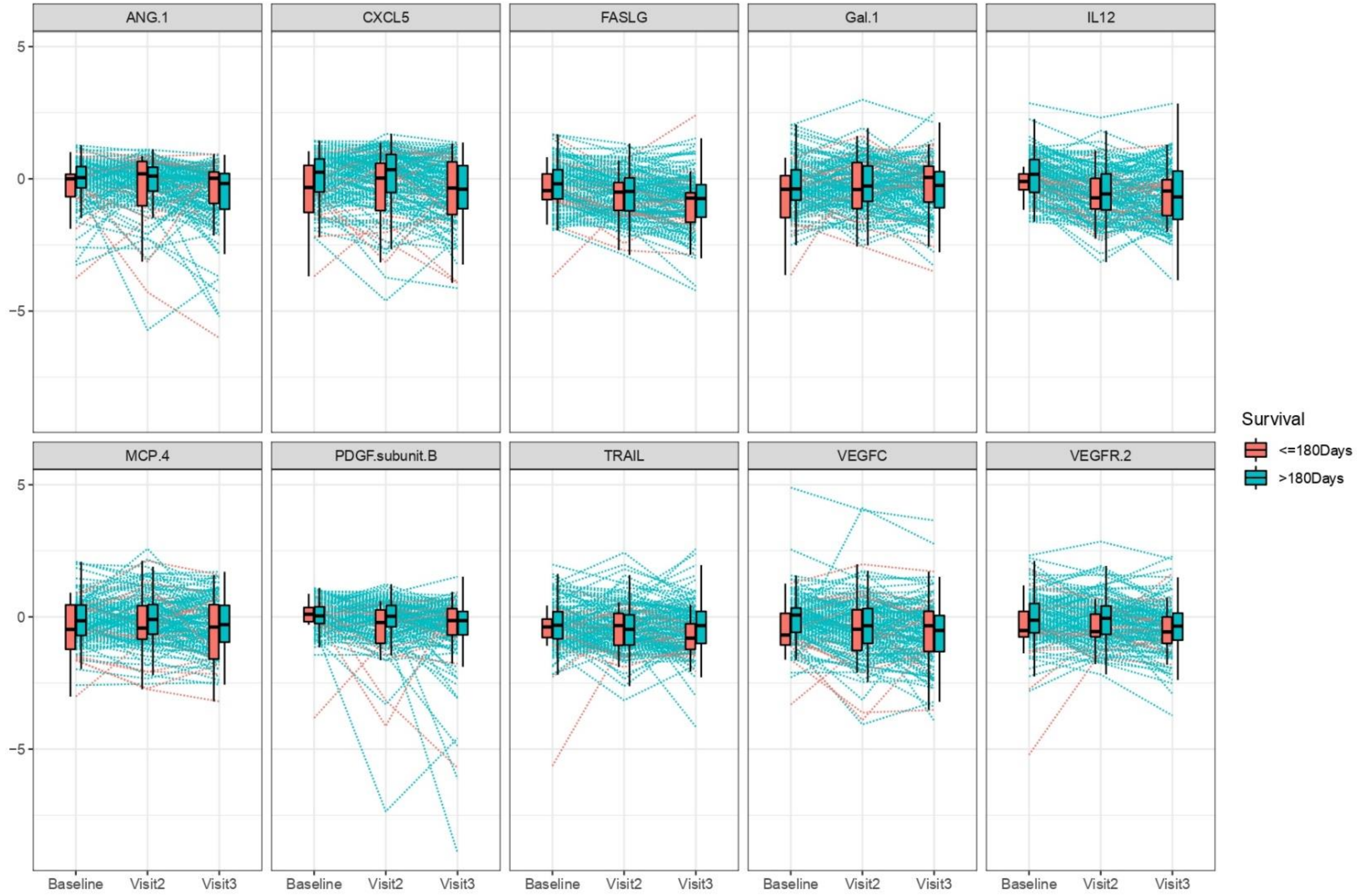

2) Cluster 2

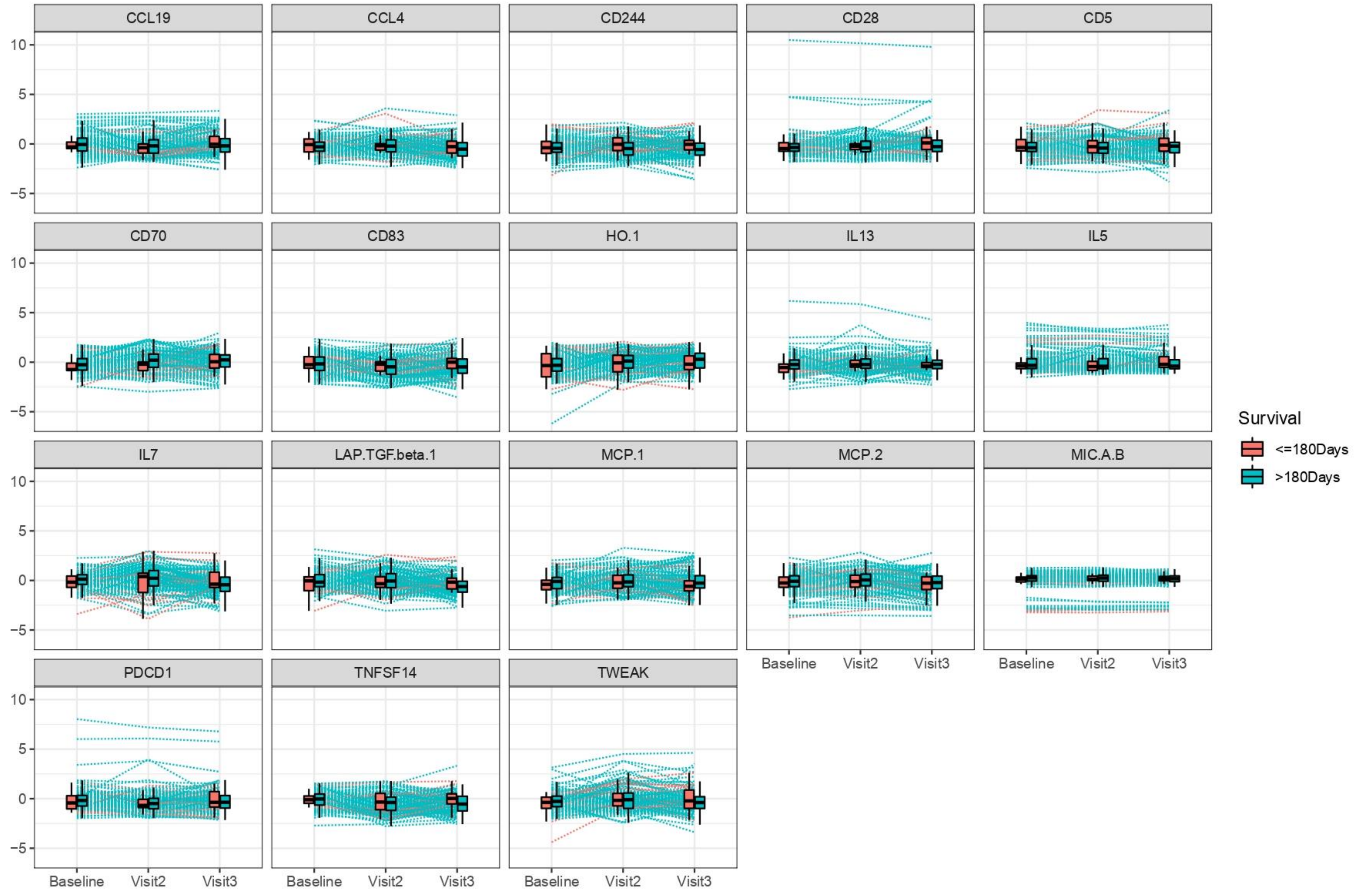

CCL19
CCL4
CD244
CD28
CD5
CD70
CD83
HO.1
IL13
IL5
IL7
LAP.TGF.beta.1
MCP.1
MCP.2
MIC.A.B
PDCD1
TNFSF14
TWEAK
10
5
0
−5
Baseline
Visit2
Visit3
Survival
<=180Days
>180Days

3) Cluster 3

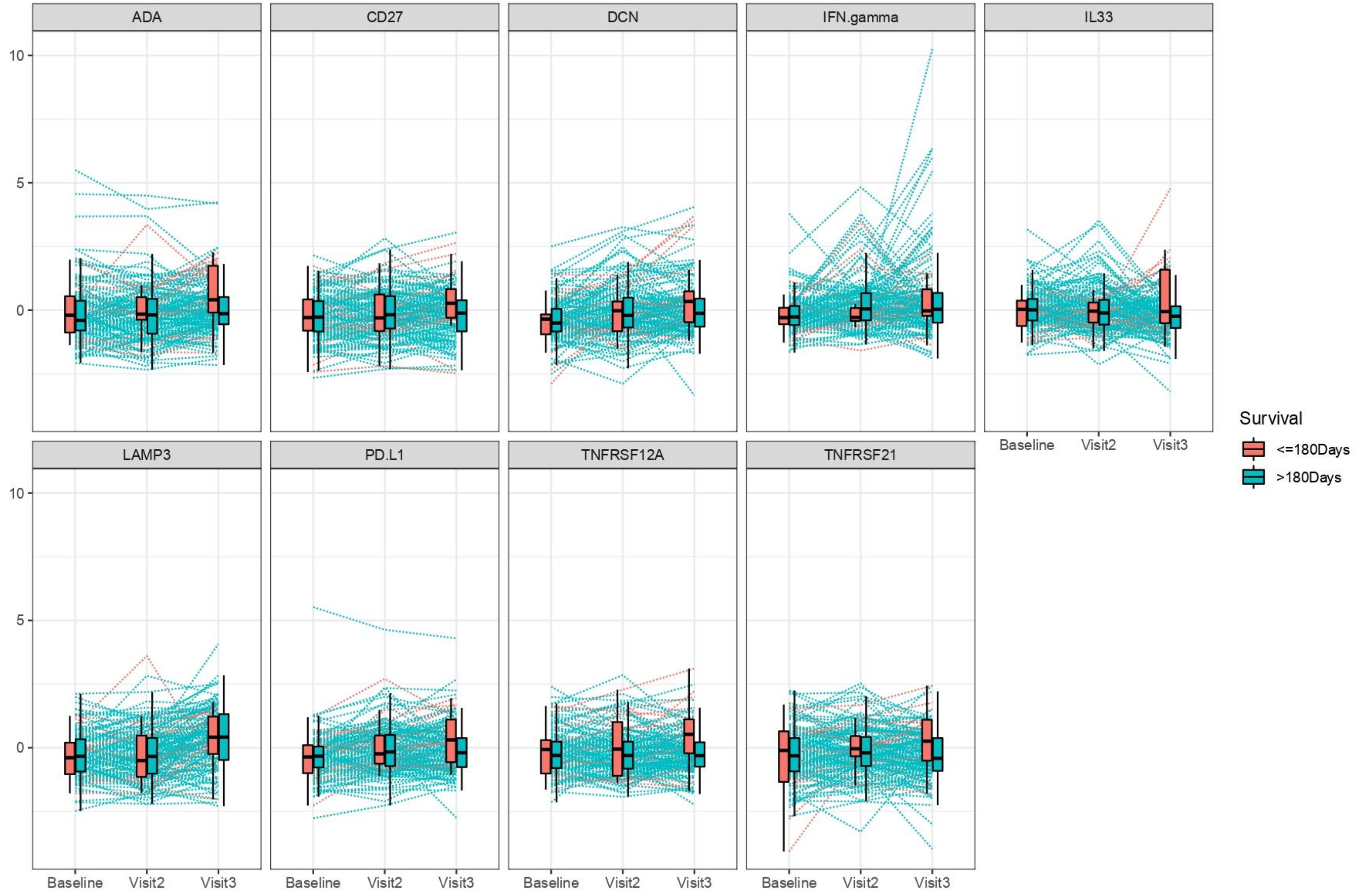

ADA
CD27
DCN
IFN.gamma
IL33
LAMP3
PD.L1
TNFRSF12A
TNFRSF21
10
5
0
Baseline
Visit2
Visit3
Survival
<=180Days
>180Days

4) Cluster 4

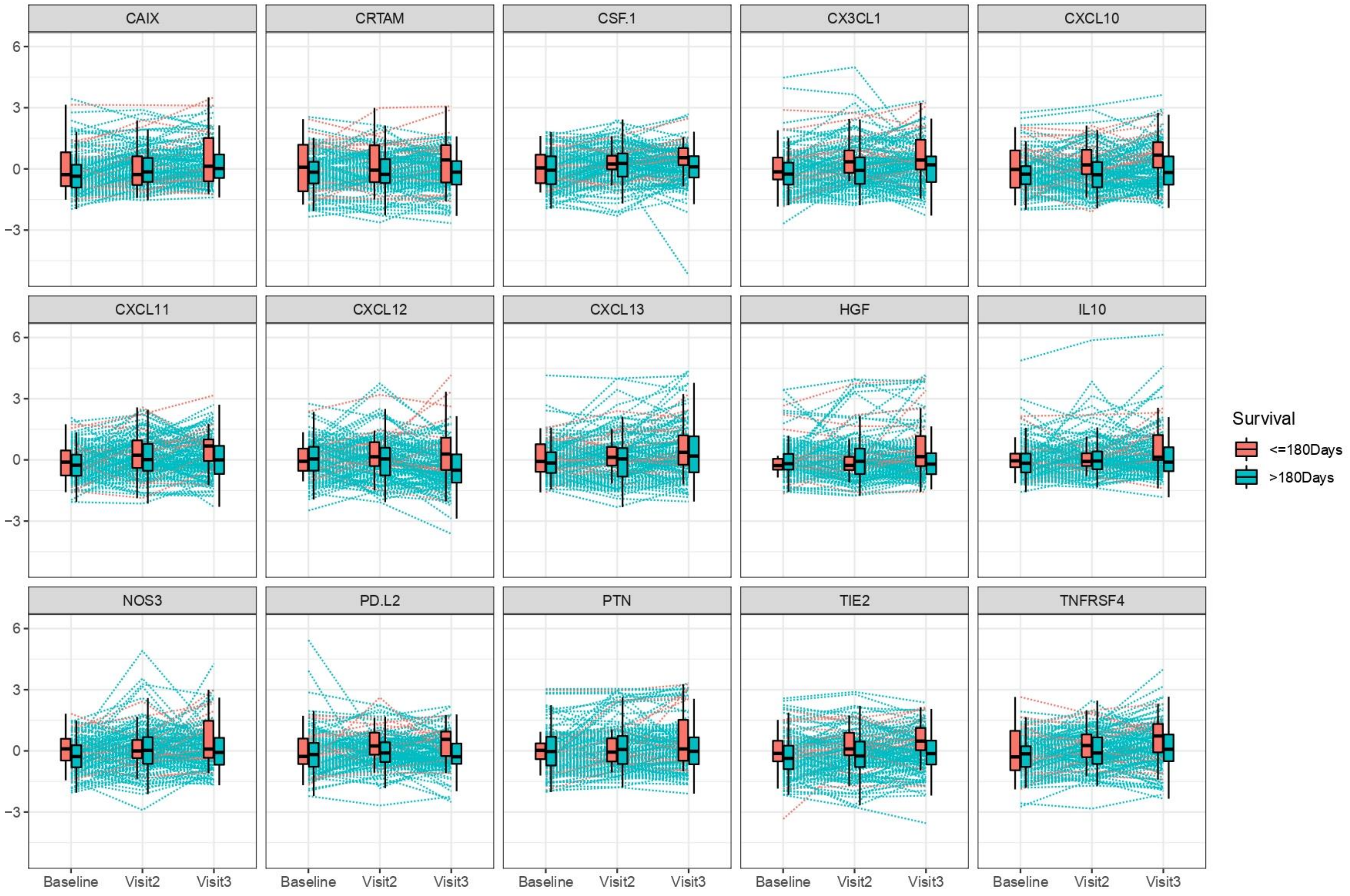

CAIX
CRTAM
CSF.1
CX3CL1
CXCL10
CXCL11
CXCL12
CXCL13
HGF
IL10
NOS3
PD.L2
PTN
TIE2
TNFRSF4
6
3
0
−3
Baseline
Visit2
Visit3
Survival
<=180Days
>180Days

5) Cluster 5

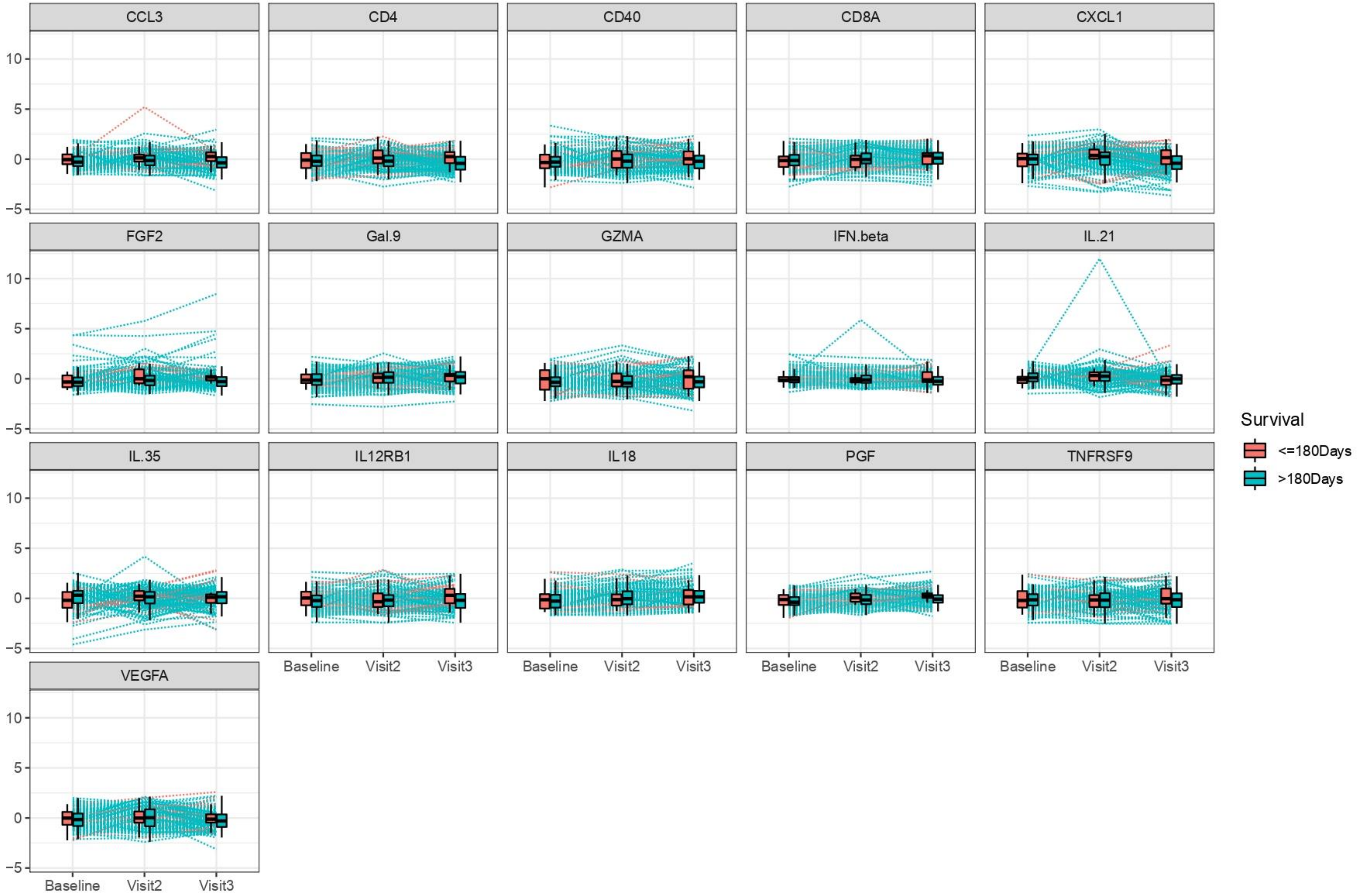

CCL3
CD4
CD40
CD8A
CXCL1
FGF2
Gal.9
GZMA
IFN.beta
IL.21
IL.35
IL12RB1
IL18
PGF
TNFRSF9
VEGFA
10
5
0
−5
Baseline
Visit2
Visit3
Survival
<=180Days
>180Days

6) Cluster 6

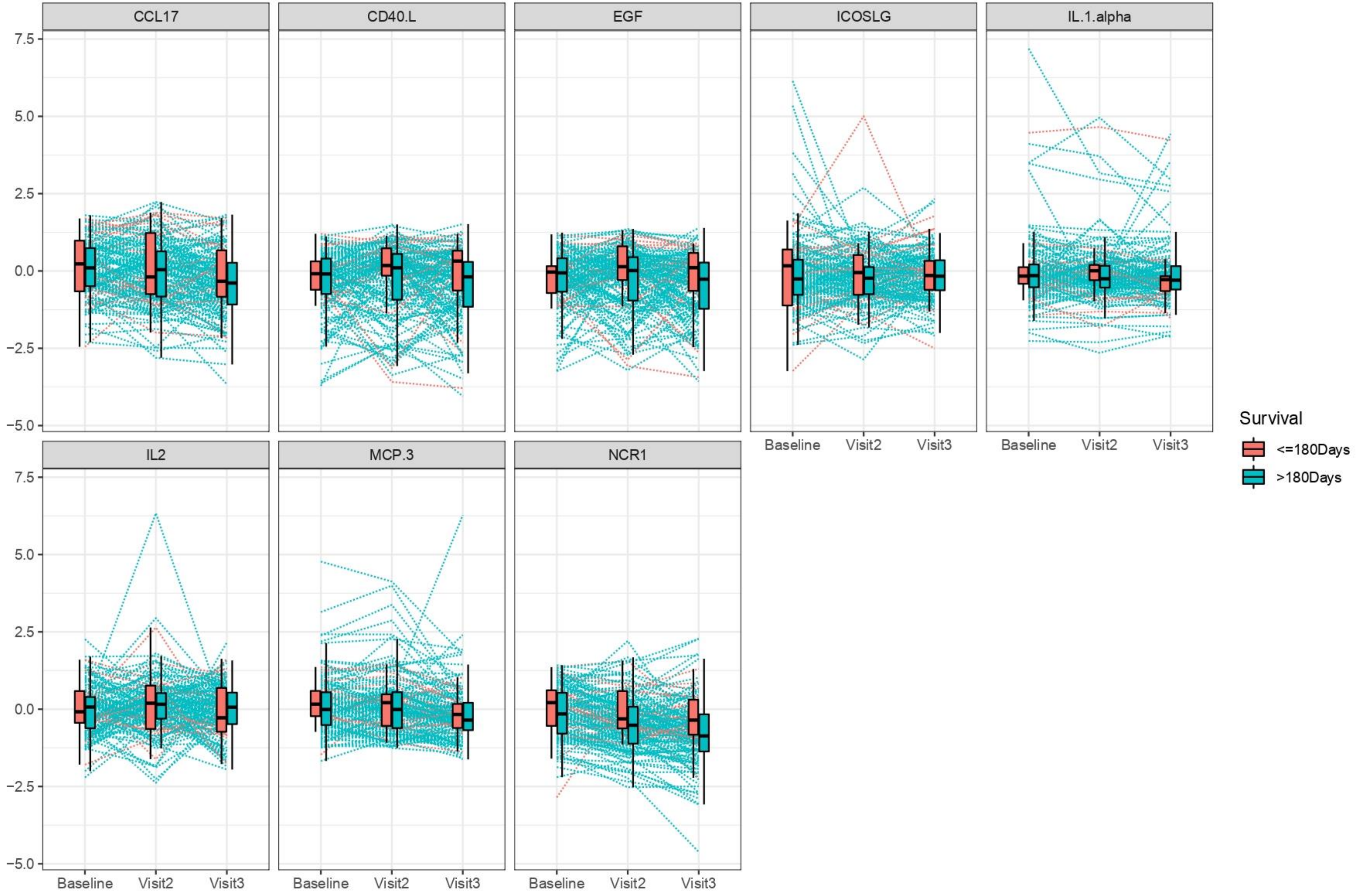

CCL17
CD40.L
EGF
ICOSLG
IL.1.alpha
IL2
MCP.3
NCR1
7.5
5.0
2.5
0.0
−2.5
−5.0
Baseline
Visit2
Visit3
Survival
<=180Days
>180Days

7) Cluster 7

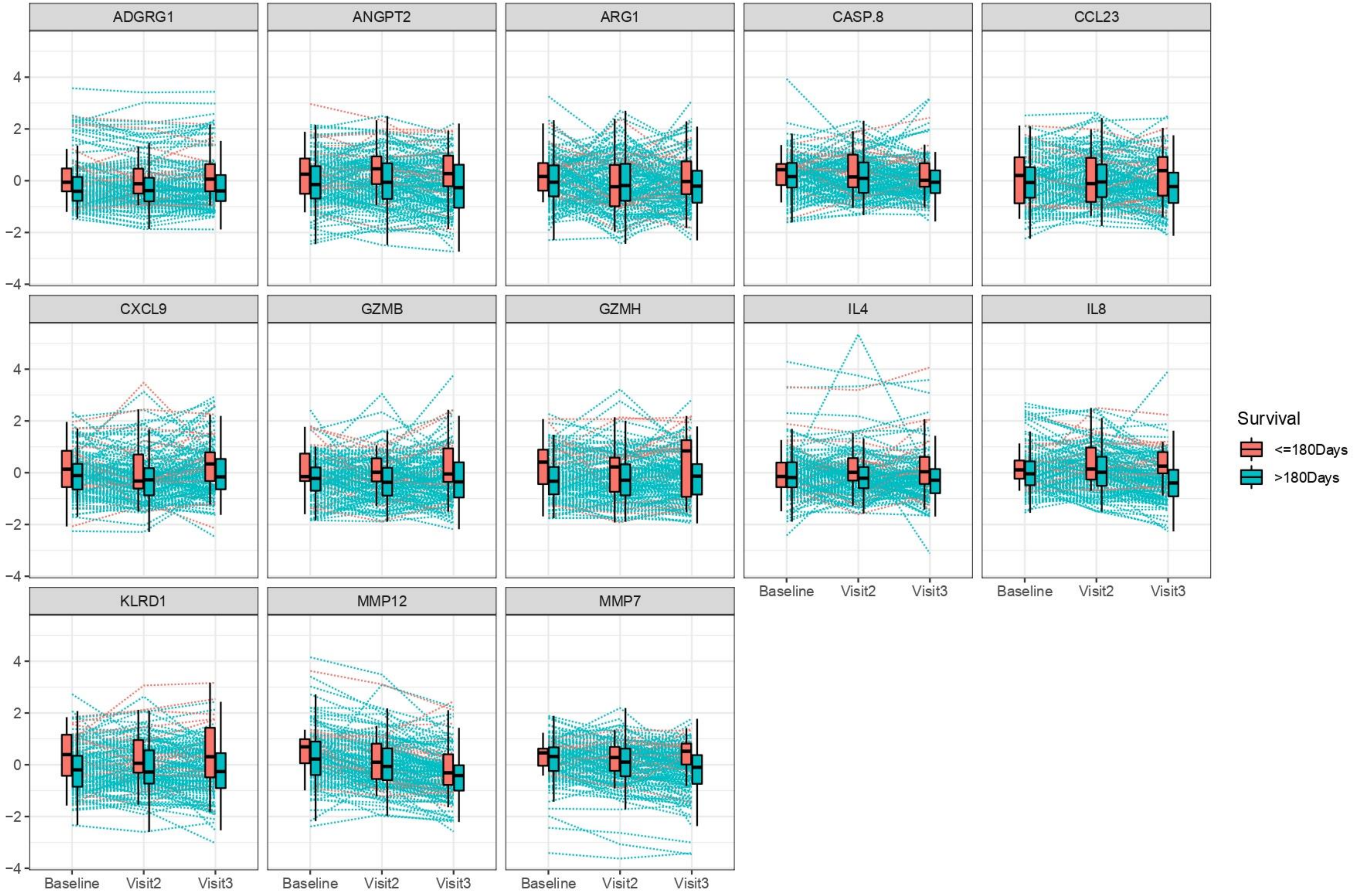

ADGRG1
ANGPT2
ARG1
CASP.8
CCL23
CXCL9
GZMB
GZMH
IL4
IL8
KLRD1
MMP12
MMP7
4
2
0
−2
−4
Baseline
Visit2
Visit3
Survival
<=180Days
>180Days

8) Cluster 8

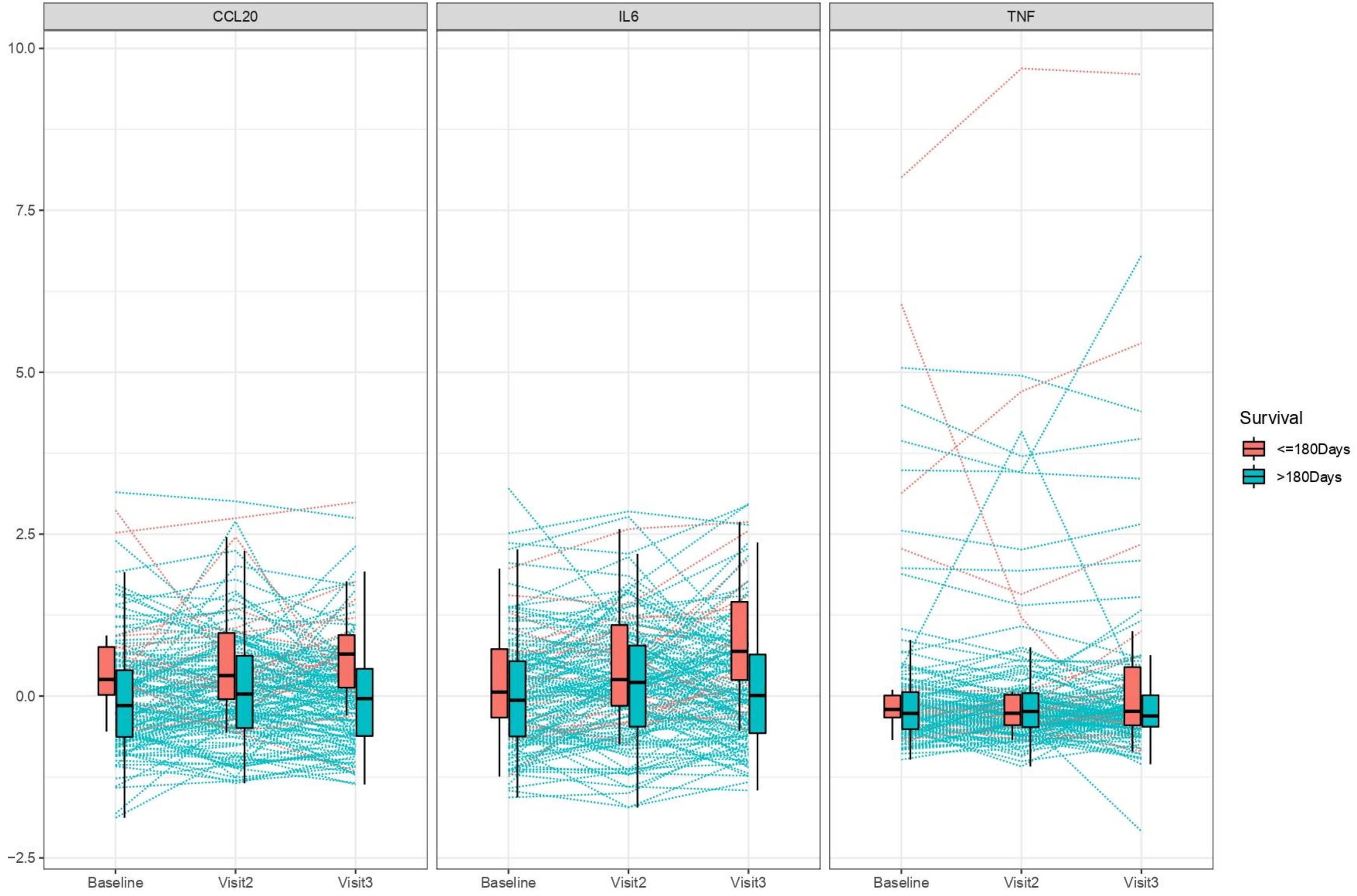
CCL20
IL6
TNF
10.0
7.5
5.0
2.5
0.0
−2.5
Baseline
Visit2
Visit3
Survival
<=180Days
>180Days

b) Protein profile plots for patients surviving >180 days

1) Cluster 1

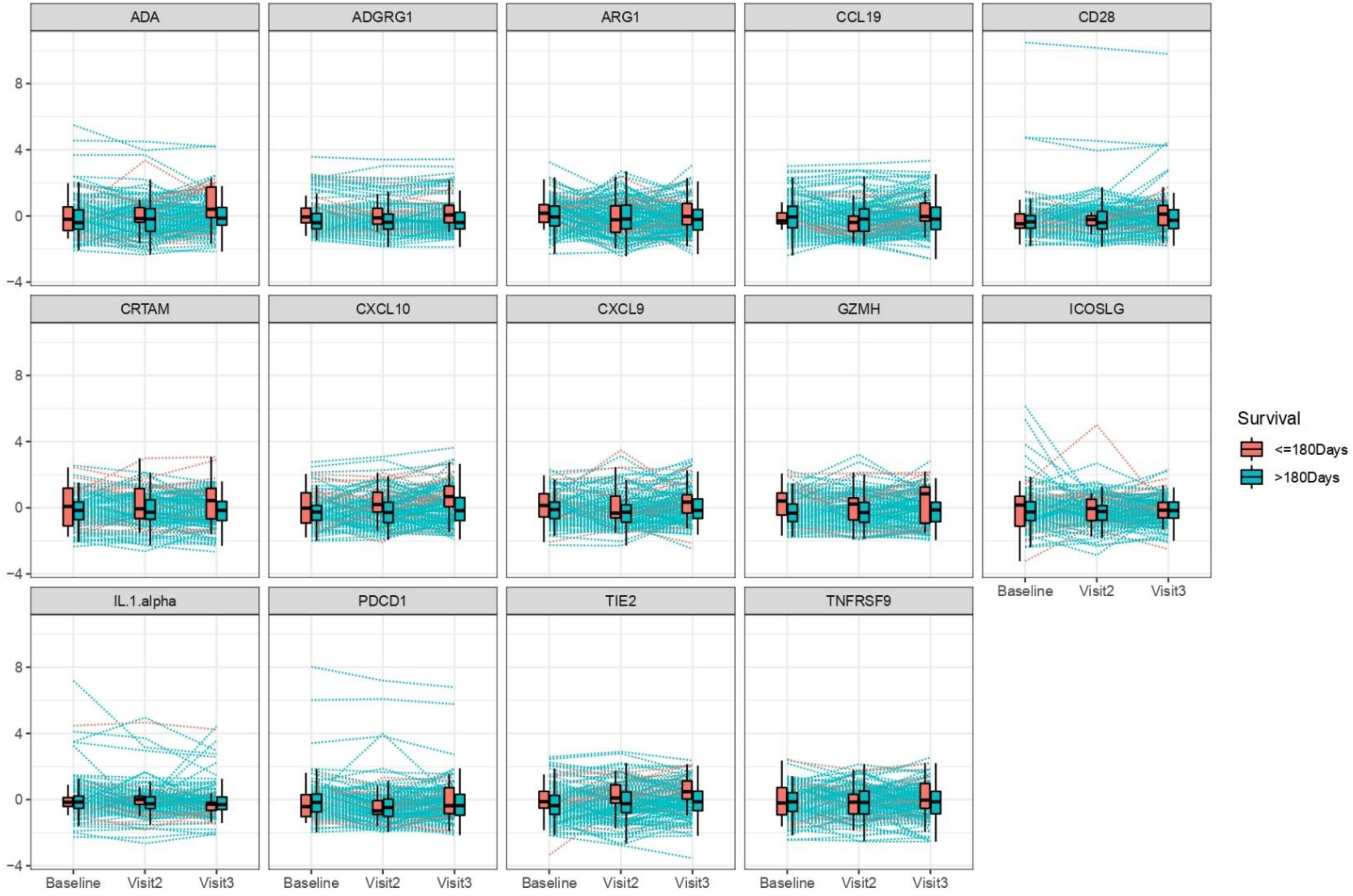

2) Cluster 2

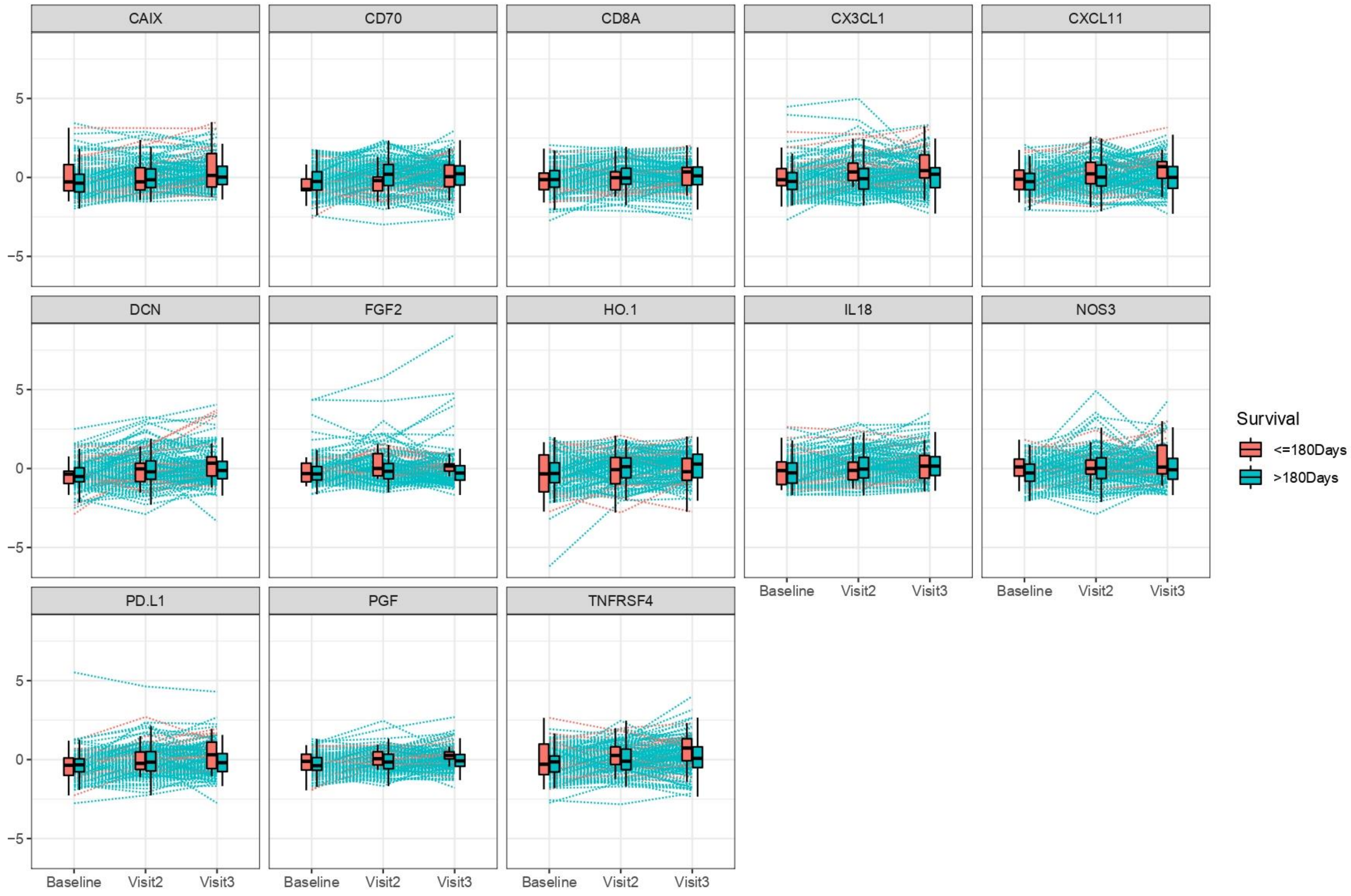
CAIX
CD70
CD8A
CX3CL1
CXCL11
DCN
FGF2
HO.1
IL18
NOS3
PD.L1
PGF
TNFRSF4
5
0
−5
Baseline
Visit2
Visit3
Survival
<=180Days
>180Days

3) Cluster 3

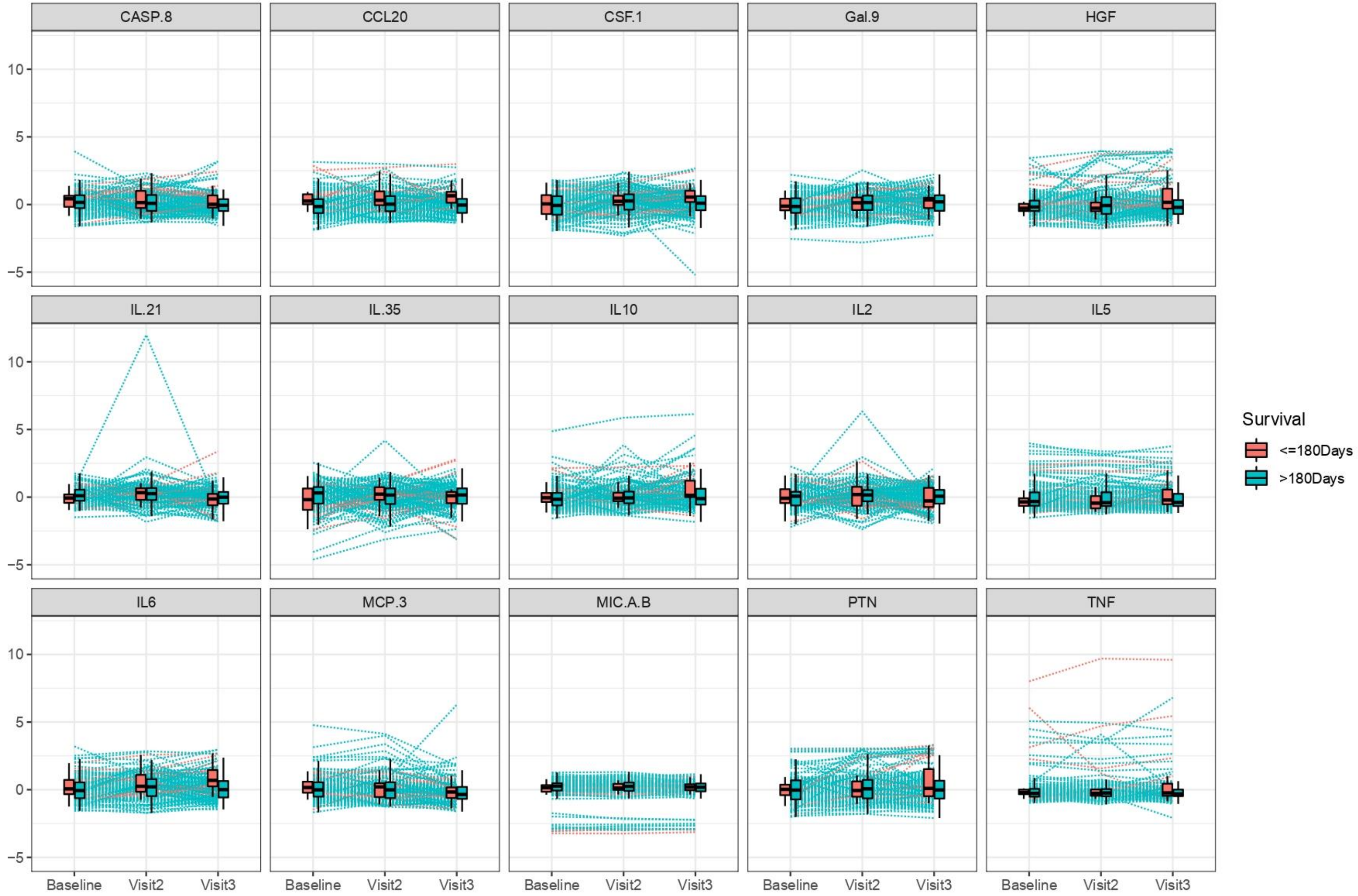

4) Cluster 4

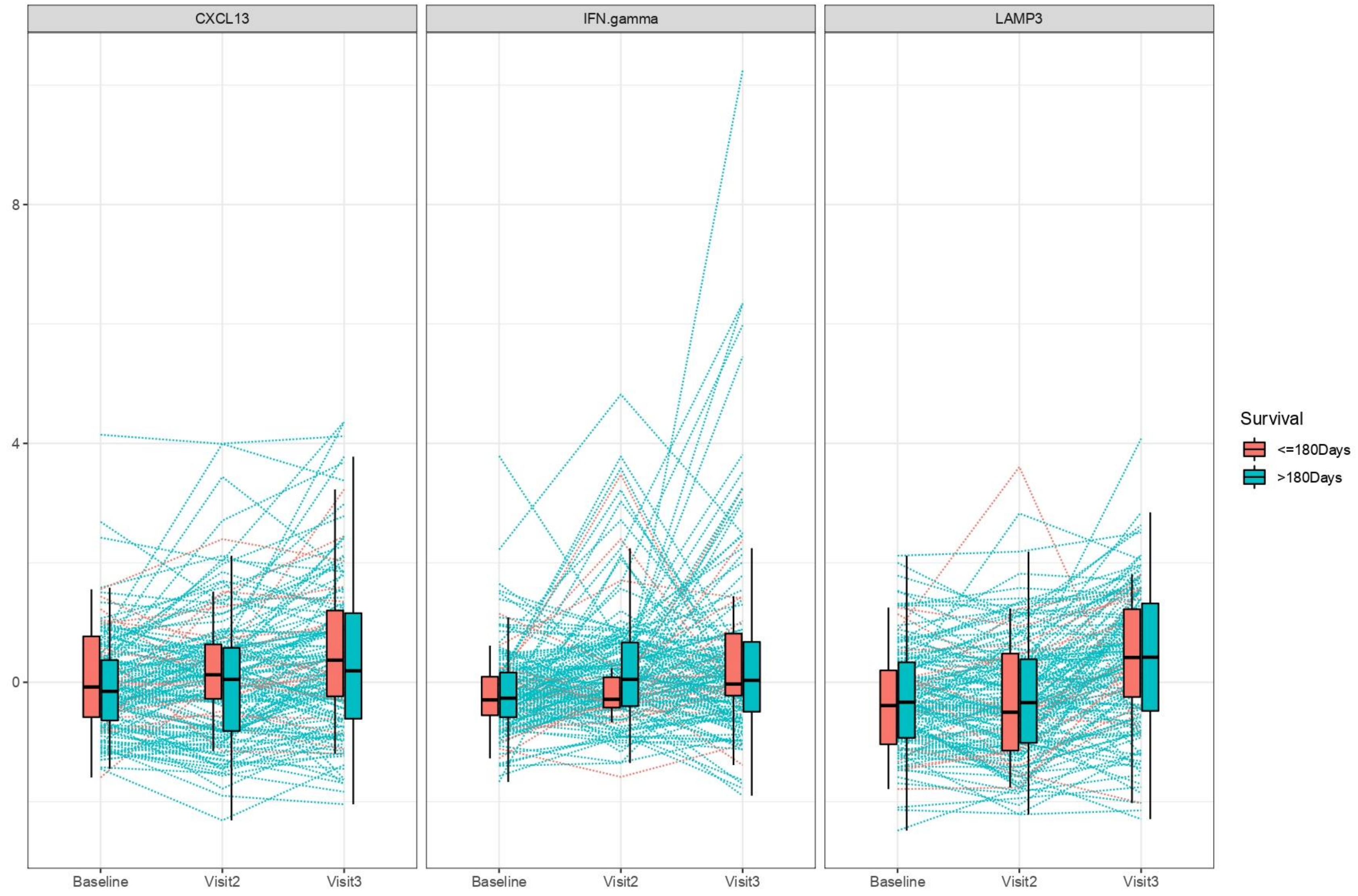

CXCL13
IFN.gamma
LAMP3
8
4
0
Baseline
Visit2
Visit3
Survival
<=180Days
>180Days

5) Cluster 5

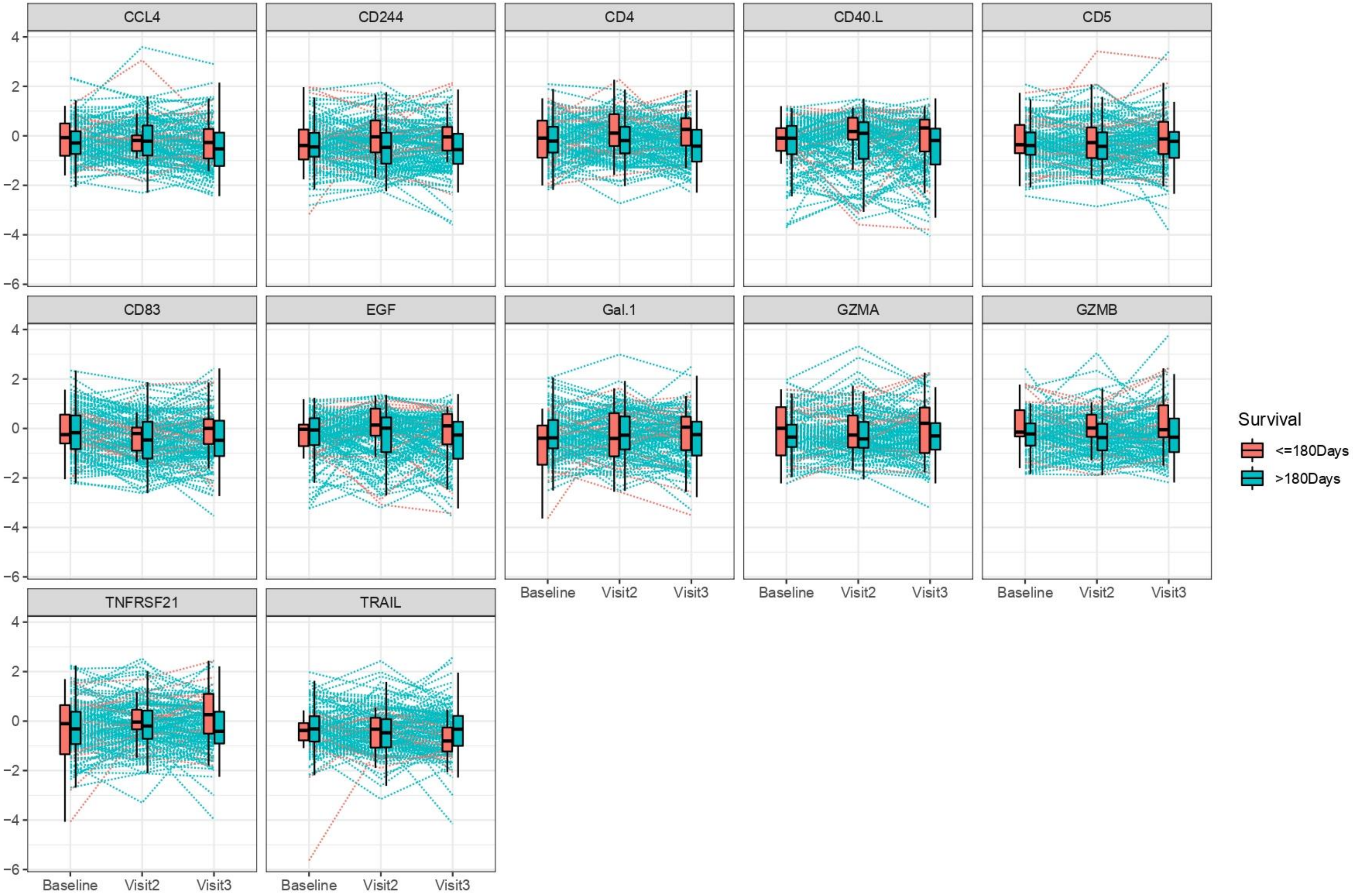
CCL4
CD244
CD4
CD40.L
CD5
CD83
EGF
Gal.1
GZMA
GZMB
TNFRSF21
TRAIL
Baseline
Visit2
Visit3
Survival
<=180Days
>180Days

6) Cluster 6

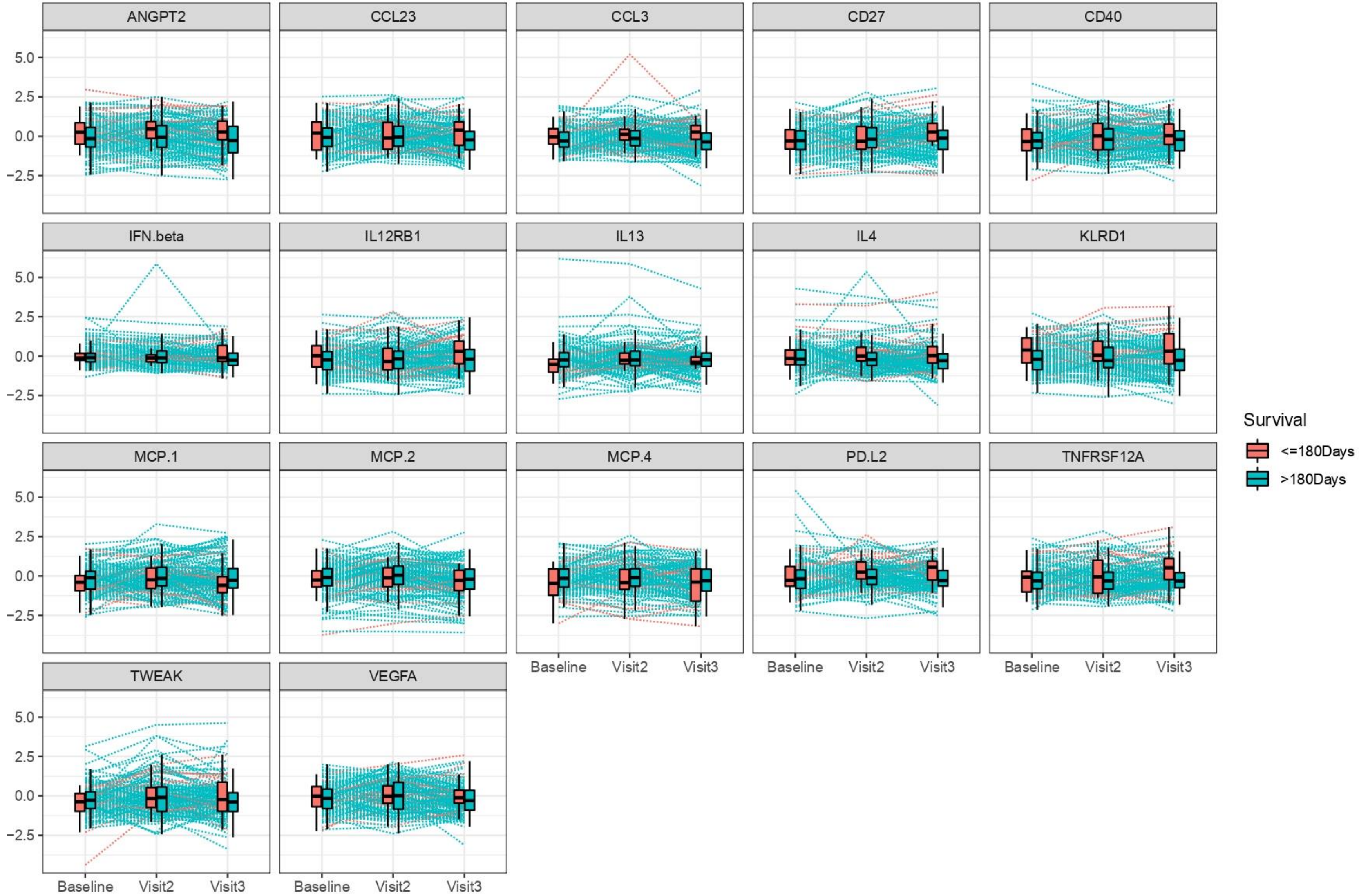
ANGPT2
CCL23
CCL3
CD27
CD40
IFN.beta
IL12RB1
IL13
IL4
KLRD1
MCP.1
MCP.2
MCP.4
PD.L2
TNFRSF12A
TWEAK
VEGFA
5.0
2.5
0.0
−2.5
Baseline
Visit2
Visit3
Survival
<=180Days
>180Days

7) Cluster 7

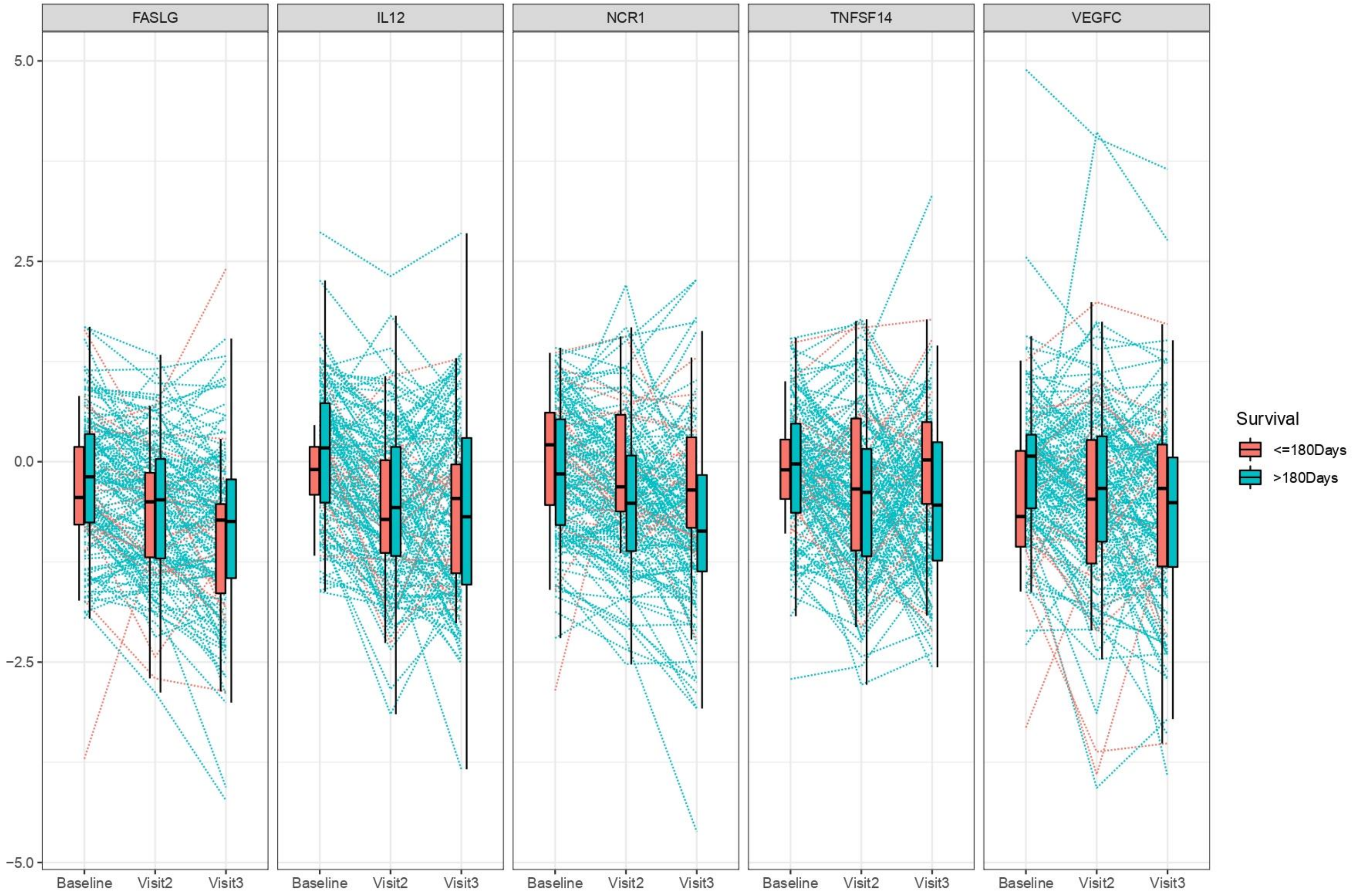
FASLG
IL12
NCR1
TNFSF14
VEGFC
5.0
2.5
0.0
−2.5
−5.0
Baseline
Visit2
Visit3
Survival
<=180Days
>180Days

8) Cluster 8

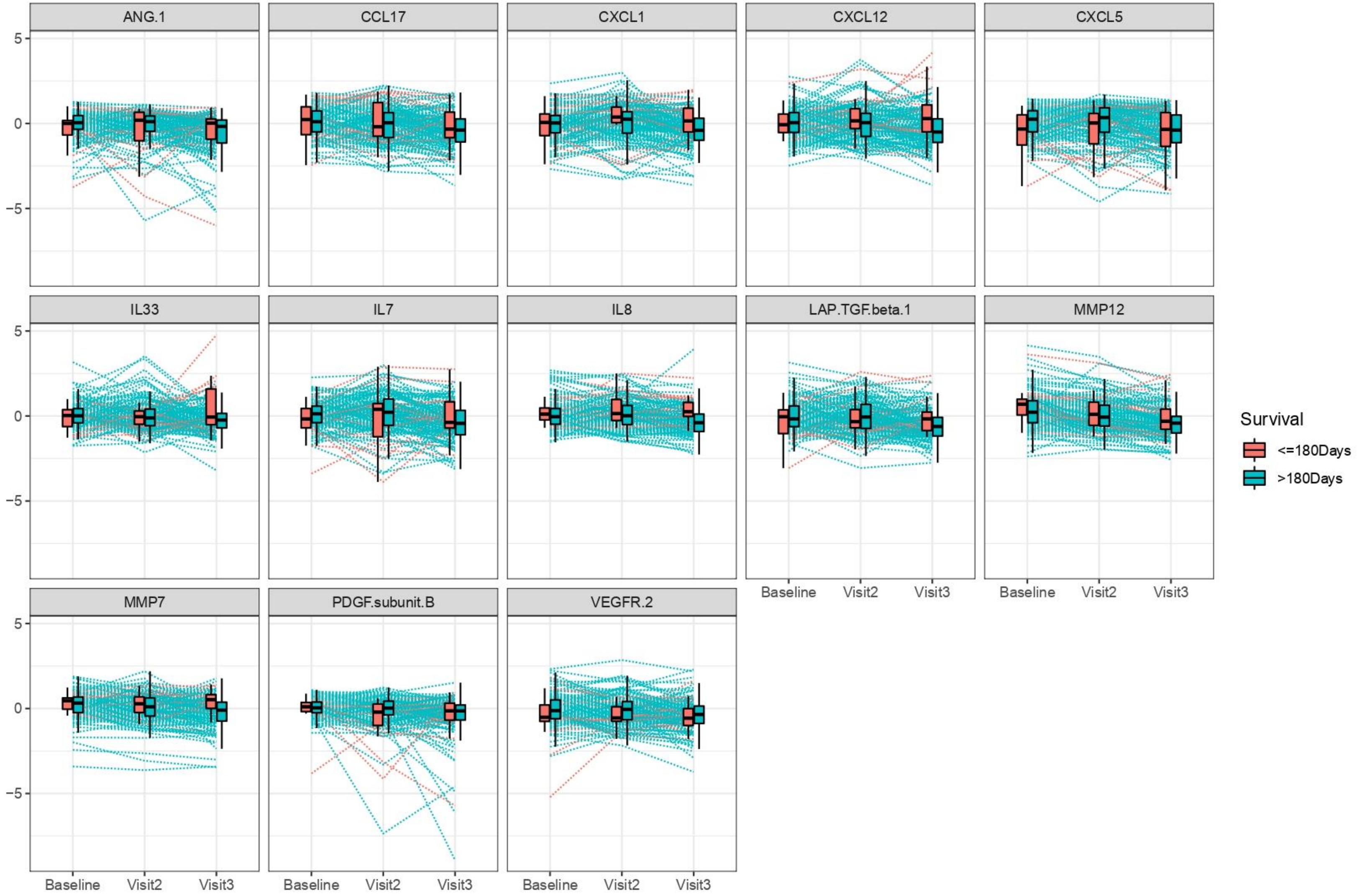
ANG.1
CCL17
CXCL1
CXCL12
CXCL5
IL33
IL7
IL8
LAP.TGF.beta.1
MMP12
MMP7
PDGF.subunit.B
VEGFR.2
5
0
-5
Baseline
Visit2
Visit3
Survival
<=180Days
>180Days

Supplementary Figure S8: Paired boxplots of the proteins over timepoints stratified by survival groups. Only proteins where the interaction between time and protein level gave a *P* value of <0.01 are shown.

a) CX3CL1, baseline to visit 2, ≤180 days vs. >180 days

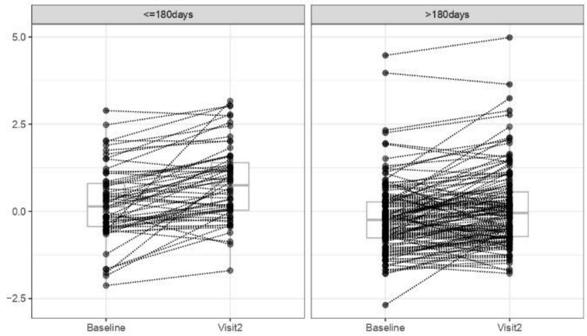

b) TNFRSF12A, baseline to visit 3, ≤180 days vs. >180 days

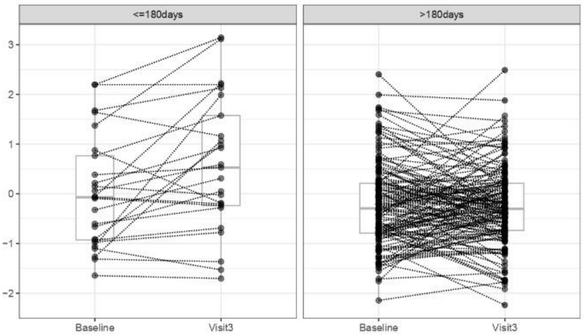

c) PD-L2, baseline to visit 3, ≤180 days vs. >180 days

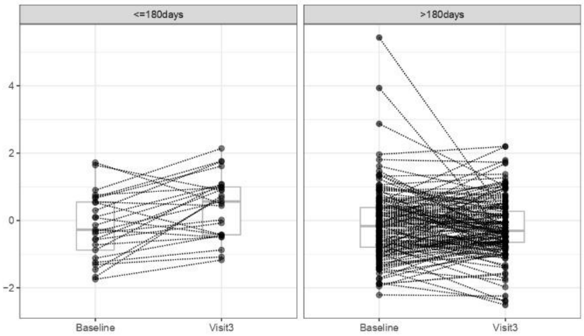

d) IL-7, baseline to visit 3, ≤180 days vs. >180 days

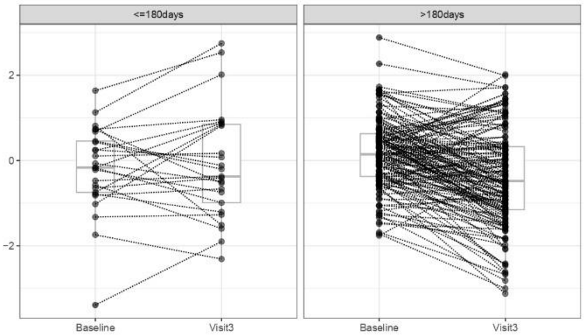

e) LAP TGF beta-1, baseline to visit 3, ≤180 days vs. >180 days

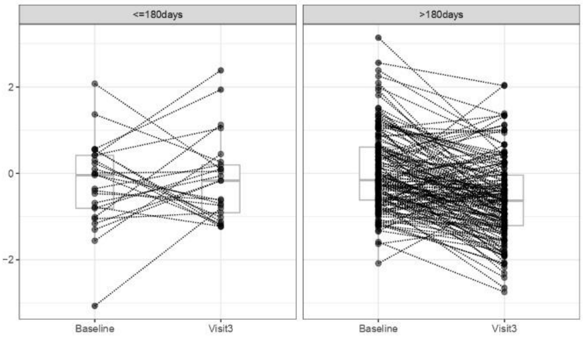

f) CXCL1, baseline to visit 3, ≤180 days vs. >180 days

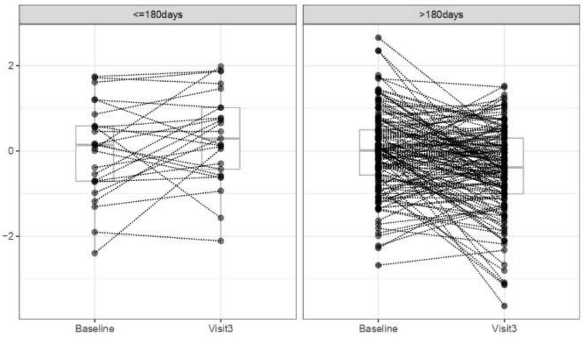

g) CXCL12, baseline to visit 3, ≤180 days vs. >180 days

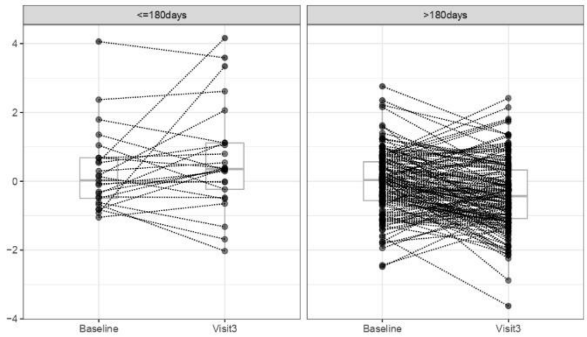

h) DCN, baseline to visit 3, ≤180 days vs. >180 days

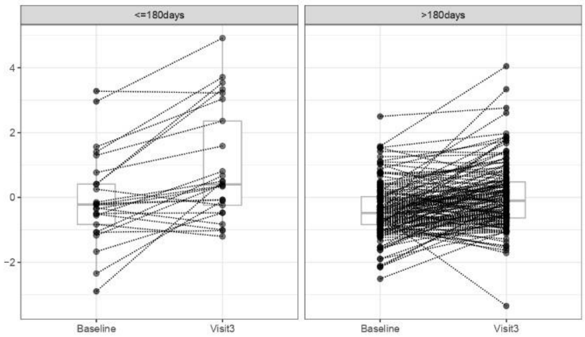

i) PD-L1, baseline to visit 3, ≤180 days vs. >180 days

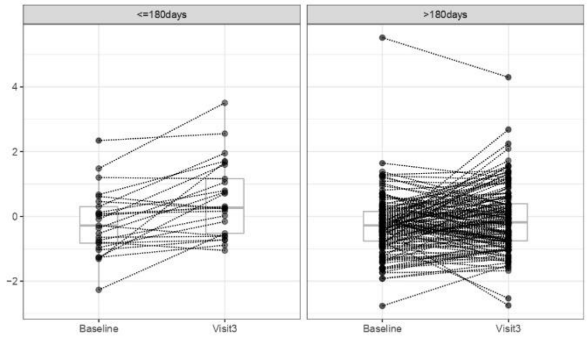

j) CXCL11, baseline to visit 3, ≤180 days vs. >180 days

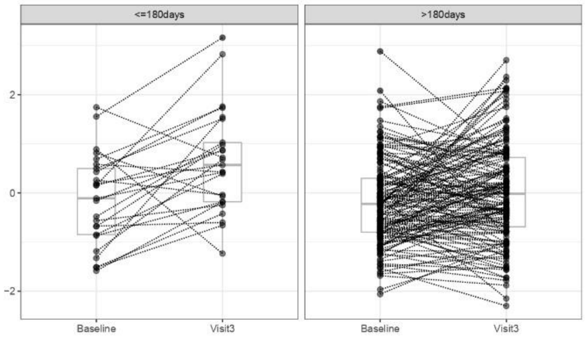

k) ADA, baseline to visit 3, ≤180 days vs. >180 days

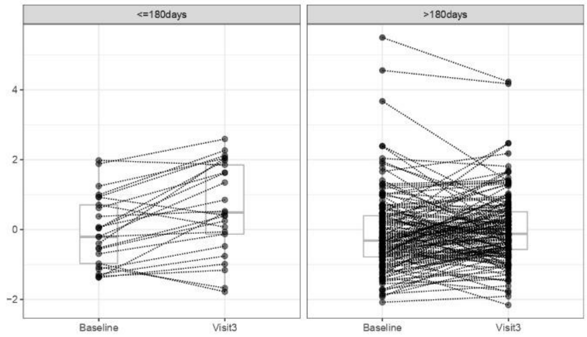

l) MMP7, baseline to visit 3, ≤1 year vs. >1 year

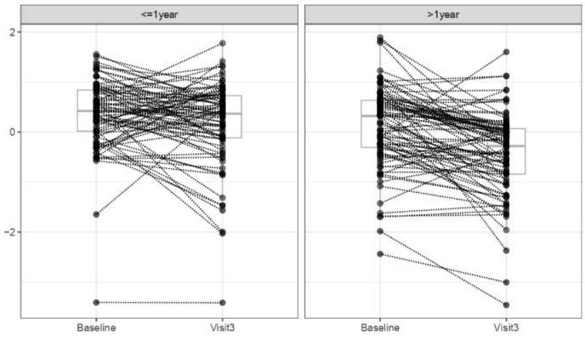

m) PTN, baseline to visit 3, ≤1 year vs. >1 year

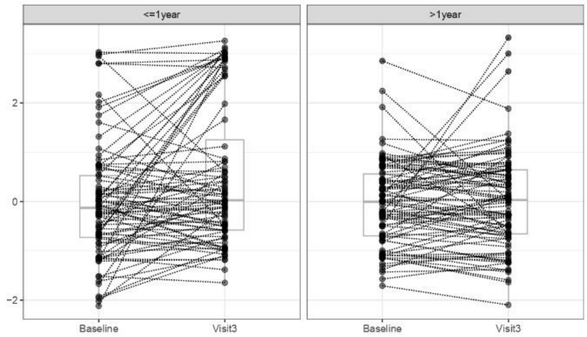

n) ARG1, baseline to visit 3, ≤1 year vs. >1 year

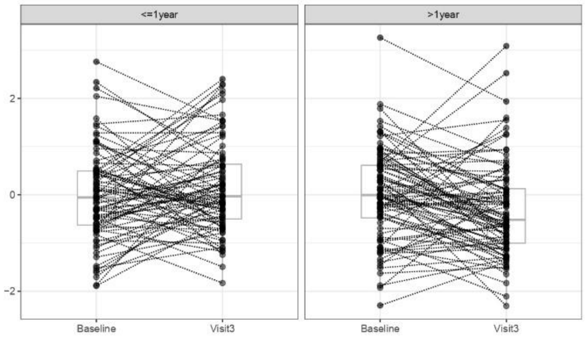

o) DCN, baseline to visit 3, ≤1 year vs. >1 year

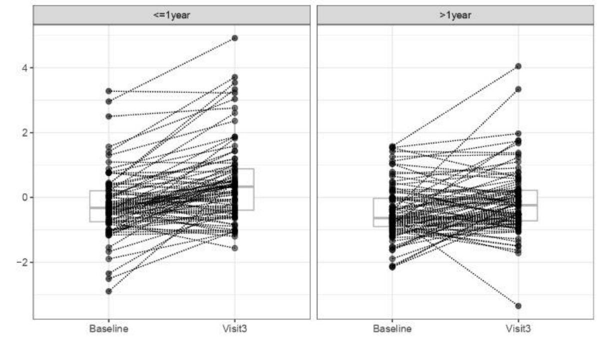

p) HGF, baseline to visit 3, ≤1 year vs. >1 year

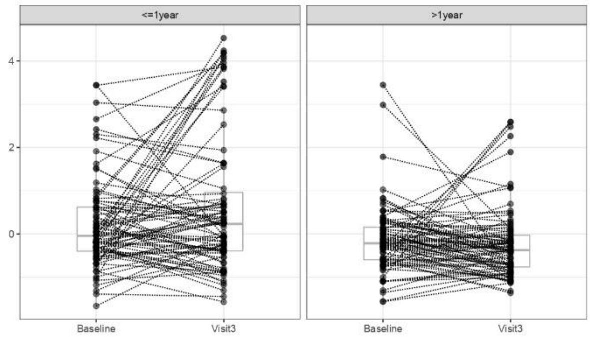

Supplementary Figure S9: Simplified representation of proteins where the longitudinal change in their levels was significantly ($P < 0.001$) associated with overall survival.

a) CD27, three timepoints

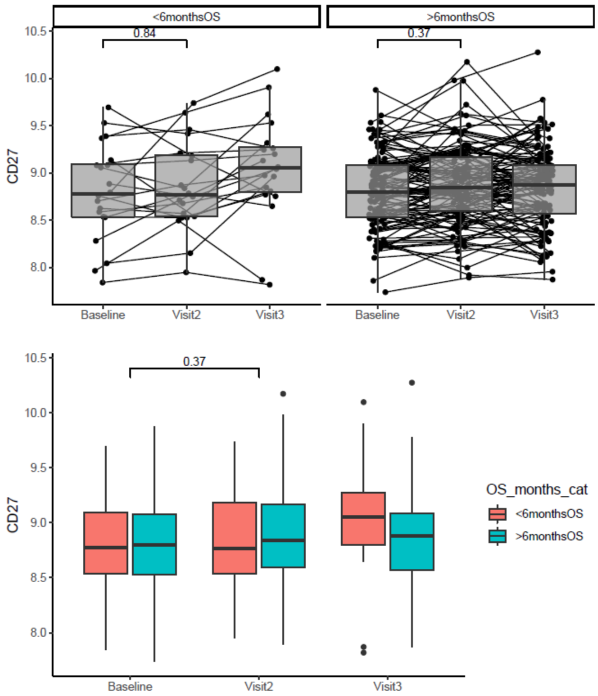

b) TNF, three timepoints

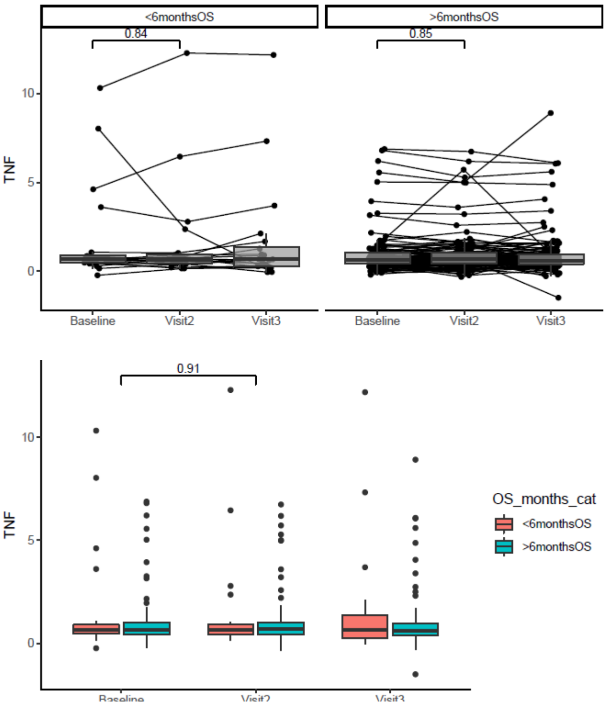

c) CAIX, two timepoints

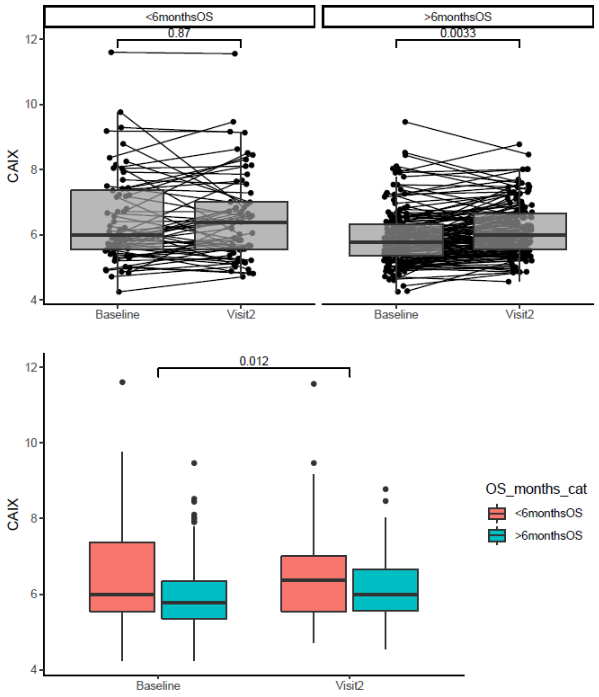

d) CX3CL1, two timepoints

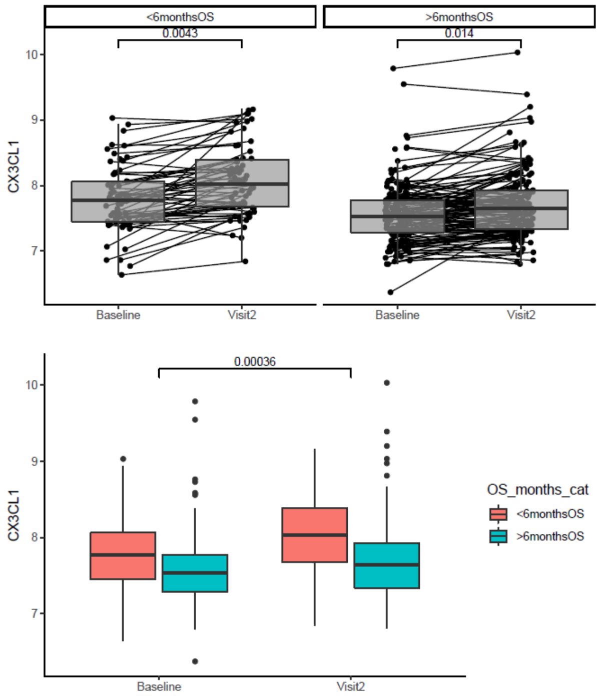

e) IL-6, two timepoints

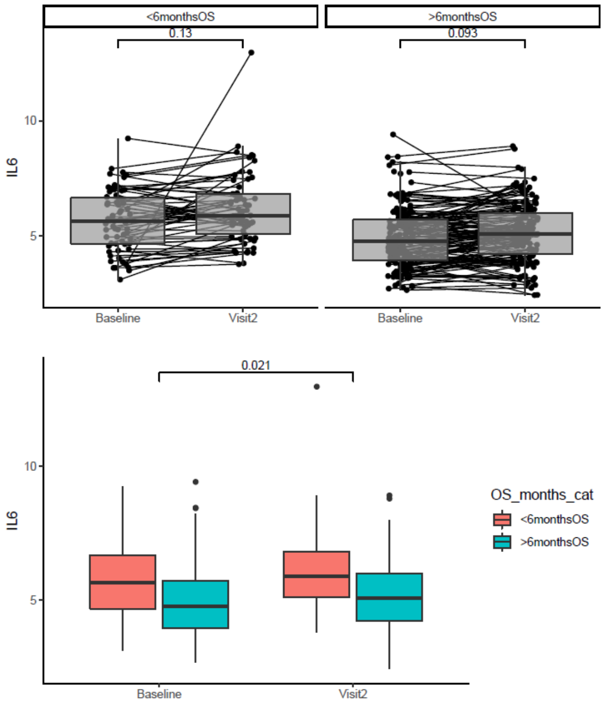

f) IL-8, two timepoints

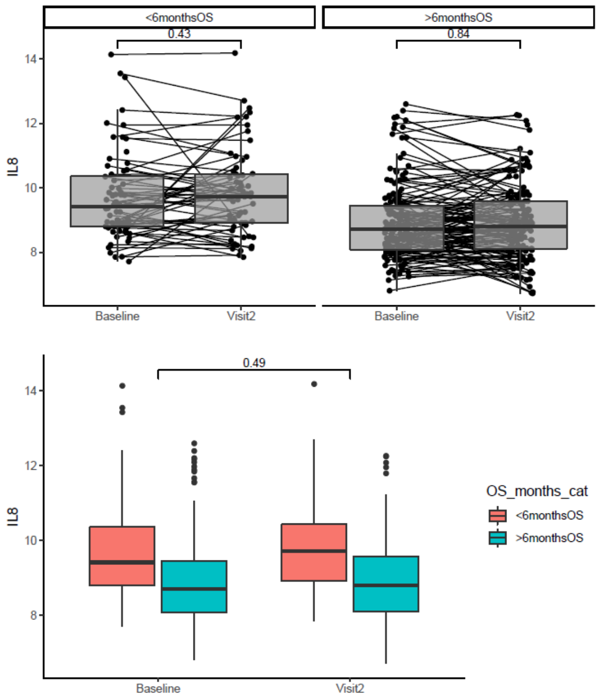

g) PDCD1, two timepoints

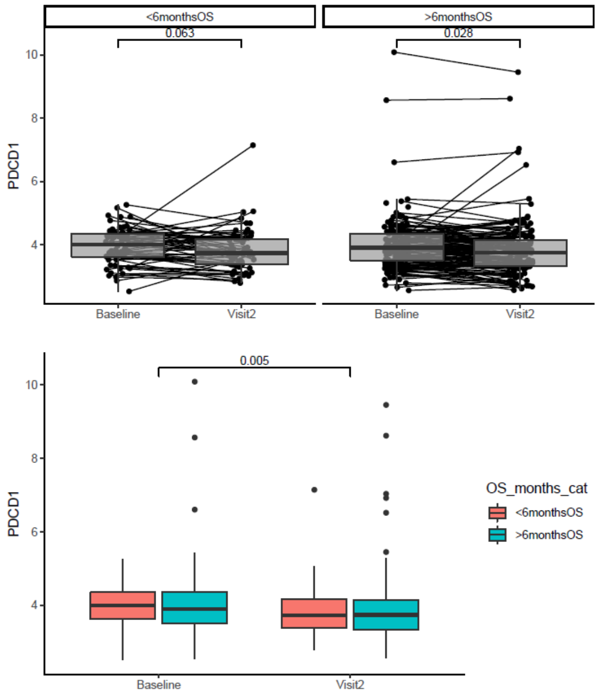

Supplementary Figure S10: Comparison of plasma IL-6 measurements using ELISA and Olink

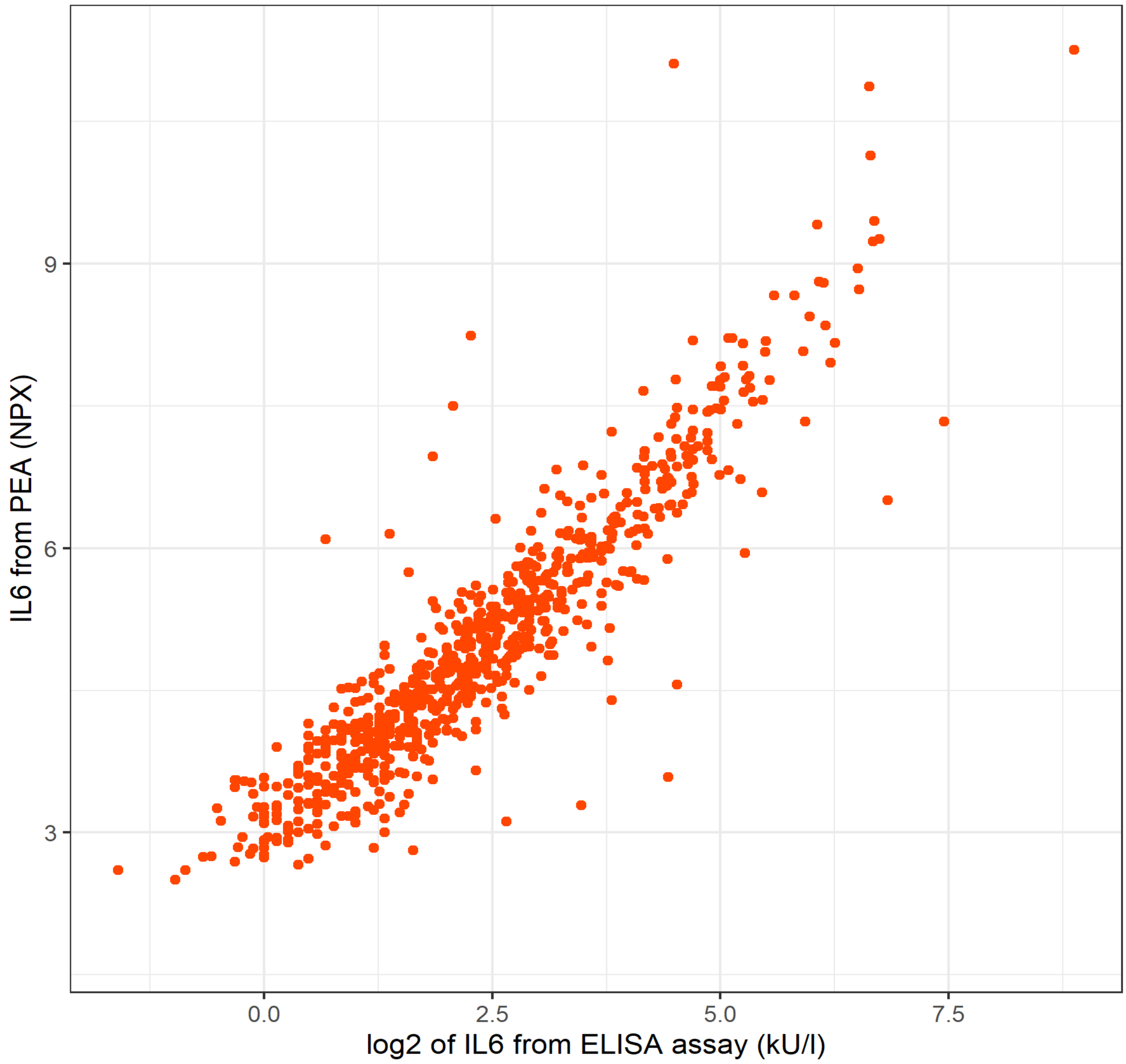

Supplementary Table S1: Full list of proteins in the immuno-oncology panel

Abbreviated protein names	Protein names	UniProt ID
ADA	Adenosine deaminase	P00813
ADGRG1	Adhesion G-protein coupled receptor G1	Q9Y653
ANG-1	Angiopoietin-1	Q15389
ANGPT2	Angiopoietin-2	O15123
ARG1	Arginase-1	P05089
CAIX	Carbonic anhydrase IX (CA9)	Q16790
CASP-8	Caspase-8	Q14790
CCL3	C-C motif chemokine 3	P10147
CCL4	C-C motif chemokine 4	P13236
CCL17	C-C motif chemokine 17	Q92583
CCL19	C-C motif chemokine 19	Q99731
CCL20	C-C motif chemokine 20	P78556
CCL23	C-C motif chemokine 23	P55773
CD4	T-cell surface glycoprotein CD4	P01730
CD5	T-cell surface glycoprotein CD5	P06127
CD8A	T-cell surface glycoprotein CD8 alpha chain	P01732
CD27	CD27 antigen	P26842
CD28	T-cell-specific surface glycoprotein CD28	P10747
CD40	CD40L receptor	P25942
CD40-L	CD40 ligand	P29965
CD70	CD70 antigen	P32970
CD83	CD83 antigen	Q01151
CD244	Natural killer cell receptor 2B4	Q9BZW8
CRTAM	Cytotoxic and regulatory T-cell molecule	O95727
CSF-1	Macrophage colony-stimulating factor 1	P09603
CX3CL1	Fractalkine	P78423
CXCL1	C-X-C motif chemokine 1	P09341
CXCL5	C-X-C motif chemokine 5	P42830
CXCL9	C-X-C motif chemokine 9	Q07325
CXCL10	C-X-C motif chemokine 10	P02778
CXCL11	C-X-C motif chemokine 11	O14625
CXCL12	Stromal cell-derived factor 1	P48061
CXCL13	C-X-C motif chemokine 13	O43927
DCN	Decorin	P07585
EGF	Pro-epidermal growth factor	P01133
FASLG	Fas Ligand/Tumor necrosis factor ligand superfamily member 6	P48023
FGF2	Fibroblast growth factor 2	P09038
Gal-1	Galectin-1	P09382
Gal-9	Galectin-9	O00182
GZMA	Granzyme A	P12544
GZMB	Granzyme B	P10144
GZMH	Granzyme H	P20718
HGF	Hepatocyte growth factor	P14210

HO-1	Heme oxygenase 1	P09601
ICOSLG	ICOS ligand	O75144
IFN-beta	Interferon beta	P01574
IFN-gamma	Interferon gamma	P01579
IL-1 alpha	Interleukin-1 alpha	P01583
IL-2	Interleukin-2	P60568
IL-4	Interleukin-4	P05112
IL-5	Interleukin-5	P05113
IL-6	Interleukin-6	P05231
IL-7	Interleukin-7	P13232
IL-8	Interleukin 8	P10145
IL-10	Interleukin-10	P22301
IL-12	Interleukin-12	P29459, P29460
IL-12RB1	Interleukin-12 receptor subunit beta-1	P42701
IL-13	Interleukin-13	P35225
IL-18	Interleukin-18	Q14116
IL-21	Interleukin-21	Q9HBE4
IL-33	Interleukin-33	O95760
IL-35	Interleukin-35	Q14213, P29459
KLRD1	Natural killer cells antigen CD94	Q13241
LAMP3	Lysosome-associated membrane glycoprotein 3	Q9UQV4
LAP TGF-beta-1	Latency-associated peptide transforming growth factor beta-1	P01137
MCP-1	Monocyte chemotactic protein 1	P13500
MCP-2	Monocyte chemotactic protein 2	P80075
MCP-3	Monocyte chemotactic protein 3	P80098
MCP-4	Monocyte chemotactic protein 4	Q99616
MIC-A/B	MHC class I polypeptide-related sequence A/B	Q29983, Q29980
MMP7	Matrix metalloproteinase-7	P09237
MMP12	Macrophage metalloproteinase-12	P39900
NCR1	Natural cytotoxicity triggering receptor	O76036
NOS3	Nitric oxide synthase, endothelia	P29474
PDCD1	Programmed cell death protein 1	Q15116
PDGF subunit B	Platelet-derived growth factor subunit B	P01127
PD-L1	Programmed cell death 1 ligand 1	Q9NZQ7
PD-L2	Programmed cell death 1 ligand 2	Q9BQ51
PGF	Placenta growth factor	P49763
PTN	Pleiotrophin	P21246
TIE2	Angiopoietin-1 receptor	Q02763
TNF	Tumor necrosis factor	P01375
TNFRSF4	Tumor necrosis factor receptor superfamily member 4	P43489
TNFRSF9	Tumor necrosis factor receptor superfamily member 9	Q07011
TNFRSF12A	Tumor necrosis factor receptor superfamily member 12A	Q9NP84
TNFRSF21	Tumor necrosis factor receptor superfamily member 21	O75509
TNFSF14	Tumor necrosis factor ligand superfamily member 14	O43557
TRAIL	TNF-related apoptosis-inducing ligand	P50591
TWEAK	Tumor necrosis factor ligand superfamily member 12	O43508
VEGFA	Vascular endothelial growth factor A	P15692
VEGFC	Vascular endothelial growth factor C	P49767
VEGFR-2	Vascular endothelial growth factor receptor 2	P35968

Supplementary Table S2: Proteins found to be differentially expressed in baseline samples in all patients divided by survival <90 days (n = 57) and >2 years (n = 30), only showing proteins for which the comparison gave P values of <0.05

Protein	Test	P value	Adjusted P value	Median NPX for group with Survival ≤ 90 days	Median NPX for group with Survival >2 years	log2 fold change
IL-6	*t* test	$9.2x10^{-14}$	$9.0x10^{-12}$	6.7	4.00	2.19
TNFRSF12A	Wilcoxon	$1.2x10^{-08}$	$3.1x10^{-07}$	7.6	6.90	0.78
CSF-1	*t* test	$5.6x10^{-08}$	$1.3x10^{-06}$	9.2	8.98	0.27
IL-8	Wilcoxon	$6.4x10^{-08}$	$1.3x10^{-06}$	9.9	8.37	1.44
CCL23	*t* test	$5.8x10^{-07}$	$7.8x10^{-06}$	12.2	11.48	0.59
HGF	Wilcoxon	$1.2x10^{-06}$	$1.5x10^{-05}$	10.0	9.49	0.76
ANGPT2	*t* test	$1.9x10^{-06}$	$2.2x10^{-05}$	7.4	6.94	0.55
PD-L1	*t* test	$1.9x10^{-05}$	$1.6x10^{-04}$	5.9	5.64	0.42
MCP-3	Wilcoxon	$2.0x10^{-05}$	$1.6x10^{-04}$	4.7	3.51	0.90
TNFRSF21	*t* test	$2.8x10^{-05}$	$2.1x10^{-04}$	9.3	9.07	0.24
PGF	Wilcoxon	$3.9x10^{-05}$	$2.5x10^{-04}$	9.3	8.95	0.38
MMP7	Wilcoxon	$5.7x10^{-05}$	$3.6x10^{-04}$	12.3	12.03	0.23
CA19-9	Wilcoxon	$1.2x10^{-04}$	$6.0x10^{-04}$	12.1	6.64	3.89
VEGFA	*t* test	$1.2x10^{-04}$	$6.2x10^{-04}$	10.5	9.87	0.52
IL-10	Wilcoxon	$2.6x10^{-04}$	$1.2x10^{-03}$	5.0	4.27	0.80
TNFRSF4	*t* test	$3.0x10^{-04}$	$1.4x10^{-03}$	5.3	4.90	0.37
CASP-8	*t* test	$3.1x10^{-04}$	$1.4x10^{-03}$	7.1	6.42	0.70
TIE2	Wilcoxon	$3.1x10^{-04}$	$1.4x10^{-03}$	8.8	8.66	0.22
TRAIL	*t* test	$3.5x10^{-04}$	$1.5x10^{-03}$	8.5	8.73	-0.30
CCL3	*t* test	$3.9x10^{-04}$	$1.6x10^{-03}$	7.4	7.03	0.42
CX3CL1	Wilcoxon	$4.4x10^{-04}$	$1.7x10^{-03}$	7.8	7.40	0.40
CCL20	Wilcoxon	$5.6x10^{-04}$	$2.0x10^{-03}$	8.9	7.32	1.39
MMP12	*t* test	$5.6x10^{-04}$	$2.0x10^{-03}$	7.7	6.68	0.85
CXCL13	*t* test	$8.4x10^{-04}$	$2.9x10^{-03}$	10.0	9.48	0.45
CXCL1	Wilcoxon	$8.5x10^{-04}$	$2.9x10^{-03}$	11.3	10.92	0.39
ADA	*t* test	$1.6x10^{-03}$	$5.4x10^{-03}$	3.9	3.46	0.34
Gal-9	*t* test	$1.9x10^{-03}$	$5.8x10^{-03}$	9.1	8.91	0.24
MIC-A/B	Wilcoxon	$1.9x10^{-03}$	$5.8x10^{-03}$	6.3	5.67	0.63
CD40	*t* test	$3.7x10^{-03}$	$1.0x10^{-02}$	12.3	11.98	0.34
VEGFC	*t* test	$5.3x10^{-03}$	$1.5x10^{-02}$	3.1	3.44	-0.33
CD4	Wilcoxon	$5.4x10^{-03}$	$1.5x10^{-02}$	2.1	1.98	0.25
NOS3	*t* test	$5.7x10^{-03}$	$1.5x10^{-02}$	1.1	0.81	0.35
TNFSF14	*t* test	$5.7x10^{-03}$	$1.5x10^{-02}$	7.2	6.70	0.52
GZMH	*t* test	$7.4x10^{-03}$	$1.9x10^{-02}$	6.1	5.73	0.56
ADGRG1	Wilcoxon	$8.3x10^{-03}$	$2.1x10^{-02}$	3.2	2.56	0.48
IL-12RB1	*t* test	$1.1x10^{-02}$	$2.6x10^{-02}$	3.1	2.88	0.24
MCP-1	Wilcoxon	$1.7x10^{-02}$	$4.0x10^{-02}$	12.4	12.32	0.41
ICOSLG	Wilcoxon	$2.0x10^{-02}$	$4.6x10^{-02}$	6.4	6.64	-0.19
CAIX	Wilcoxon	$2.0x10^{-02}$	$4.6x10^{-02}$	6.4	5.98	0.79
CXCL11	*t* test	$2.1x10^{-02}$	$4.6x10^{-02}$	8.8	8.60	0.49

Abbreviations: Wilcoxon = Wilcoxon Rank Sum Test

Supplementary Table S3: Candidate prognostic circulating protein signatures with associated proportion scores. Index I corresponds to signature 7.

Abbreviated protein names	Signatures										
	1	2	3	4	5	6	7	8	9	10	11
ADGRG1	All 93 markers	X									
ARG1		X									
CASP-8		X									
CCL17		X									
CCL19		X									
CCL20		X	X	X	X	X					
CCL23		X									
CD40-L		X									
CD70		X									
CSF-1		X	X	X	X	X	X	X	X		
CX3CL1		X									
CXCL1		X	X								
CXCL13		X									
HO-1		X	X	X	X	X					
ICOSLG		X	X								
IFN-gamma		X									
IL-1 alpha		X	X	X							
IL-2		X	X								
IL-4		X									
IL-6		X	X	X	X	X	X	X	X	X	X
IL-7		X									
IL-8		X	X	X	X	X					
IL-10		X									
IL-21		X									
LAP TGF beta-1		X									
MCP-1		X									
MCP-2		X	X	X							
MCP-3		X	X	X							
MCP-4		X									
MIC-A/B		X									
MMP7		X	X	X	X						
PD-L1		X	X	X	X	X					
PD-L2		X	X								
PDCD1		X	X	X	X	X	X				
TNFRSF12A		X	X	X	X	X	X	X	X	X	
TNFRSF21		X	X	X							
TNFSF14		X									
TRAIL		X	X	X	X	X	X				
TWEAK		X	X	X	X	X	X				
VEGFA		X	X	X							
VEGFR-2		X	X								
CA19-9		X	X	X	X	X	X	X			
**Proteins, n**	**93**	**42**	**22**	**17**	**12**	**11**	**7**	**4**	**3**	**2**	**1**

Proportion score	0	0.05	0.1	0.15	0.2	0.25	0.35	0.4	0.5	0.65	0.9

Supplementary Table S4: The relation to cancer and inflammation of the proteins included in the two indices

Proteins marked with yellow are in Index I. Proteins marked with blue are in Index II. Proteins marked with green are in both Index I and Index II.

Known relation to / Abbrevi-ated protein names	Biological process	Cancer	Inflammation and other	Associated with
**CSF-1**<br><br>= Macrophage colony-stimulating factor (M-CSF)	Suppress tumor immunity	Cytokine overexpressed by human **PDAC cells** [48]. Increased production of CSF-1 by the cancer-associated fibroblasts in **PDAC** contributes to the M2 polarization of tumor-associated macrophages enhancing pancreatic cancer growth and progression [49].	Regulates the survival, proliferation, and differentiation of mononuclear phagocytes from precursors to fully differentiated macrophages [50].	
**CXCL13**<br><br>= B lymphocyte chemoattractant (BLC)	Chemotaxis<br><br>Promote tumor immunity<br><br>Suppress tumor immunity	Chemokine interacting with the receptor CXCR5 which is present on B cells and some tumor cells. Has been implicated as key modulators of both tumor progression and antitumor immunity [51]. CXCL13 can drive tumor growth and invasion through PI3K/AKT signaling or contribute to an enhanced antitumor immune response via increased tumor immune localization [51]. Has been associated with both metastasis and with greater patient survival [51]. Has been shown to be overexpressed in **pancreatic cancer cell lines** [52].	Expressed by follicular dendritic cells and helper T cells and is essential for naive B cell homing and organization within lymphoid follicles, sites critical for B cell antigen interaction and B cell differentiation [51]. Implicated in the pathogenesis of a number of autoimmune diseases and inflammatory conditions, as well as in lymphoproliferative disorders [52].	
**IL-6**	Promote tumor immunity<br><br>Suppress tumor immunity	Produced in various types of tumor cells and is found to be elevated in many types of cancers [53]. Known to be significantly increased in **pancreatic cancer cells** compared to normal pancreatic cells [30]. Has been	Cytokine released at the local lesion of early inflammation. Induces synthesis of acute phase reactants such as CRP and plays an important role in acquired immune response by stimulation of antibody production and of effector T-	VEGF, IL-8, IL-10, HGF

		shown to be significantly higher in **PDAC** patients compared to healthy individuals and patients with chronic pancreatitis [54]. Associated with short OS in patients with **PDAC** [30,55]. Has more recently been shown to be a prognostic factor in melanoma and renal and colorectal cancer [56–58].	cell development. In the bone marrow, IL-6 stimulates maturation of megakaryocytes leading to a release of platelets [59]. IL-6 is overexpressed in many chronic inflammatory diseases, and also induces excess production of VEGF leading to enhanced angiogenesis and increased vascular permeability, which are pathological features of inflammatory lesions [59,60].	
**PDCD1**<br>= PD-1	Suppress tumor immunity	The ligands of PDCD1 are upregulated by tumor cells, which promotes inhibition of both the innate and the adaptive immune response [31]. Involved in immune evasion and tumor progression [61]. Prognostic marker in multiple cancer types [62]. Expressed in **pancreatic cancer cells** [63].	Involved in the regulation of immune responses [31]. Binds to its two known ligands PD-L1 and PD-L2 [31,61]. Is one of the T-cell co-inhibitory receptors expressed on multiple types of immune cells [61].	PD-L1, PD-L2
**TNFRSF12A**<br>= TWEAK receptor, fibroblast growth factor–inducible 14 (Fn14)<br>**TWEAK**	Vascular/tissue remodeling	Overexpressed in many solid tumor types, including **pancreatic cancer** and cholangiocarcinoma [36,64,65]. Both TWEAK and TNFRSF12A show low expression in normal tissues but are highly expressed in many carcinomas and metastases and are associated with a worse clinical outcome [35].	Upon binding of TWEAK to TNFRSF12A, a number of intracellular signaling pathways are activated, including NF-κB, [36] and this interaction promotes cell proliferation, migration, differentiation, apoptosis, angiogenesis, and inflammation in various cell types [36,66].	
**TRAIL**	Apoptosis/cell killing	Known to activate non-apoptotic signaling pathways in cancer cell lines, such as NF-κB, MAP-K and ERK [67,68]. Stimulates tumor invasion and metastasis in the presence of oncogenic KRAS-mutations in colorectal cancer cell lines [69]. Its receptor, TRAIL-R2, has been shown to be highly expressed on **PDAC** human tissue and stimulation promoted cancer progression, invasion, and metastasis [67].	TRAIL is a cytokine and is expressed mainly by cells of the immune system [70].	CASP-8, FASLG

**CA19-9**	Tumor marker/ cell-to-cell recognition	*Please see main article*	*Please see main article*

Abbreviations: CRP: C-reactive Protein; PD-1: Programmed cell death protein 1.

Supplementary Table S5: Candidate prognostic plasma protein signatures from the following comparisons: OS ≤90 days vs. >90 days, OS ≤180 days vs. >180 days, and OS <90 days vs. >1 year.

Abbreviated	OS ≤90 days vs. >90 days									OS ≤180 days vs. >180 days											OS <90 days vs. >1 year											
protein names	A1	A2	A3	A4	A5	A6	A7	A8	A9	B1	B2	B3	B4	B5	B6	B7	B8	B9	B10	B11	C1	C2	C3	C4	C5	C6	C7	C8	C9	C10	C11	C12
ADA										X																						
ADGRG1	X	X	X	X	X					X	X	X	X	X	X	X						X	X	X	X							
ANGPT2	X	X								X	X	X	X	X	X							X	X	X	X							
CAIX	X	X	X	X	X	X	X			X	X	X	X	X	X	X	X	X	X			X	X	X								
CASP-8										X	X	X																				
CCL3										X																						
CCL19																						X										
CCL20	X	X	X	X	X	X				X	X	X	X	X	X	X	X	X				X	X	X	X	X	X	X				
CCL23	X	X	X	X	X					X	X																					
CD244																						X										
CD27	X	X																														
CD4	X	X								X																						
CD40	X									X																						
CD70																						X										
CD8A																					All 93	X	X									
CSF-1	X	X	X	X	X	X	X			X	X	X	X	X	X	X	X					X	X	X	X	X	X	X	X			
CX3CL1	X									X												X	X	X								
CXCL1	X	X								X																						
CXCL13										X	X	X										X										
DCN	X	X								X	X	X																				
FGF2																						X										
GZMB																						X										
HGF	X	X	X	X	X	X				X												X	X	X								
HO-1										X	X	X										X										
ICOSLG	X	X	X	X																												
IFN-gamma																						X										
IL-1-alpha																						X	X	X	X							
IL-2																						X										

IL-5																						X										
IL-6	X	X	X	X	X	X	X	X	X	X	X	X	X	X	X	X	X	X	X	X		X	X	X	X	X	X	X	X	X	X	X
IL-8	X									X												X	X	X	X	X						
IL-10										X	X	X	X	X								X	X									
IL-12RB1	X									X																						
IL-13																						X	X	X								
IL-21																						X	X									
KLRD1										X	X	X	X	X																		
MCP-1	X	X								X																						
MCP-3	X	X	X							X	X	X	X	X	X							X										
MCP-4																						X										
MIC-A/B																						X										
MMP12	X	X	X	X	X	X				X	X											X										
MMP7	X	X	X	X						X	X	X	X	X	X	X	X	X														
NOS3	X									X	X	X	X	X	X							X	X	X	X	X	X	X				
PD-L1	X									X	X	X																				
PD-L2										X	X	X																				
PDCD1																						X	X									
PDGF subunit-B										X	X	X	X									X										
PGF	X	X								X	X	X										X	X	X	X	X	X					
TIE2										X	X																					
TNF																						X										
TNFRSF12A	X	X	X	X						X												X	X	X	X	X	X	X	X	X	X	
TNFRSF21	X									X												X	X	X	X	X						
TNFRSF4										X																						
TNFSF14	X									X	X											X	X									
TRAIL	X	X	X	X	X	X	X	X		X	X	X	X	X	X	X	X	X				X	X	X	X	X	X	X				
TWEAK																						X	X	X	X	X	X	X	X	X		
VEGFA	X									X	X											X										
VEGFR-2																						X	X	X	X	X						
CA19-9																						X	X	X	X	X	X	X				
**Proteins, n**	**29**	**20**	**13**	**12**	**9**	**7**	**4**	**2**	**1**	**38**	**25**	**20**	**13**	**12**	**10**	**7**	**6**	**5**	**2**	**1**	**93**	**41**	**24**	**19**	**15**	**12**	**9**	**8**	**4**	**3**	**2**	**1**

**Proportion score**	0	0.05	0.1	0.2	0.3	0.25	0.35	0.5	0.9	0	0.05	0.10	0.15	0.20	0.25	0.35	0.40	0.45	0.65	0.90	0.00	0.05	0.10	0.15	0.20	0.25	0.30	0.40	0.50	0.55	0.75	0.95

Supplementary Table S6: Performance of the prognostic plasma protein signatures from the comparisons OS ≤90 days vs. >90 days (A1–A9), OS ≤180 days vs. >180 days (B1–B11), and OS <90 days vs. 1 year (C1–C12).

Signature	Discovery cohort (*n* = 215)					Replication cohort (*n* = 148)					Replication cohort when adding age to the model (*n* = 148)				
	**AUC (95% CI)**	**BPsens (95% CI)**	**BPspec (95% CI)**	**PPV (95% CI)**	**NPV (95% CI)**	**AUC (95% CI)**	**BPsens (95% CI)**	**BPspec (95% CI)**	**PPV (95% CI)**	**NPV (95% CI)**	**AUC (95% CI)**	**BPsens (95% CI)**	**BPspec (95% CI)**	**PPV (95% CI)**	**NPV (95% CI)**
**A1**	0.76 (0.65–0.88)	0.72 (0.54–1)	0.72 (0.42–0.92)	0.41 (0.30–0.7)	0.91 (0.87–1)	0.76 (0.65–0.86)	0.52 (0.43–1)	0.90 (0.39–0.96)	0.50 (0.22–0.73)	0.91 (0.89–1)	0.76 (0.65–0.86)	0.52 (0.43–1)	0.90 (0.40–0.96)	0.50 (0.22–0.73)	0.91 (0.89–1)
**A2**	0.77 (0.66–0.88)	0.95 (0.63–1)	0.54 (0.47–0.89)	0.35 (0.31–0.62)	0.97 (0.89–1)	0.76 (0.66–0.87)	0.52 (0.43–1)	0.89 (0.38–0.96)	0.48 (0.22–0.73)	0.91 (0.89–1)	0.76 (0.66–0.87)	0.52 (0.43–1)	0.89 (0.38–0.96)	0.48 (0.22–0.72)	0.91 (0.90–1)
**A3**	0.81 (0.70–0.91)	0.77 (0.63–1)	0.77 (0.50–0.89)	0.47 (0.33–0.65)	0.92 (0.90–1)	0.77 (0.67–0.87)	0.52 (0.47–1)	0.89 (0.4–0.95)	0.48 (0.22–0.7)	0.91 (0.90–1)	0.77 (0.67–0.87)	0.52 (0.47–1)	0.89 (0.40–0.95)	0.48 (0.22–0.7)	0.91 (0.90–1)
**A4**	0.80 (0.69–0.91)	0.77 (0.63–1)	0.76 (0.54–0.89)	0.45 (0.33–0.62)	0.92 (0.89–1)	0.77 (0.66–0.87)	0.52 (0.43–1)	0.90 (0.39–0.95)	0.50 (0.22–0.7)	0.91 (0.90–1)	0.77 (0.66–0.87)	0.52 (0.43–1)	0.90 (0.39–0.96)	0.50 (0.22–0.7)	0.91 (0.90–1)
**A5**	0.82 (0.72–0.91)	0.81 (0.68–1)	0.77 (0.54–0.87)	0.48 (0.35–0.62)	0.94 (0.90–1)	0.75 (0.65–0.86)	0.78 (0.43–1)	0.60 (0.36–0.95)	0.26 (0.21–0.66)	0.93 (0.89–1)	0.76 (0.65–0.86)	0.78 (0.43–1)	0.62 (0.38–0.95)	0.27 (0.22–0.66)	0.93 (0.90–1)
**A6**	0.81 (0.72–0.90)	0.90 (0.68–1)	0.63 (0.54–0.87)	0.39 (0.33–0.61)	0.96 (0.90–1)	0.76 (0.66–0.86)	0.73 (0.47–1)	0.71 (0.39–0.93)	0.32 (0.22–0.62)	0.93 (0.90–1)	0.76 (0.66–0.86)	0.73 (0.47–1)	0.71 (0.40–0.93)	0.32 (0.22–0.63)	0.93 (0.90–1)
**A7**	0.83 (0.75–0.91)	0.95 (0.72–1)	0.62 (0.50–0.85)	0.39 (0.34–0.61)	0.98 (0.92–1)	0.75 (0.66–0.85)	0.82 (0.52–1)	0.62 (0.36–0.92)	0.28 (0.22–0.58)	0.95 (0.90–1)	0.75 (0.66–0.85)	0.82 (0.52–1)	0.62 (0.36–0.91)	0.28 (0.22–0.53)	0.95 (0.91–1)
**A8**	0.84 (0.76–0.92)	0.90 (0.54–1)	0.64 (0.55–0.97)	0.40 (0.35–0.88)	0.96 (0.89–1)	0.75 (0.64–0.85)	0.82 (0.52–1)	0.63 (0.52–0.93)	0.29 (0.23–0.62)	0.95 (0.90–1)	0.75 (0.64–0.85)	0.82 (0.47–1)	0.63 (0.51–0.94)	0.29 (0.23–0.64)	0.95 (0.90–1)

**A9**	0.83 (0.72–0.93)	0.72 (0.54–0.95)	0.88 (0.63–0.96)	0.61 (0.38–0.85)	0.92 (0.88–0.98)	0.72 (0.61–0.84)	0.78 (0.43–0.95)	0.62 (0.48–0.92)	0.27 (0.23–0.57)	0.93 (0.89–0.98)	0.72 (0.61–0.84)	0.78 (0.43–0.95)	0.62 (0.48–0.92)	0.27 (0.22–0.57)	0.93 (0.89–0.98)
	**Discovery cohort (*n* = 215)**					**Replication cohort (*n* = 148)**					**Replication cohort when adding age to the model (*n* = 148)**				
**Signature**	**AUC (95% CI)**	**BPsens (95% CI)**	**BPspec (95% CI)**	**PPV (95% CI)**	**NPV (95% CI)**	**AUC (95% CI)**	**BPsens (95% CI)**	**BPspec (95% CI)**	**PPV (95% CI)**	**NPV (95% CI)**	**AUC (95% CI)**	**BPsens (95% CI)**	**BPspec (95% CI)**	**PPV (95% CI)**	**NPV (95% CI)**
**B1**	0.71 (0.61–0.81)	0.78 (0.68–0.95)	0.68 (0.45–0.8)	0.66 (0.56–0.75)	0.80 (0.73–0.93)	0.75 (0.67–0.83)	0.68 (0.48–0.93)	0.74 (0.45–0.89)	0.55 (0.43–0.71)	0.83 (0.78–0.93)	0.76 (0.68–0.84)	0.68 (0.44–0.95)	0.74 (0.41–0.93)	0.55 (0.43–0.79)	0.83 (0.77–0.95)
**B2**	0.73 (0.63–0.83)	0.85 (0.72–0.95)	0.65 (0.51–0.78)	0.65 (0.58–0.75)	0.84 (0.76–0.94)	0.76 (0.67–0.84)	0.70 (0.46–0.93)	0.71 (0.43–0.92)	0.53 (0.43–0.77)	0.83 (0.77–0.93)	0.75 (0.6–0.84)	0.74 (0.46–0.91)	0.66 (0.45–0.93)	0.50 (0.43–0.78)	0.84 (0.77–0.93)
**B3**	0.72 (0.63–0.82)	0.76 (0.68–0.93)	0.70 (0.51–0.81)	0.66 (0.57–0.76)	0.79 (0.73–0.91)	0.77 (0.69–0.85)	0.65 (0.48–0.87)	0.80 (0.57–0.93)	0.60 (0.47–0.80)	0.83 (0.78–0.91)	0.77 (0.69–0.85)	0.70 (0.46–0.82)	0.75 (0.68–0.95)	0.56 (0.50–0.83)	0.84 (0.78–0.90)
**B4**	0.74 (0.65–0.84)	0.93 (0.76–1)	0.56 (0.48–0.76)	0.62 (0.58–0.74)	0.91 (0.79–1)	0.76 (0.68–0.85)	0.51 (0.42–0.89)	0.90 (0.5–0.96)	0.70 (0.44–0.85)	0.79 (0.77–0.92)	0.77 (0.68–0.85)	0.63 (0.42–0.89)	0.78 (0.51–0.96)	0.57 (0.44–0.85)	0.82 (0.77–0.92)
**B5**	0.74 (0.65–0.84)	0.80 (0.68–0.95)	0.66 (0.48–0.81)	0.65 (0.57–0.77)	0.81 (0.74–0.95)	0.77 (0.69–0.85)	0.61 (0.42–0.95)	0.81 (0.41–0.97)	0.60 (0.43–0.88)	0.82 (0.77–0.96)	0.77 (0.69–0.85)	0.61 (0.42–0.89)	0.81 (0.53–0.97)	0.60 (0.45–0.88)	0.82 (0.77–0.92)
**B6**	0.75 (0.66–0.84)	0.82 (0.72–0.97)	0.66 (0.46–0.8)	0.66 (0.57–0.76)	0.83 (0.76–0.97)	0.76 (0.68–0.85)	0.59 (0.4–0.91)	0.81 (0.49–0.97)	0.59 (0.43–0.87)	0.81 (0.77–0.93)	0.76 (0.68–0.85)	0.59 (0.4–0.93)	0.81 (0.49–0.97)	0.59 (0.44–0.88)	0.81 (0.77–0.93)
**B7**	0.74 (0.64–0.83)	0.82 (0.74–0.95)	0.66 (0.50–0.80)	0.66 (0.58–0.76)	0.83 (0.76–0.95)	0.75 (0.66–0.83)	0.53 (0.44–0.89)	0.87 (0.53–0.94)	0.65 (0.44–0.80)	0.80 (0.77–0.92)	0.75 (0.66–0.83)	0.53 (0.44–0.89)	0.87 (0.54–0.94)	0.65 (0.44–0.80)	0.80 (0.77–0.92)
**B8**	0.74 (0.65–0.84)	0.78 (0.63–0.91)	0.68 (0.51–0.85)	0.66 (0.57–0.79)	0.80 (0.71–0.91)	0.74 (0.65–0.82)	0.70 (0.44–0.89)	0.70 (0.47–0.92)	0.52 (0.43–0.75)	0.83 (0.76–0.91)	0.74 (0.65–0.82)	0.70 (0.42–0.91)	0.69 (0.47–0.94)	0.51 (0.43–0.77)	0.83 (0.76–0.92)
**B9**	0.73 (0.64–0.83)	0.82 (0.51–0.93)	0.60 (0.50–0.90)	0.61 (0.56–0.80)	0.81 (0.69–0.90)	0.72 (0.64–0.81)	0.72 (0.42–0.87)	0.66 (0.56–0.93)	0.50 (0.43–0.79)	0.83 (0.76–0.91)	0.72 (0.64–0.81)	0.72 (0.42–0.89)	0.66 (0.55–0.93)	0.50 (0.43–0.78)	0.83 (0.76–0.91)

**B10**	0.75 (0.66–0.85)	0.72 (0.48–0.85)	0.73 (0.63–0.95)	0.68 (0.60–0.88)	0.77 (0.68–0.86)	0.70 (0.61–0.80)	0.61 (0.42–0.8)	0.77 (0.58–0.91)	0.55 (0.45–0.72)	0.81 (0.76–0.88)	0.70 (0.61–0.80)	0.61 (0.42–0.8)	0.77 (0.58–0.92)	0.55 (0.45–0.73)	0.81 (0.76–0.88)
**B11**	0.74 (0.65–0.84)	0.82 (0.44–0.91)	0.58 (0.50–0.95)	0.60 (0.56–0.88)	0.81 (0.67–0.9)	0.67 (0.58–0.76)	0.70 (0.38–0.89)	0.60 (0.45–0.91)	0.45 (0.40–0.70)	0.81 (0.75–0.91)	0.67 (0.58–0.76)	0.70 (0.38–0.89)	0.60 (0.44–0.91)	0.45 (0.40–0.70)	0.81 (0.75–0.92)
**Signature**	**Discovery cohort (*n* = 215)**					**Replication cohort (*n* = 148)**					**Replication cohort when adding age to the model (*n* = 148)**				
	**AUC (95% CI)**	**BPsens (95% CI)**	**BPspec (95% CI)**	**PPV (95% CI)**	**NPV (95% CI)**	**AUC (95% CI)**	**BPsens (95% CI)**	**BPspec (95% CI)**	**PPV (95% CI)**	**NPV (95% CI)**	**AUC (95% CI)**	**BPsens (95% CI)**	**BPspec (95% CI)**	**PPV (95% CI)**	**NPV (95% CI)**
**C1**	0.87 (0.79–0.96)	0.77 (0.68–1)	0.84 (0.53–0.97)	0.73 (0.55–0.93)	0.86 (0.83–1)	0.77 (0.61–0.92)	0.61 (0.44–0.94)	0.93 (0.62–1)	0.78 (0.47–1)	0.85 (0.79–0.96)	0.77 (0.61–0.93)	0.66 (0.44–0.94)	0.86 (0.62–1)	0.66 (0.48–1)	0.86 (0.79–0.96)
**C2**	0.93 (0.88–0.99)	0.86 (0.77–1)	0.92 (0.69–1)	0.86 (0.64–1)	0.92 (0.88–1)	0.77 (0.61–0.93)	0.72 (0.5–0.88)	0.86 (0.74–1)	0.68 (0.54–1)	0.88 (0.80–0.95)	0.77 (0.6–0.94)	0.72 (0.5–0.94)	0.86 (0.74–1)	0.68 (0.55–1)	0.88 (0.81–0.97)
**C3**	0.92 (0.86–0.99)	1 (0.86–1)	0.76 (0.69–0.97)	0.70 (0.62–0.94)	1 (0.91–1)	0.78 (0.63–0.94)	0.77 (0.44–0.94)	0.76 (0.72–1)	0.58 (0.53–1)	0.89 (0.80–0.96)	0.78 (0.62–0.94)	0.72 (0.50–0.94)	0.86 (0.74–1)	0.68 (0.56–1)	0.88 (0.81–0.97)
**C4**	0.95 (0.90–0.99)	0.95 (0.9–1)	0.87 (0.71–0.97)	0.80 (0.66–0.95)	0.97 (0.94–1)	0.78 (0.62–0.94)	0.66 (0.44–0.88)	0.90 (0.69–1)	0.75 (0.53–1)	0.86 (0.80–0.95)	0.78 (0.62–0.94)	0.66 (0.44–0.94)	0.93 (0.74–1)	0.80 (0.57–1)	0.86 (0.8–0.97)
**C5**	0.94 (0.88–0.99)	0.95 (0.86–1)	0.84 (0.71–0.97)	0.77 (0.66–0.95)	0.97 (0.92–1)	0.80 (0.65–0.95)	0.66 (0.5–0.88)	0.93 (0.72–1)	0.80 (0.55–1)	0.86 (0.81–0.95)	0.80 (0.65–0.95)	0.66 (0.50–0.94)	0.95 (0.76–1)	0.85 (0.59–1)	0.87 (0.82–0.97)
**C6**	0.95 (0.90–1)	0.90 (0.86–1)	0.92 (0.76–1)	0.86 (0.7–1)	0.94 (0.92–1)	0.78 (0.62–0.94)	0.61 (0.44–0.88)	0.97 (0.72–1)	0.91 (0.54–1)	0.85 (0.80–0.95)	0.78 (0.62–0.94)	0.66 (0.44–0.88)	0.93 (0.72–1)	0.80 (0.54–1)	0.86 (0.8–0.95)
**C7**	0.95 (0.90–1)	0.95 (0.86–1)	0.89 (0.76–1)	0.84 (0.7–1)	0.97 (0.92–1)	0.78 (0.63–0.93)	0.61 (0.44–0.88)	0.95 (0.74–1)	0.84 (0.55–1)	0.85 (0.80–0.95)	0.77 (0.61–0.94)	0.66 (0.44–0.94)	0.90 (0.72–1)	0.75 (0.53–1)	0.86 (0.8–0.96)
**C8**	0.95 (0.90–1)	0.95 (0.86–1)	0.87 (0.74–0.97)	0.80 (0.68–0.95)	0.97 (0.92–1)	0.80 (0.67–0.93)	0.61 (0.44–0.88)	0.95 (0.72–1)	0.84 (0.52–1)	0.85 (0.80–0.95)	0.79 (0.64–0.94)	0.66 (0.44–0.94)	0.90 (0.72–1)	0.75 (0.54–1)	0.86 (0.8–0.96)
**C9**	0.94	0.95 (0.81–1)	0.84	0.77	0.97 (0.9–1)	0.81	0.66 (0.5–1)	0.88	0.70	0.86 (0.81–1)	0.80	0.72 (0.55–1)	0.86	0.68	0.88 (0.82–1)

	(0.89–0.99)		(0.71–0.97)	(0.65–0.95)		(0.69–0.93)		(0.48–0.97)	(0.43–0.94)		(0.67–0.93)		(0.51–0.97)	(0.44–0.92)	
**C10**	0.93 (0.88–0.99)	1 (0.77–1)	0.74 (0.66–1)	0.68 (0.62–1)	1 (0.87–1)	0.81 (0.68–0.95)	0.66 (0.5–1)	0.95 (0.53–1)	0.85 (0.45–1)	0.87 (0.82–1)	0.81 (0.67–0.94)	0.61 (0.44–0.94)	0.95 (0.53–1)	0.84 (0.45–1)	0.85 (0.8–0.97)
**C11**	0.93 (0.86–0.99)	0.95 (0.72–1)	0.79 (0.71–1)	0.72 (0.64–1)	0.96 (0.86–1)	0.78 (0.63–0.93)	0.55 (0.38–0.88)	0.97 (0.72–1)	0.90 (0.51–1)	0.84 (0.79–0.95)	0.77 (0.61–0.93)	0.55 (0.38–0.88)	1 (0.72–1)	1 (0.53–1)	0.84 (0.79–0.94)
**C12**	0.90 (0.82–0.99)	0.86 (0.68–1)	0.84 (0.71–1)	0.76 (0.64–1)	0.91 (0.82–1)	0.80 (0.67–0.93)	0.77 (0.44–1)	0.74 (0.44–1)	0.56 (0.42–1)	0.88 (0.80–1)	0.79 (0.65–0.92)	0.72 (0.44–0.94)	0.83 (0.62–1)	0.65 (0.47–1)	0.87 (0.8–0.97)

Supplementary Table S7: Statistically significant tests for predictive comparisons in treatment groups

a) First statistical approach

Treatment	Protein	Test type	LOD check*	Median 1st group (survival ≤180 days)	Median 2nd group (survival >180 days)	Log2 fold change	*P* value	Adjusted *P* value
Gemcitabine	IL-8	Wilcoxon	No flag	0.106	-0.42	0.77	5.9×10^{-08}	2.7×10^{-06}
Gemcitabine	CCL20	Wilcoxon	No flag	0.260	-0.27	0.77	6.0×10^{-06}	1.4×10^{-04}
Gemcitabine	**IL-6**	Wilcoxon	No flag	0.347	-0.39	0.60	1.1×10^{-05}	2.2×10^{-04}
Gemcitabine	HGF	Wilcoxon	No flag	0.058	-0.43	0.58	1.6×10^{-05}	2.9×10^{-04}
Gemcitabine	CSF-1	*t* test	No flag	0.378	-0.13	0.51	8.9×10^{-05}	1.1×10^{-03}
Gemcitabine	**ANGPT2**	*t* test	No flag	0.103	-0.24	0.57	1.2×10^{-04}	1.3×10^{-03}
Gemcitabine	CASP-8	Wilcoxon	No flag	0.266	-0.23	0.42	3.5×10^{-04}	3.0×10^{-03}
Gemcitabine	ADGRG1	Wilcoxon	No flag	0.158	-0.39	0.50	3.6×10^{-04}	3.0×10^{-03}
Gemcitabine	CA19-9	*t* test	No flag	0.389	-0.15	0.48	5.7×10^{-04}	4.3×10^{-03}
Gemcitabine	**TNFRSF12A**	Wilcoxon	No flag	0.169	-0.19	0.53	6.8×10^{-04}	4.9×10^{-03}
Gemcitabine	**IL-10**	Wilcoxon	No flag	0.011	-0.31	0.37	1.3×10^{-03}	7.9×10^{-03}
Gemcitabine	MCP-3	*t* test	No flag	0.227	-0.24	0.43	1.4×10^{-03}	8.5×10^{-03}
Gemcitabine	NOS3	Wilcoxon	No flag	0.043	-0.26	0.55	2.6×10^{-03}	1.4×10^{-02}
Gemcitabine	CAIX	Wilcoxon	No flag	0.071	-0.29	0.60	2.8×10^{-03}	1.5×10^{-02}
Gemcitabine	MMP12	*t* test	No flag	0.126	-0.12	0.36	1.3×10^{-02}	5.0×10^{-02}
Gemcitabine + nab-Paclitaxel	CSF-1	*t* test	No flag	0.692	-0.72	1.12	2.4×10^{-06}	6.8×10^{-05}
Gemcitabine + nab-Paclitaxel	MCP-3	*t* test	No flag	0.702	-0.54	1.01	1.9×10^{-05}	3.3×10^{-04}
Gemcitabine + nab-Paclitaxel	CAIX	Wilcoxon	No flag	0.339	-0.44	0.96	6.0×10^{-05}	9.5×10^{-04}
Gemcitabine + nab-Paclitaxel	CX3CL1	Wilcoxon	No flag	0.456	-0.34	0.68	7.4×10^{-05}	1.0×10^{-03}
Gemcitabine + nab-Paclitaxel	HGF	Wilcoxon	No flag	0.374	-0.32	0.74	7.7×10^{-05}	1.0×10^{-03}
Gemcitabine + nab-Paclitaxel	**IL-6**	Wilcoxon	No flag	0.528	-0.58	0.94	9.6×10^{-05}	1.1×10^{-03}
Gemcitabine + nab-Paclitaxel	**TNFRSF12A**	Wilcoxon	No flag	0.604	-0.34	0.95	1.1×10^{-04}	1.3×10^{-03}
Gemcitabine + nab-Paclitaxel	IL-8	Wilcoxon	No flag	0.430	-0.39	0.85	1.1×10^{-04}	1.3×10^{-03}
Gemcitabine + nab-Paclitaxel	PGF	Wilcoxon	No flag	0.459	-0.29	0.70	3.2×10^{-04}	2.9×10^{-03}
Gemcitabine + nab-Paclitaxel	CCL20	Wilcoxon	No flag	0.400	-0.52	0.78	3.6×10^{-04}	3.0×10^{-03}
Gemcitabine + nab-Paclitaxel	ADGRG1	Wilcoxon	No flag	0.321	-0.54	0.67	3.9×10^{-04}	3.2×10^{-03}
Gemcitabine + nab-Paclitaxel	PD-L2	Wilcoxon	No flag	0.315	-0.33	0.67	8.0×10^{-04}	5.5×10^{-03}
Gemcitabine + nab-Paclitaxel	NOS3	*t* test	No flag	0.625	-0.34	0.77	8.3×10^{-04}	5.5×10^{-03}
Gemcitabine + nab-Paclitaxel	ADA	*t* test	No flag	0.468	-0.23	0.70	8.7×10^{-04}	5.7×10^{-03}
Gemcitabine + nab-Paclitaxel	TIE2	*t* test	No flag	0.558	-0.37	0.83	9.9×10^{-04}	6.4×10^{-03}

Gemcitabine + nab-Paclitaxel	TNFRSF21	*t* test	No flag	0.631	-0.33	0.74	$1.3x10^{-03}$	$7.9x10^{-03}$
Gemcitabine + nab-Paclitaxel	**ANGPT2**	Wilcoxon	No flag	0.602	-0.42	0.69	$1.5x10^{-03}$	$8.9x10^{-03}$
Gemcitabine + nab-Paclitaxel	IL-12RB1	*t* test	No flag	0.300	-0.50	0.69	$2.3x10^{-03}$	$1.3x10^{-02}$
Gemcitabine + nab-Paclitaxel	TNFRSF9	*t* test	No flag	0.264	-0.25	0.64	$4.2x10^{-03}$	$2.1x10^{-02}$
Gemcitabine + nab-Paclitaxel	CD4	*t* test	No flag	0.474	-0.24	0.66	$4.7x10^{-03}$	$2.3x10^{-02}$
Gemcitabine + nab-Paclitaxel	**IL-10**	Wilcoxon	No flag	0.294	-0.54	0.72	$4.7x10^{-03}$	$2.3x10^{-02}$
Gemcitabine + nab-Paclitaxel	CD40	*t* test	No flag	0.283	-0.43	0.58	$4.8x10^{-03}$	$2.3x10^{-02}$
Gemcitabine + nab-Paclitaxel	PD-L1	*t* test	No flag	0.377	-0.12	0.57	$6.7x10^{-03}$	$3.0x10^{-02}$
Gemcitabine + nab-Paclitaxel	TNFRSF4	*t* test	No flag	0.462	-0.26	0.69	$7.0x10^{-03}$	$3.1x10^{-02}$
Gemcitabine + nab-Paclitaxel	VEGFA	*t* test	No flag	0.155	-0.30	0.59	$8.4x10^{-03}$	$3.6x10^{-02}$
Gemcitabine + nab-Paclitaxel	MMP12	*t* test	No flag	0.487	-0.22	0.66	$9.0x10^{-03}$	$3.8x10^{-02}$
Gemcitabine + nab-Paclitaxel	Gal-9	*t* test	No flag	0.459	-0.16	0.62	$1.0x10^{-02}$	$4.1x10^{-02}$
Gemcitabine + nab-Paclitaxel	MCP-1	*t* test	No flag	0.372	-0.32	0.69	$1.0x10^{-02}$	$4.1x10^{-02}$
Gemcitabine + nab-Paclitaxel	DCN	*t* test	No flag	0.213	-0.29	0.55	$1.2x10^{-02}$	$4.9x10^{-02}$
FOLFIRINOX	CCL23	*t* test	No flag	0.714	-0.16	0.74	$1.9x10^{-03}$	$1.1x10^{-02}$
FOLFIRINOX	**ANGPT2**	*t* test	No flag	0.370	-0.29	0.74	$1.9x10^{-03}$	$1.1x10^{-02}$
FOLFIRINOX	**IL-6**	*t* test	No flag	0.498	-0.27	0.89	$3.3x10^{-03}$	$1.7x10^{-02}$
FOLFIRINOX	**TNFRSF12A**	Wilcoxon	No flag	0.216	-0.59	0.57	$3.9x10^{-03}$	$2.0x10^{-02}$
FOLFIRINOX	TNFSF14	Wilcoxon	No flag	0.721	$-3.8x10^{-03}$	0.66	$5.7x10^{-03}$	$2.6x10^{-02}$
FOLFIRINOX	**IL-10**	Wilcoxon	No flag	0.088	-0.25	0.51	$1.0x10^{-02}$	$4.1x10^{-02}$
All treatments combined	IL-8	Wilcoxon	No flag	0.222	-0.39	0.68	$2.5x10^{-11}$	$8.3x10^{-09}$
All treatments combined	**IL-6**	Wilcoxon	No flag	0.426	-0.39	0.71	$4.5x10^{-11}$	$8.3x10^{-09}$
All treatments combined	CSF-1	*t* test	No flag	0.458	-0.21	0.67	$1.2x10^{-10}$	$1.5x10^{-08}$
All treatments combined	TNFRSF12A	Wilcoxon	No flag	0.290	-0.33	0.68	$1.3x10^{-09}$	$1.2x10^{-07}$
All treatments combined	**ANGPT2**	*t* test	No flag	0.238	-0.32	0.64	$8.0x10^{-09}$	$5.1x10^{-07}$
All treatments combined	HGF	Wilcoxon	No flag	0.153	-0.37	0.56	$9.3x10^{-09}$	$5.1x10^{-07}$
All treatments combined	CCL20	Wilcoxon	No flag	0.216	-0.34	0.64	$9.6x10^{-09}$	$5.1x10^{-07}$
All treatments combined	NOS3	Wilcoxon	No flag	0.130	-0.32	0.60	$2.0x10^{-07}$	$8.2x10^{-06}$
All treatments combined	MCP-3	Wilcoxon	No flag	0.261	-0.26	0.51	$3.8x10^{-07}$	$1.4x10^{-05}$
All treatments combined	**IL-10**	Wilcoxon	No flag	0.105	-0.32	0.49	$7.0x10^{-07}$	$2.4x10^{-05}$
All treatments combined	ADGRG1	Wilcoxon	No flag	0.154	-0.43	0.45	$1.2x10^{-06}$	$3.8x10^{-05}$
All treatments combined	CAIX	Wilcoxon	No flag	0.087	-0.35	0.63	$2.6x10^{-06}$	$6.8x10^{-05}$
All treatments combined	PGF	Wilcoxon	No flag	0.197	-0.27	0.48	$4.2x10^{-06}$	$1.0x10^{-04}$
All treatments combined	CX3CL1	Wilcoxon	No flag	0.243	-0.21	0.48	$7.8x10^{-06}$	$1.7x10^{-04}$
All treatments combined	TIE2	Wilcoxon	No flag	0.144	-0.23	0.46	$1.1x10^{-05}$	$2.2x10^{-04}$
All treatments combined	MMP12	Wilcoxon	No flag	0.237	-0.19	0.42	$5.4x10^{-05}$	$9.1x10^{-04}$
All treatments combined	CD40	Wilcoxon	No flag	0.220	-0.30	0.42	$6.1x10^{-05}$	$9.5x10^{-04}$
All treatments combined	CASP-8	Wilcoxon	No flag	0.274	-0.21	0.40	$7.0x10^{-05}$	$1.0x10^{-03}$
All treatments combined	TNFRSF21	*t* test	No flag	0.372	-0.20	0.44	$7.4x10^{-05}$	$1.0x10^{-03}$
All treatments combined	*CCL3*	Wilcoxon	No flag	0.139	-0.24	0.43	$8.6x10^{-05}$	$1.1x10^{-03}$
All treatments combined	TNFRSF4	*t* test	No flag	0.285	-0.17	0.42	$1.5x10^{-04}$	$1.6x10^{-03}$

All treatments combined	*MIC-A/B*	Wilcoxon	No flag	0.414	0.16	0.24	1.6x10^{-04}	1.6x10^{-03}
All treatments combined	ADA	Wilcoxon	No flag	0.218	-0.24	0.36	1.7x10^{-04}	1.7x10^{-03}
All treatments combined	DCN	Wilcoxon	No flag	0.153	-0.21	0.43	1.9x10^{-04}	1.8x10^{-03}
All treatments combined	CA19-9	Wilcoxon	No flag	0.362	-0.14	0.37	2.0x10^{-04}	1.8x10^{-03}
All treatments combined	VEGFA	Wilcoxon	No flag	0.205	-0.19	0.39	2.0x10^{-04}	1.8x10^{-03}
All treatments combined	PD-L1	Wilcoxon	No flag	0.232	-0.14	0.36	2.4x10^{-04}	2.2x10^{-03}
All treatments combined	IL12RB1	Wilcoxon	No flag	0.205	-0.18	0.40	5.1x10^{-04}	4.0x10^{-03}
All treatments combined	CCL23	*t* test	No flag	0.311	-0.20	0.39	5.4x10^{-04}	4.2x10^{-03}
All treatments combined	*CXCL13*	Wilcoxon	No flag	0.116	-0.20	0.33	6.5x10^{-04}	4.9x10^{-03}
All treatments combined	CD4	*t* test	No flag	0.132	-0.10	0.38	7.4x10^{-04}	5.3x10^{-03}
All treatments combined	Gal-9	Wilcoxon	No flag	0.218	-0.15	0.39	7.7x10^{-04}	5.4x10^{-03}
All treatments combined	*TRAIL*	Wilcoxon	No flag	-0.169	0.14	-0.39	8.2x10^{-04}	5.5x10^{-03}
All treatments combined	TNFSF14	*t* test	No flag	0.213	-4.1x10^{-02}	0.35	1.1x10^{-03}	7.2x10^{-03}
All treatments combined	*CXCL1*	Wilcoxon	No flag	0.257	-6.0x10^{-02}	0.35	1.6x10^{-03}	9.1x10^{-03}
All treatments combined	*GZMH*	Wilcoxon	No flag	0.158	-0.20	0.32	2.7x10^{-03}	1.5x10^{-02}
All treatments combined	*HO-1*	Wilcoxon	No flag	0.223	-8.3x10^{-02}	0.30	2.9x10^{-03}	1.5x10^{-02}
All treatments combined	*MMP7*	Wilcoxon	No flag	0.318	5.3x10^{-02}	0.32	3.0x10^{-03}	1.5x10^{-02}
All treatments combined	*KLRD1*	*t* test	No flag	0.242	-0.18	0.31	4.9x10^{-03}	2.3x10^{-02}
All treatments combined	*TNF*	Wilcoxon	Flag	-0.184	-0.29	0.22	6.0x10^{-03}	2.8x10^{-02}
All treatments combined	PD-L2	Wilcoxon	No flag	0.187	-0.12	0.23	6.1x10^{-03}	2.8x10^{-02}
All treatments combined	*CCL4*	Wilcoxon	No flag	0.185	-0.20	0.24	7.8x10^{-03}	3.4x10^{-02}
All treatments combined	MCP-1	*t* test	No flag	0.128	-3.6x10^{-02}	0.29	9.2x10^{-03}	3.9x10^{-02}
All treatments combined	TNFRSF9	*t* test	No flag	0.216	-0.13	0.28	9.6x10^{-03}	4.0x10^{-02}
All treatments combined	*IL-5*	Wilcoxon	Flag	-0.196	-0.37	0.28	9.8x10^{-03}	4.1x10^{-02}

*LOD-check: We checked to see whether the differential expression of the medians of the two investigated groups was below the limit of detection (LOD) for that given protein. If the medians of both groups were below LOD, the test would be marked as "Flag", and if the median of one of the groups or none of groups was below LOD, this would be marked as "No flag".
Proteins in **bold** are the proteins present in all four comparisons. Proteins in *italic and red* are the proteins unique to the comparison in question.
Abbreviations: Wilcoxon = Wilcoxon Rank Sum Test

b) Second statistical approach

**Treatment**	**Protein**	**HR**	**95% CI**	***P* value (log-rank)**
Gemcitabine	**IL-6**	2.23	1.65–3.01	0
Gemcitabine	IL-8	2.2	1.63–2.97	0
Gemcitabine	CASP-8	1.91	1.41–2.58	0.00003
Gemcitabine	**HGF**	1.77	1.32–2.38	0.00013
Gemcitabine	**MCP-3**	1.7	1.26–2.29	0.00049
Gemcitabine	**TNFRSF12A**	1.63	1.21–2.18	0.00119
Gemcitabine	CCL3	1.61	1.20–2.18	0.00171
Gemcitabine	MMP12	1.56	1.16–2.09	0.00329
Gemcitabine	**CSF-1**	1.53	1.14–2.05	0.00452
Gemcitabine	CXCL13	1.52	1.13–2.05	0.00549
Gemcitabine	CXCL1	1.52	1.13–2.04	0.00587
Gemcitabine	*IL-2*	1.45	1.08–1.96	0.01363
Gemcitabine	GZMH	1.45	1.08–1.94	0.01374
Gemcitabine	*TWEAK*	0.69	0.51–0.93	0.01518
Gemcitabine	*IL-5*	1.41	1.05–1.89	0.02134
Gemcitabine	**ANGPT2**	1.41	1.05–1.89	0.02407
Gemcitabine	IL-10	1.4	1.04–1.88	0.02539
Gemcitabine	TNF	1.39	1.04–1.86	0.0271
Gemcitabine	CCL4	1.39	1.03–1.87	0.02927
Gemcitabine	CCL20	1.39	1.03–1.86	0.0293
Gemcitabine	*ARG1*	1.37	1.02–1.85	0.03602
Gemcitabine	PD-L1	1.36	1.02–1.83	0.03803
Gemcitabine	TNFSF14	1.36	1.01–1.83	0.04088
Gemcitabine + nab-Paclitaxel	**CSF-1**	2.97	1.86–4.74	0.00001
Gemcitabine + nab-Paclitaxel	IL-8	2.5	1.58–3.97	0.00009
Gemcitabine + nab-Paclitaxel	**MCP-3**	2.48	1.56–3.95	0.00012
Gemcitabine + nab-Paclitaxel	CCL20	2.16	1.36–3.43	0.00105
Gemcitabine + nab-Paclitaxel	TIE2	2.14	1.34–3.40	0.00136
Gemcitabine + nab-Paclitaxel	CX3CL1	2.08	1.32–3.25	0.00144
Gemcitabine + nab-Paclitaxel	MMP7	2.05	1.30–3.23	0.00204
Gemcitabine + nab-Paclitaxel	TNFRSF21	2.04	1.29–3.21	0.00212
Gemcitabine + nab-Paclitaxel	**HGF**	1.97	1.26–3.10	0.00322
Gemcitabine + nab-Paclitaxel	ADGRG1	1.97	1.25–3.11	0.0036
Gemcitabine + nab-Paclitaxel	ADA	1.99	1.25–3.17	0.0037
Gemcitabine + nab-Paclitaxel	VEGFA	1.9	1.21–3.01	0.00564
Gemcitabine + nab-Paclitaxel	**TNFRSF12A**	1.89	1.20–2.96	0.00592
Gemcitabine + nab-Paclitaxel	CD40	1.89	1.19–3.00	0.00655
Gemcitabine + nab-Paclitaxel	**IL-6**	1.85	1.17–2.91	0.00798
Gemcitabine + nab-Paclitaxel	**ANGPT2**	1.8	1.15–2.83	0.01029
Gemcitabine + nab-Paclitaxel	NOS3	1.7	1.09–2.66	0.02015
Gemcitabine + nab-Paclitaxel	TRAIL	0.59	0.38–0.92	0.02016
Gemcitabine + nab-Paclitaxel	CXCL1	1.68	1.07–2.65	0.024
Gemcitabine + nab-Paclitaxel	DCN	1.63	1.04–2.55	0.03395
Gemcitabine + nab-Paclitaxel	*PTN*	1.62	1.02–2.55	0.0393
Gemcitabine + nab-Paclitaxel	*Gal-1*	1.6	1.02–2.51	0.04137
Gemcitabine + nab-Paclitaxel	MMP12	1.59	1.02–2.48	0.04258
Gemcitabine + nab-Paclitaxel	PD-L2	1.57	1.00–2.46	0.04966
mFOLFIRINOX	**IL-6**	2.54	1.67–3.86	0.00001
mFOLFIRINOX	TIE2	2.39	1.56–3.69	0.00007
mFOLFIRINOX	**ANGPT2**	2.08	1.38–3.15	0.0005
mFOLFIRINOX	**CSF-1**	2.09	1.37–3.19	0.00062

mFOLFIRINOX	PD-L1	1.99	1.31–3.03	0.0013
mFOLFIRINOX	CX3CL1	1.85	1.22–2.81	0.00355
mFOLFIRINOX	TNFRSF21	1.77	1.18–2.65	0.00599
mFOLFIRINOX	*MCP-4*	0.56	0.37–0.86	0.00731
mFOLFIRINOX	**TNFRSF12A**	1.69	1.13–2.54	0.01057
mFOLFIRINOX	FGF2	1.7	1.13–2.57	0.01144
mFOLFIRINOX	*VEGFC*	0.59	0.39–0.89	0.01307
mFOLFIRINOX	DCN	1.67	1.11–2.52	0.01375
mFOLFIRINOX	CCL23	1.64	1.09–2.47	0.0167
mFOLFIRINOX	*CD5*	1.62	1.08–2.45	0.02078
mFOLFIRINOX	CRTAM	1.62	1.07–2.45	0.02132
mFOLFIRINOX	*IL-13*	1.61	1.07–2.43	0.02284
mFOLFIRINOX	VEGFA	1.58	1.05–2.39	0.02783
mFOLFIRINOX	**MCP-3**	1.58	1.05–2.39	0.02815
mFOLFIRINOX	CD40	1.57	1.05–2.36	0.02879
mFOLFIRINOX	**HGF**	1.52	1.02–2.28	0.04201
mFOLFIRINOX	HO-1	1.52	1.01–2.28	0.04327
mFOLFIRINOX	CXCL13	1.5	1.00–2.25	0.04812
mFOLFIRINOX	PGF	1.5	1.00–2.24	0.04884
All treatments combined	**ANGPT2**	1.68	1.36–2.07	0
All treatments combined	**CSF-1**	1.85	1.50–2.28	0
All treatments combined	**IL-6**	2.16	1.74–2.67	0
All treatments combined	IL-8	1.78	1.44–2.19	0
All treatments combined	**MCP-3**	1.81	1.46–2.23	0
All treatments combined	**TNFRSF12A**	1.67	1.35–2.05	0
All treatments combined	**HGF**	1.56	1.27–1.92	0.00003
All treatments combined	TIE2	1.56	1.26–1.92	0.00003
All treatments combined	TNFRSF21	1.55	1.25–1.91	0.00005
All treatments combined	PD-L1	1.53	1.24–1.88	0.00007
All treatments combined	PGF	1.53	1.24–1.88	0.00007
All treatments combined	CASP-8	1.52	1.23–1.87	0.00008
All treatments combined	CCL23	1.52	1.23–1.87	0.00009
All treatments combined	CCL3	1.52	1.23–1.88	0.00009
All treatments combined	CXCL1	1.5	1.21–1.85	0.00016
All treatments combined	MMP12	1.47	1.20–1.82	0.00026
All treatments combined	CX3CL1	1.47	1.19–1.81	0.0003
All treatments combined	CXCL13	1.44	1.17–1.77	0.00067
All treatments combined	VEGFA	1.44	1.17–1.77	0.00068
All treatments combined	GZMH	1.42	1.15–1.75	0.00107
All treatments combined	IL-10	1.4	1.13–1.72	0.00162
All treatments combined	DCN	1.4	1.13–1.72	0.00171
All treatments combined	*TNFRSF4*	1.39	1.13–1.72	0.0019
All treatments combined	CD40	1.39	1.13–1.71	0.00192
All treatments combined	NOS3	1.39	1.13–1.71	0.00195
All treatments combined	*TNFRSF9*	1.38	1.12–1.70	0.00243
All treatments combined	CCL20	1.37	1.11–1.69	0.00297
All treatments combined	*MIC-A/B*	1.36	1.10–1.68	0.00378
All treatments combined	MMP7	1.34	1.09–1.65	0.00613
All treatments combined	CRTAM	1.33	1.08–1.64	0.00686
All treatments combined	*LAP-TGF-beta-1*	1.32	1.07–1.63	0.00826
All treatments combined	FGF2	1.32	1.07–1.63	0.00989
All treatments combined	*CXCL11*	1.31	1.06–1.61	0.0108
All treatments combined	TNF	1.29	1.04–1.58	0.01812
All treatments combined	CCL4	1.28	1.04–1.58	0.01882
All treatments combined	TNFSF14	1.28	1.04–1.58	0.01977

All treatments combined	HO-1	1.27	1.03–1.57	0.02259
All treatments combined	ADA	1.26	1.03–1.56	0.0283
All treatments combined	*CXCL10*	1.25	1.02–1.54	0.03309
All treatments combined	ADGRG1	1.25	1.02–1.54	0.03491
All treatments combined	PD-L2	1.24	1.01–1.53	0.03906
All treatments combined	TRAIL	0.81	0.65–0.99	0.04181

Proteins in **bold** are the proteins present in all four comparisons.

Proteins in *italics and red* are the proteins unique to the comparison in question.

Supplementary Table S8: Proteins with statistically significant interaction effects in the longitudinal analyses

a) All statistically significant interaction effects for changes in NPX values between two samples (baseline and visit 2) and 180-day survival

Effect name	Protein	Estimate	StdError	df	*t* value	*P* value
timePointvisit2 : ≤180 days	CX3CL1	0.31	0.112	196	2.7	6.7×10^{-03}
timePointvisit2 : ≤180 days	IL-33	-0.41	0.165	196	-2.5	0.013
timePointvisit2 : ≤180 days	GZMA	-0.25	0.122	197	-2.0	0.042
timePointvisit2 : ≤180 days	CSF-1*	-0.23	0.117	194	-2.0	0.046

b) All statistically significant interaction effects for changes in NPX values between two samples (baseline and visit 3) and 180-day survival

Effect name	Protein	Estimate	StdError	df	*t* value	*P* value
timePointvisit3 : ≤180 days	TNFRSF12A*^	0.61	0.188	170	3.2	1.4×10^{-03}
timePointvisit3 : ≤180 days	PD-L2	0.67	0.209	168	3.2	1.6×10^{-03}
timePointvisit3 : ≤180 days	IL-7	0.68	0.216	169	3.1	2.0×10^{-03}
timePointvisit3 : ≤180 days	LAP-TGF-beta-1	0.73	0.232	166	3.1	2.1×10^{-03}
timePointvisit3 : ≤180 days	CXCL1	0.72	0.237	166	3.0	2.7×10^{-03}
timePointvisit3 : ≤180 days	CXCL12	0.71	0.233	170	3.0	2.8×10^{-03}
timePointvisit3 : ≤180 days	DCN	0.60	0.208	169	2.9	4.3×10^{-03}
timePointvisit3 : ≤180 days	PD-L1	0.55	0.193	170	2.9	4.7×10^{-03}
timePointvisit3 : ≤180 days	CXCL11	0.58	0.223	170	2.6	9.7×10^{-03}
timePointvisit3 : ≤180 days	ADA	0.53	0.203	168	2.6	9.7×10^{-03}
timePointvisit3 : ≤180 days	CX3CL1	0.56	0.220	169	2.6	0.011
timePointvisit3 : ≤180 days	IL-8	0.54	0.218	167	2.5	0.014
timePointvisit3 : ≤180 days	IL-33	0.65	0.261	337	2.5	0.014
timePointvisit3 : ≤180 days	TNFRSF21	0.57	0.230	169	2.5	0.015
timePointvisit3 : ≤180 days	VEGFC	0.51	0.211	170	2.4	0.018
timePointvisit3 : ≤180 days	IL-6*^	0.48	0.207	169	2.3	0.021
timePointvisit3 : ≤180 days	CD27	0.41	0.176	170	2.3	0.021
timePointvisit3 : ≤180 days	MIC-A/B	0.12	0.053	170	2.3	0.022
timePointvisit3 : ≤180 days	VEGFA	0.44	0.197	169	2.2	0.028
timePointvisit3 : ≤180 days	MMP7	0.39	0.179	169	2.2	0.031

timePointvisit3 : ≤180 days	KLRD1	0.30	0.150	170	2.0	0.046
timePointvisit3 : ≤180 days	IL-4	0.36	0.184	169	2.0	0.049

c) All statistically significant interaction effects for changes in NPX values between two samples (baseline and visit 3) and 1 year survival

**Effect name**	**Protein**	**Estimate**	**StdError**	**df**	***t* value**	***P* value**
timePointvisit3 : ≤1year	MMP7	0.37	0.124	170	3.0	$3.1{\times}10^{-03}$
timePointvisit3 : ≤1year	PTN	0.52	0.174	159	3.0	$3.4{\times}10^{-03}$
timePointvisit3 : ≤1year	ARG1	0.48	0.171	168	2.8	$5.6{\times}10^{-03}$
timePointvisit3 : ≤1year	DCN	0.39	0.147	169	2.7	$8.1{\times}10^{-03}$
timePointvisit3 : ≤1year	HGF	0.55	0.208	171	2.7	$8.6{\times}10^{-03}$
timePointvisit3 : ≤1year	ADGRG1	0.20	0.077	170	2.6	0.011
timePointvisit3 : ≤1year	CD40	0.39	0.152	170	2.5	0.012
timePointvisit3 : ≤1year	CXCL10	0.41	0.164	171	2.5	0.013
timePointvisit3 : ≤1year	CCL19	0.33	0.134	169	2.4	0.016
timePointvisit3 : ≤1year	TWEAK*	0.51	0.212	171	2.4	0.017
timePointvisit3 : ≤1year	TNFRSF12A*^	0.31	0.134	171	2.3	0.024
timePointvisit3 : ≤1year	ADA	0.32	0.144	168	2.2	0.026
timePointvisit3 : ≤1year	CRTAM	0.22	0.107	171	2.1	0.039

Red marks proteins significant with regard to both a) baseline to visit 2 samples and 180-day survival, and b) baseline to visit 3 samples and 180-day survival. Yellow marks proteins significant with regard to both b) baseline to visit 3 samples and 180-day survival, and c) baseline to visit 3 samples and 1-year survival.

Visit 2 = before 2nd chemotherapy cycle, Visit 3 = time of first CT evaluation.

* Also in prognostic Index I.

^ Also in prognostic Index II.

Supplementary Table S9:

a) Comparison of protein levels at baseline vs. OS in univariate and multivariate analyses (including age, stage, baseline PS, baseline CA19-9, and type of palliative chemotherapy). Only proteins with statistically significant (*P* < 0.05) univariate analyses are shown.

	Univariate analysis			Multivariate analysis		
**Protein**	**HR**	**95% CI**	***P* value**	**HR**	**95% CI**	***P* value**
ADA	1.26	1.03–1.56	0.0283	1.20	0.97–1.50	0.09614
ADGRG1	1.25	1.02–1.54	0.03491	1.14	0.92–1.42	0.22589
ANGPT2	1.68	1.36–2.07	<0.0001	1.55	1.24–1.94	$1x10^{-04}$
CASP-8	1.52	1.23–1.87	0.00008	1.52	1.21–1.90	0.00031
CCL20	1.37	1.11–1.69	0.00297	1.29	1.04–1.60	0.02285
CCL23	1.52	1.23–1.87	0.00009	1.51	1.22–1.88	0.00019
CCL3	1.52	1.23–1.88	0.00009	1.48	1.18–1.85	0.00059
CCL4	1.28	1.04–1.58	0.01882	1.23	0.98–1.53	0.06948
CD40	1.39	1.13–1.71	0.00192	1.37	1.10–1.70	0.00455
CRTAM	1.33	1.08–1.64	0.00686	1.26	1.01–1.56	0.03916
**CSF-1**	**1.85**	**1.50–2.28**	**<0.0001**	**1.79**	**1.44–2.24**	**<0.0001**
CX3CL1	1.47	1.19–1.81	0.0003	1.21	0.97–1.52	0.0937
CXCL1	1.50	1.21–1.85	0.00016	1.40	1.12–1.75	0.00299
CXCL10	1.25	1.02–1.54	0.03309	1.24	0.99–1.54	0.05903
CXCL11	1.31	1.06–1.61	0.0108	1.21	0.97–1.50	0.09315
**CXCL13**	**1.44**	**1.17–1.77**	**0.00067**	**1.30**	**1.04–1.62**	**0.02337**
DCN	1.40	1.13–1.72	0.00171	1.39	1.11–1.73	0.00346
FGF2	1.32	1.07–1.63	0.00989	1.21	0.97–1.51	0.09343
GZMH	1.42	1.15–1.75	0.00107	1.35	1.08–1.68	0.00736
HGF	1.56	1.27–1.92	0.00003	1.43	1.14–1.79	0.00172
HO-1	1.27	1.03–1.57	0.02259	1.08	0.86–1.35	0.50974
IL-10	1.4	1.13–1.72	0.00162	1.24	0.99–1.54	0.06301

**IL-6**	**2.16**	**1.74–2.67**	**<0.0001**	**2.08**	**1.66–2.61**	**<0.0001**
IL-8	1.78	1.44–2.19	<0.0001	1.69	1.34–2.12	<0.0001
LAP-TGF-beta-1	1.32	1.07–1.63	0.00826	1.14	0.90–1.43	0.27327
MCP-3	1.81	1.46–2.23	<0.0001	1.57	1.25–1.97	0.00011
MIC-A/B	1.36	1.10–1.68	0.00378	1.19	0.96–1.49	0.11502
MMP12	1.47	1.20–1.82	0.00026	1.30	1.04–1.63	0.02074
MMP7	1.34	1.09–1.65	0.00613	1.32	1.06–1.65	0.01302
NOS3	1.39	1.13–1.71	0.00195	1.23	0.98–1.54	0.0711
PD-L1	1.53	1.24–1.88	0.00007	1.35	1.08–1.69	0.00813
PD-L2	1.24	1.01–1.53	0.03906	1.19	0.95–1.48	0.12885
PGF	1.53	1.24–1.88	0.00007	1.45	1.16–1.81	0.00103
TIE2	1.56	1.26–1.92	0.00003	1.41	1.13–1.76	0.00237
TNF	1.29	1.04–1.58	0.01812	1.24	1.00–1.54	0.04945
**TNFRSF12A**	**1.67**	**1.35–2.05**	**<0.0001**	**1.57**	**1.26–1.96**	**<0.0001**
TNFRSF21	1.55	1.25–1.91	0.00005	1.42	1.14–1.77	0.00191
TNFRSF4	1.39	1.13–1.72	0.0019	1.24	0.99–1.55	0.05978
TNFRSF9	1.38	1.12–1.70	0.00243	1.17	0.93–1.46	0.17238
TNFSF14	1.28	1.04–1.58	0.01977	1.20	0.96–1.51	0.10684
TRAIL	0.81	0.65–0.99	0.04181	0.81	0.65–1.00	0.05485
VEGFA	1.44	1.17–1.77	0.00068	1.25	0.99–1.56	0.05798

b) Comparison of protein levels at second treatment cycle vs. OS in univariate and multivariate analyses (including age, stage, baseline PS, baseline CA19-9, and type of palliative chemotherapy). Only proteins with statistically significant (*P* < 0.05) univariate analyses are shown.

Protein	Univariate analysis			Multivariate analysis		
	**HR**	**95% CI**	***P* value**	**HR**	**95% CI**	***P* value**
ADA	1.64	1.23–2.19	0.0007	1.55	1.15–2.09	0.00396
ADGRG1	1.50	1.13–1.99	0.0052	1.38	1.01–1.88	0.04443
ANGPT2	1.73	1.30–2.31	0.00017	1.76	1.28–2.42	0.00049
CCL20	1.51	1.14–2.01	0.00397	1.42	1.06–1.91	0.01874

CCL23	1.67	1.26–2.23	0.00042	1.92	1.42–2.60	<0.0001
CD27	1.41	1.07–1.87	0.01634	1.32	0.97–1.79	0.0745
CD4	1.57	1.18–2.09	0.00211	1.50	1.11–2.02	0.00775
CD40	1.60	1.20–2.12	0.00133	1.44	1.06–1.94	0.01964
**CSF-1**	**1.33**	**1.00–1.77**	**0.04644**	**1.35**	**1.00–1.81**	**0.05019**
CX3CL1	1.68	1.26–2.22	0.00034	1.57	1.16–2.11	0.00309
CXCL11	1.53	1.15–2.04	0.00333	1.32	0.96–1.8	0.08905
CXCL12	1.47	1.1–1.96	0.00816	1.15	0.84–1.57	0.3917
**CXCL13**	**1.42**	**1.07–1.89**	**0.01477**	**1.33**	**0.98–1.79**	**0.06633**
DCN	1.41	1.07–1.88	0.01661	1.35	1.00–1.82	0.04789
GZMH	1.42	1.07–1.88	0.01465	1.64	1.22–2.20	0.00098
IL-10	1.61	1.21–2.14	0.00096	1.45	1.06–1.98	0.02128
**IL-6**	**1.63**	**1.23–2.16**	**0.00071**	**1.57**	**1.17–2.12**	**0.00295**
IL-7	1.36	1.02–1.81	0.03334	1.16	0.85–1.59	0.34384
IL-8	1.41	1.06–1.86	0.0175	1.24	0.91–1.68	0.16608
KLRD1	1.36	1.02–1.80	0.03324	1.42	1.06–1.90	0.01999
MCP-3	1.41	1.07–1.87	0.01649	1.41	1.04–1.90	0.02674
MMP12	1.47	1.11–1.95	0.0079	1.50	1.12–2.01	0.00674
MMP7	1.40	1.05–1.85	0.02031	1.30	0.96–1.76	0.08433
NCR1	1.44	1.08–1.90	0.01195	1.56	1.16–2.10	0.00307
NOS3	1.42	1.07–1.89	0.01543	1.44	1.07–1.94	0.01659
PD-L1	1.61	1.21–2.15	0.001	1.53	1.12–2.10	0.00752
PGF	1.56	1.18–2.08	0.00194	1.59	1.17–2.15	0.00321
TIE2	1.36	1.03–1.80	0.03237	1.39	1.03–1.88	0.03387
**TNFRSF12A**	**1.72**	**1.29–2.29**	**0.00022**	**1.68**	**1.24–2.28**	**0.00079**
TNFRSF21	1.57	1.19–2.09	0.00167	1.40	1.04–1.88	0.02731
TNFRSF4	1.48	1.12–1.97	0.00629	1.37	1.01–1.87	0.04403
TNFSF14	1.36	1.03–1.81	0.03254	1.27	0.94–1.71	0.1166
VEGFR-2	0.70	0.53–0.93	0.01251	0.69	0.51–0.94	0.01856

c) Comparison of protein levels at first CT scan (approximately 3 months after start of treatment) vs. OS in univariate and multivariate analyses (including age, stage, baseline PS, baseline CA19-9, and type of palliative chemotherapy). Only proteins with statistically significant ($P < 0.05$) univariate analyses are shown.

	**Univariate analysis**			**Multivariate analysis**		
**Protein**	**HR**	**95% CI**	**P value**	**HR**	**95% CI**	**P value**
ADGRG1	1.52	1.12–2.06	7.0×10^{-03}	1.52	1.1–2.09	0.011
ANGPT2	1.72	1.26–2.33	5.8×10^{-04}	1.53	1.1–2.13	0.012
ARG1	1.45	1.07–1.97	0.018	1.19	0.86–1.64	0.305
CCL3	1.57	1.16–2.13	0.0030	1.65	1.17–2.33	0.0046
CCL23	1.77	1.30–2.41	0.00030	1.61	1.17–2.23	0.0036
CD244	1.43	1.05–1.93	0.022	1.45	1.03–2.02	0.031
CD4	1.79	1.31–2.42	0.00020	1.79	1.29–2.49	0.0049
CD40	1.75	1.28–2.38	0.00039	1.61	1.11–2.33	0.012
CD83	1.50	1.10–2.03	0.0092	1.43	1.03–1.99	0.031
CRTAM	1.69	1.24–2.31	0.00089	1.61	1.16–2.23	0.0044
**CSF-1**	**1.57**	**1.15–2.13**	**0.004**	**1.40**	**1.01–1.95**	**0.043**
CX3CL1	1.57	1.16–2.13	0.0037	1.64	1.14–2.35	0.0077
CXCL10	1.61	1.19–2.18	0.0021	1.47	1.03–2.09	0.033
CXCL11	1.49	1.10–2.02	0.010	1.33	0.95–1.87	0.098
CXCL12	1.64	1.21–2.23	0.002	1.38	0.99–1.93	0.061
**CXCL13**	**1.66**	**1.22–2.25**	**0.001**	**1.49**	**1.07–2.1**	**0.020**
CXCL9	1.45	1.07–1.97	0.016	1.42	0.99–2.04	0.059
DCN	1.46	1.08–1.97	0.015	1.52	1.09–2.13	0.015
GZMH	1.53	1.13–2.07	0.006	1.59	1.16–2.18	0.004
HGF	1.39	1.03–1.88	0.033	1.21	0.87–1.68	0.258
**IL-6**	**1.82**	**1.34–2.48**	**0.00014**	**1.62**	**1.16–2.26**	**0.0045**
IL-8	1.50	1.1–2.03	0.0093	1.63	1.17–2.27	0.0035
IL-10	1.66	1.22–2.25	0.0012	1.57	1.12–2.19	0.0091
IL-12RB1	1.52	1.12–2.06	0.0065	1.39	1.01–1.9	0.041

IL-33	1.37	1.01–1.86	0.040	1.21	0.87–1.67	0.257
MMP7	2.04	1.50–2.77	<0.0001	1.99	1.44–2.76	<0.0001
NOS3	1.36	1.01–1.84	0.045	1.27	0.91–1.76	0.159
PD-L1	1.69	1.24–2.29	0.00084	1.45	1.03–2.04	0.032
PGF	1.41	1.04–1.91	0.026	1.23	0.88–1.72	0.235
**TNFRSF12A**	**1.85**	**1.36–2.51**	**<0.0001**	**1.79**	**1.3–2.46**	**0.00038**
TNFRSF21	1.57	1.16–2.13	0.0033	1.56	1.11–2.21	0.011
TNFRSF9	1.49	1.10–2.02	0.010	1.33	0.96–1.83	0.090
TNFSF14	1.39	1.03–1.88	0.033	1.25	0.9–1.72	0.180

References

21. Enroth, S.; Berggrund, M.; Lycke, M.; Broberg, J.; Lundberg, M.; Assarsson, E.; Olovsson, M.; Stålberg, K.; Sundfeldt, K.; Gyllensten, U. High throughput proteomics identifies a high-accuracy 11 plasma protein biomarker signature for ovarian cancer. *Commun. Biol.* **2019**, *2*, 221. https://doi.org/10.1038/s42003-019-0464-9.
25. Altman, D.G.; McShane, L.M.; Sauerbrei, W.; Taube, S.E. Reporting recommendations for tumor marker prognostic studies (REMARK): Explanation and elaboration. *BMC Med.* **2012**, *10*, 51. https://doi.org/10.1186/1741-7015-10-51.
30. Vainer, N.; Dehlendorff, C.; Johansen, J.S. Systematic literature review of IL-6 as a biomarker or treatment target in patients with gastric, bile duct, pancreatic and colorectal cancer. *Oncotarget* **2018**, *9*, 29820–29841. https://doi.org/10.18632/oncotarget.25661.
31. Quatrini, L.; Mariotti, F.; Munari, E.; Tumino, N.; Vacca, P.; Moretta, L. The Immune Checkpoint PD-1 in Natural Killer Cells: Expression, Function and Targeting in Tumour Immunotherapy. *Cancers* **2020**, *12*, 3285. https://doi.org/10.3390/cancers12113285.
35. Badia-Villanueva, M.; Defaus, S.; Foj, R.; Andreu, D.; Oliva, B.; Sierra, A.; Fernandez-Fuentes, N. Evaluation of Computationally Designed Peptides Against TWEAK, a Cytokine of the Tumour Necrosis Factor Ligand Family. *Int. J. Mol. Sci.* **2021**, *22*, 1066. https://doi.org/10.3390/ijms22031066.
36. Yoriki, R.; Akashi, S.; Sho, M.; Nomi, T.; Yamato, I.; Hotta, K.; Takayama, T.; Matsumoto, S.; Wakatsuki, K.; Migita, K.; et al. Therapeutic potential of the TWEAK/Fn14 pathway in intractable gastrointestinal cancer. *Exp. Ther. Med.* **2010**, *2*, 103–108. https://doi.org/10.3892/etm.2010.181.
46. Friedman, J.H.; Hastie, T.; Tibshirani, R. Regularization Paths for Generalized Linear Models via Coordinate Descent. *J. Stat. Softw.* **2010**, *33*, 1–22. https://doi.org/10.18637/jss.v033.i01.
47. R Core Team. In Series. Available online: http://www.r-project.org/index.html (accessed on 12 April 2021).
48. Zhu, Y.; Knolhoff, B.L.; Meyer, M.A.; Nywening, T.M.; West, B.L.; Luo, J.; Wang-Gillam, A.; Goedegebuure, S.P.; Linehan, D.C.; DeNardo, D.G. CSF1/CSF1R Blockade Reprograms Tumor-Infiltrating Macrophages and Improves Response to T-cell Checkpoint Immunotherapy in Pancreatic Cancer Models. *Cancer Res.* **2014**, *74*, 5057–5069. https://doi.org/10.1158/0008-5472.can-13-3723.
49. Zhang, A.; Qian, Y.; Ye, Z.; Chen, H.; Xie, H.; Zhou, L.; Shen, Y.; Zheng, S. Cancer-associated fibroblasts promote M2 polarization of macrophages in pancreatic ductal adenocarcinoma. *Cancer Med.* **2017**, *6*, 463–470. https://doi.org/10.1002/cam4.993.
50. Stanley, E.R.; Berg, K.L.; Einstein, D.B.; Lee, P.S.; Pixley, F.J.; Wang, Y.; Yeung, Y.-G. Biology and action of colony-stimulating factor-1. *Mol. Reprod. Dev.* **1997**, *46*, 4–10. https://doi.org/10.1002/(sici)1098-2795(199701)46:1<4::aid-mrd2>3.0.co;2-v.
51. Rubio, A.J.; Porter, T.; Zhong, X. Duality of B Cell-CXCL13 Axis in Tumor Immunology. *Front. Immunol.* **2020**, *11*, 521110. https://doi.org/10.3389/fimmu.2020.521110.
52. Kazanietz, M.G.; Durando, M.; Cooke, M. CXCL13 and Its Receptor CXCR5 in Cancer: Inflammation, Immune Response, and Beyond. *Front. Endocrinol.* **2019**, *10*, 471. https://doi.org/10.3389/fendo.2019.00471.
53. Kumari, N.; Dwarakanath, B.S.; Das, A.; Bhatt, A.N. Role of interleukin-6 in cancer progression and therapeutic resistance. *Tumor Biol. J. Int. Soc. Oncodev. Biol. Med.* **2016**, *37*, 11553–11572. https://doi.org/10.1007/s13277-016-5098-7.

54. Mroczko, B.; Groblewska, M.; Gryko, M.; Kędra, B.; Szmitkowski, M. Diagnostic usefulness of serum interleukin 6 (IL-6) and C-reactive protein (CRP) in the differentiation between pancreatic cancer and chronic pancreatitis. *J. Clin. Lab. Anal.* **2010**, *24*, 256–261. https://doi.org/10.1002/jcla.20395.
55. Feng, L.; Qi, Q.; Wang, P.; Chen, H.; Chen, Z.; Meng, Z.; Liu, L. Serum levels of IL-6, IL-8, and IL-10 are indicators of prognosis in pancreatic cancer. *J. Int. Med Res.* **2018**, *46*, 5228–5236. https://doi.org/10.1177/0300060518800588.
56. Gudbrandsdottir, G.; Aarstad, H.H.; Bostad, L.; Hjelle, K.M.; Aarstad, H.J.; Bruserud, Ø.; Tvedt, T.H.A.; Beisland, C. Serum levels of the IL-6 family of cytokines predict prognosis in renal cell carcinoma (RCC). *Cancer Immunol. Immunother.* **2020**, *70*, 19–30. https://doi.org/10.1007/s00262-020-02655-z.
57. Laino, A.S.; Woods, D.; Vassallo, M.; Qian, X.; Tang, H.; Wind-Rotolo, M.; Weber, J. Serum interleukin-6 and C-reactive protein are associated with survival in melanoma patients receiving immune checkpoint inhibition. *J. Immunother. Cancer* **2020**, *8*, e000842. https://doi.org/10.1136/jitc-2020-000842.
58. Peltonen, R.; Gramkow, M.H.; Dehlendorff, C.; Osterlund, P.J.; Johansen, J.S.; Isoniemi, H. Elevated serum YKL-40, IL-6, CRP, CEA, and CA19-9 combined as a prognostic biomarker panel after resection of colorectal liver metastases. *PLoS ONE* **2020**, *15*, e0236569. https://doi.org/10.1371/journal.pone.0236569.
59. Tanaka, T.; Narazaki, M.; Kishimoto, T. IL-6 in Inflammation, Immunity, and Disease. *Cold Spring Harb. Perspect. Biol.* **2014**, *6*, a016295. https://doi.org/10.1101/cshperspect.a016295.
60. Uciechowski, P.; Dempke, W.C. Interleukin-6: A Masterplayer in the Cytokine Network. *Oncology* **2020**, *98*, 131–137. https://doi.org/10.1159/000505099.
61. Qiao, X.-W.; Jiang, J.; Pang, X.; Huang, M.-C.; Tang, Y.-L.; Liang, X.-H. The Evolving Landscape of PD-1/PD-L1 Pathway in Head and Neck Cancer. *Front. Immunol.* **2020**, *11*, 1721. https://doi.org/10.3389/fimmu.2020.01721.
62. Miao, Y.; Wang, J.; Li, Q.; Quan, W.; Wang, Y.; Li, C.; Wu, J.; Mi, D. Prognostic value and immunological role of PDCD1 gene in pan-cancer. *Int. Immunopharmacol.* **2020**, *89 Pt B*, 107080. https://doi.org/10.1016/j.intimp.2020.107080.
63. Gao, M.; Lin, M.; Moffitt, R.A.; Salazar, M.A.; Park, J.; Vacirca, J.; Huang, C.; Shroyer, K.R.; Choi, M.; Georgakis, G.V.; et al. Direct therapeutic targeting of immune checkpoint PD-1 in pancreatic cancer. *Br. J. Cancer* **2019**, *120*, 88–96. https://doi.org/10.1038/s41416-018-0298-0.
64. Perez, J.G.; Tran, N.L.; Rosenblum, M.G.; Schneider, C.S.; Connolly, N.P.; Kim, A.J.; Woodworth, G.F.; Winkles, J.A. The TWEAK receptor Fn14 is a potential cell surface portal for targeted delivery of glioblastoma therapeutics. *Oncogene* **2016**, *35*, 2145–2155. https://doi.org/10.1038/onc.2015.310.
65. Dwyer, B.J.; Jarman, E.J.; Gogoi-Tiwari, J.; Ferreira-Gonzalez, S.; Boulter, L.; Guest, R.V.; Kendall, T.J.; Kurian, D.; Kilpatrick, A.M.; Robson, A.J.; et al. TWEAK/Fn14 signalling promotes cholangiocarcinoma niche formation and progression. *J. Hepatol.* **2021**, *74*, 860–872. https://doi.org/10.1016/j.jhep.2020.11.018.
66. Donohue, P.J.; Richards, C.M.; Brown, S.A.; Hanscom, H.N.; Buschman, J.; Thangada, S.; Hla, T.; Williams, M.S.; Winkles, J.A. TWEAK Is an Endothelial Cell Growth and Chemotactic Factor That Also Potentiates FGF-2 and VEGF-A Mitogenic Activity. *Arter. Thromb. Vasc. Biol.* **2003**, *23*, 594–600. https://doi.org/10.1161/01.atv.0000062883.93715.37.
67. von Karstedt, S.; Conti, A.; Nobis, M.; Montinaro, A.; Hartwig, T.; Lemke, J.; Legler, K.; Annewanter, F.; Campbell, A.D.; Taraborrelli, L.; et al. Cancer Cell-Autonomous TRAIL-R Signaling Promotes KRAS-Driven Cancer Progression, Invasion, and Metastasis. *Cancer Cell* **2015**, *27*, 561–573. https://doi.org/10.1016/j.ccell.2015.02.014.
68. Ozawa, F.; Friess, H.; Kleeff, J.; Xu, Z.; Zimmermann, A.; Sheikh, M.; Büchler, M. Effects and expression of TRAIL and its apoptosis-promoting receptors in human pancreatic cancer. *Cancer Lett.* **2001**, *163*, 71–81. https://doi.org/10.1016/s0304-3835(00)00660-1.
69. Hoogwater, F.J.; Nijkamp, M.W.; Smakman, N.; Steller, E.J.; Emmink, B.L.; Westendorp, B.F.; Raats, D.A.; Sprick, M.R.; Schaefer, U.; van Houdt, W.J.; et al. Oncogenic K-Ras Turns Death Receptors Into Metastasis-Promoting Receptors in Human and Mouse Colorectal Cancer Cells. *Gastroenterology* **2010**, *138*, 2357–2367. https://doi.org/10.1053/j.gastro.2010.02.046.
70. Nahacka, Z.; Svadlenka, J.; Peterka, M.; Ksandrova, M.; Benesova, S.; Neuzil, J.; Andera, L. TRAIL induces apoptosis but not necroptosis in colorectal and pancreatic cancer cells preferentially via the TRAIL-R2/DR5 receptor. *Biochim. Biophys. Acta Mol. Cell. Res.* **2018**, *1865*, 522–531. https://doi.org/10.1016/j.bbamcr.2017.12.006.
